# Combination Immunotherapy in Pancreatic Cancer: Current Clinical Evidence and Mechanistic Rationale for Overcoming a “Cold” Tumor

**DOI:** 10.3390/cancers18183019

**Published:** 2026-09-17

**Authors:** Bode T. Eisenmenger, Abraham S. Rappaport, Liam H. Connell, Victor T. Petruc, Ethan J. Riordan, Mark R. Wakefield, Yujiang Fang

**Affiliations:** 1 Department of Microbiology, Immunology & Pathology, Des Moines University College of Osteopathic Medicine, West Des Moines, IA 50266, USA; 2 Department of Surgery, University of Missouri School of Medicine, Columbia, MO 65212, USA; asryhd@missouri.edu (A.S.R.); lc5pc@missouri.edu (L.H.C.); ejrcb8@missouri.edu (E.J.R.); wakefieldmr@health.missouri.edu (M.R.W.); 3 Ellis Fischel Cancer Center, University of Missouri School of Medicine, Columbia, MO 65212, USA

**Keywords:** pancreatic ductal adenocarcinoma, combination immunotherapy, immune checkpoint blockade, tumor microenvironment, biomarkers, clinical trials

## Abstract

Pancreatic ductal adenocarcinoma (PDAC) resists checkpoint blockades outside uncommon molecularly selected populations. This narrative review relates clinical combination studies to four overlapping resistance mechanisms: deficient antigenicity, presentation and priming; restricted effector-cell access; suppressive myeloid and regulatory-cell programs; and T-cell dysfunction. The grouping is an organizing heuristic, not a validated treatment-selection algorithm. A source audit through 5 September 2026 reconciled a nonexhaustive inventory of 85 supplied study/cohort entries and relevant additional reports; 84 inventory entries remain in the synthesis after the exclusion of 1 trial. Trial design, endpoint success, component attribution and pharmacodynamic evidence were assessed separately. PA.7 and CISPD3 did not improve their primary survival endpoints when checkpoint blockade was added to chemotherapy. Positive or apparently favorable findings require design-specific interpretation: NASCA changed several components, KG4/2015 pooled randomized and nonrandomized controls, and early vaccine signals have not established a routine benefit. POLAR did not meet its co-primary activity criteria; preliminary SWOG S2001 results did not demonstrate benefits from adding pembrolizumab to olaparib. dMMR/MSI-H remains an established tumor-agnostic selection biomarker, while exploratory PA.7 and tissue-state signatures require independent validation. Human immune changes, immunogenicity and target engagement should not be equated with clinical efficacy. Future trials should test defined component contributions, appropriate pharmacodynamic hypotheses and patient-reported outcomes, with sampling adapted to disease setting and feasibility.

## Simple Summary

Pancreatic cancer usually responds poorly to drugs that release the brakes on immune cells. Combining these drugs with other treatments may help, but early responses do not show which treatment contributed or whether patients live longer. This review examines clinical studies alongside four interacting problems: inadequate recognition of cancer, restricted access by immune cells, suppression by surrounding cells, and impaired immune function. These groups help organize questions for research; they are not a validated method of choosing treatment. We distinguish randomized comparisons from uncontrolled signals and immune changes from clinical benefit. Important limitations include selected patient groups, different treatment backbones, incomplete sampling, and uncertainty in some of the published results. Rare molecular features can identify patients eligible for established immunotherapy, but the benefit of adding another treatment remains uncertain. Future studies should test specific treatment contributions, measure the relevant biology when feasible, and assess symptoms, daily function, toxicity, and quality of life.

## 1. Introduction

Pancreatic ductal adenocarcinoma (PDAC) remains a major cause of cancer mortality. The American Cancer Society projects 67,530 new pancreatic cancer cases and 52,740 deaths in the United States in 2026. These are pancreatic-site statistics: five-year relative survival was 13% for diagnoses in 2015–2021, compared with 4% in the mid-1990s. Part of that increase reflects a greater proportion of neuroendocrine tumors, which have substantially better survival than ductal adenocarcinoma [1]. FOLFIRINOX, gemcitabine plus nab-paclitaxel, and NALIRIFOX improved survival against their respective comparators in metastatic disease [2,3,4]; their results do not define a universal chemotherapy benchmark. PDAC immune resistance includes deficient antigen presentation and priming, restricted T-cell access, suppressive myeloid populations, and dysfunctional effector cells [5]. Early ipilimumab and anti-PD-L1 trials reported no protocol-defined objective responses in their pancreatic cohorts, although one ipilimumab-treated patient had a delayed response [6,7]. Durvalumab with or without tremelimumab also failed its prespecified expansion criterion [8]. In contrast, pembrolizumab produced responses in 4 of 22 previously treated patients with dMMR/MSI-H pancreatic cancer [9]. Combination approaches seek to address complementary resistance mechanisms, but a biological rationale must be distinguished from evidence that an added component improves clinical outcomes [5,10].

Recent reviews address PDAC immune biology, therapeutic classes or pooled clinical outcomes [11,12,13,14,15,16]. Our contribution is a narrative comparison of the clinical contrast tested, the proposed function of each component and the measurements supporting that function. 

## 2. Literature Search and Study Appraisal

This narrative review uses a curated clinical inventory and design-specific critical discussion. It does not provide a systematic review, meta-analysis or pooled treatment-effect estimate.

### 2.1. Databases, Dates and Search Strategy

The supplied working review documented PubMed/MEDLINE queries through 29 August 2026, English-language restrictions, registry searches, and supplementary citation and conference searches. The query strings and reported flow counts are retained in Supplementary Sections S1–S3 as a historical account. Supplementary Tables S1 and S2 present the historically reported exclusion categories and counts for title/abstract screening and later eligibility assessment, respectively. Record-level screening logs and parts of the reference-set provenance were unavailable, so that account cannot be independently reconstructed. The present revision checked primary publications, corrections, accessible supplements and registries through 5 September 2026. This source audit and targeted update searching are distinct from the earlier search; they do not validate its historical retrieval or screening counts.

The inventory is nonexhaustive. Additional relevant studies were located during the revision, and database, language and source-access limitations remain. Supplementary Table S3 identifies values that could not be established from the source material obtained, whereas Supplementary Table S4 identifies specific unresolved details in inspected reports. 

### 2.2. Eligibility Criteria

The supplied tabulation generally selected prospective interventional pancreatic-cancer combinations published from 2013 onward, with at least 10 pancreatic patients and clinical efficacy results. Scope exceptions and analysis populations are specified by entry. It includes immune checkpoint, costimulatory, vaccine, oncolytic, innate, cellular and selected stromal or myeloid interventions. These criteria describe the tabulation scope, rather than a demonstrated exhaustive selection process. Relevant smaller studies, monotherapy platforms and mechanistic literature are discussed separately. Mixed histology and analysis populations are identified where material.

### 2.3. Study Selection

Supplementary Table S5 retains 84 of the 85 supplied study/cohort entries after exclusion of 1 trial. These entries are supported by 95 listed reports: 84 index reports, 5 linked analyses and 6 notices. These 84 study/cohort entries do not represent 84 independent trial protocols. Related phases and platform cohorts share participants or controls, including PRINCE, COMBAT and MORPHEUS. Newly identified qualifying combination reports and smaller or monotherapy contextual reports are distinguished from this fixed inventory. No total unique-patient count or cross-study tissue-measurement percentage is presented.

The earlier review described a single assessor; independent human screening or consensus adjudication is not documented. The current source checks address reported study characteristics and interpretation, not the reliability of that historical screen. Source-access limits and unresolved fields remain explicit.

### 2.4. Descriptive Appraisal Tools

Author-defined tiers describe design separately from quality, maturity, endpoint success and attribution. They are not Oxford levels or validated risk-of-bias scores [17].

T1 denotes a randomized, prespecified comparative clinical-efficacy hypothesis; T2 denotes randomized exploratory, noncomparative arm-benchmark or primarily biological/safety studies; T3 denotes prospective nonrandomized clinical studies, with or without a control; and T4 denotes preclinical or translational evidence without clinical efficacy assessment. Mixed designs are labeled. Phase, statistical power, early stopping and whether a comparator isolates a component are reported separately.

Descriptive flags identify: (a) limitations of a same-backbone component comparison; (b) historical or external comparison; (c) outcome-derived, post hoc or outcome-selected exploratory analysis; (d) selected or incomplete analysis populations; (e) a specific unresolved reporting discrepancy; (f) conference abstracts or non-peer-reviewed publications; (g) mixed histology or tumor type. Flags apply to the stated inference, but not automatically to every result in a study. Online-first peer-reviewed reports do not receive flag f solely for lacking issue pagination. Each row identifies the component or analysis affected. An active immune-containing comparator can isolate an added component; expected all-treated safety and measurable-disease response sets do not automatically receive flag d.

No formal instrument-based risk-of-bias assessment was performed. Relevant concerns include randomization and missing outcomes in trials, as well as confounding, selection and outcome ascertainment in nonrandomized studies. RoB 2, ROBINS-I and JBI or MINORS provide design-specific appraisal approaches; the descriptive scheme does not replace them [18,19,20,21]. SANRA informed the narrative review appraisal, rather than serving as a reporting guideline or evidence-certainty grade [22].

### 2.5. Controlled Vocabulary for Regimen-Level Evidence Claims

Mechanistic plausibility, an early clinical signal, and demonstrated comparative benefit are distinct. Uncontrolled or outcome-selected findings are described as hypothesis-generating; a favorable randomized result is interpreted for its actual endpoint and treatment contrast. “Investigational” describes clinical readiness, independently of tier. Biomarker roles are defined in Section 7.

### 2.6. Tools Used in the Literature Discovery

AI-assisted tools were used to locate candidate studies, to execute and document the search, and to check reported study characteristics and reference metadata against the primary publications. The full scope, each tool, and its version are declared in the Acknowledgments section. Screening, eligibility, tier and flag decisions were made by the author-assessor, and every study characteristic, numerical result and design judgment was taken from the primary publication or registry record, where neither supplies the value that the cell indicates rather than carrying an inference.

## 3. Immunobiology of the “Cold” PDAC Tumor

PDAC contains heterogeneous immune states across patients, anatomical sites and regions within a tumor [23,24,25]. We organize resistance into four overlapping groups: deficient antigenicity, presentation and priming; restricted effector-cell access; suppressive myeloid and regulatory-cell programs; and T-cell dysfunction [5,26]. These groups are used to appraise combinations, rather than assign a clinical subtype.

### 3.1. Insufficient Antigenicity, Presentation, and Priming

Mutation burden alone does not establish immunogenicity: expression, processing, presentation and T-cell recognition determine whether a mutation becomes a useful target [27]. In a longitudinal analysis of 70 pancreatic cancers, a neoantigen-quality model predicted clonal evolution, and recurrent tumors in long-term survivors contained fewer high-quality neoantigens [28]. These observations concern immune selection and prognosis; they do not validate a combination-treatment selector.

Presentation can fail despite antigen availability. Experimental PDAC systems demonstrate autophagy-dependent MHC-I degradation [29]; hypoxia also suppresses presentation in experimental colorectal models [30]. Dendritic-cell paucity and dysfunction impair immune surveillance in the neoantigen-expressing murine PDAC [31]. These findings motivate antigen-supplying or priming interventions, with component-specific measurements of tumor recognition.

### 3.2. Matrix, Vascular, and Chemokine-Mediated Exclusion

Matrix accumulation impairs vascular function and drug delivery in mouse PDAC [32,33], while stellate-cell and fibroblast chemokine programs can redirect lymphocytes away from malignant glands [34,35]. CAF states have distinct functions: depletion of selected fibroblasts, collagen or hedgehog-dependent stroma can accelerate disease [36,37,38,39,40]. This supports the selective modification of exclusionary functions while considering tumor-restraining elements [41].

CXCL12–CXCR4 and CXCR2-dependent programs affect immune access in murine PDAC [34,42]; human CAF cocultures also modify T-cell chemokine receptor expression [43]. In a human spatial-imaging cohort, cytotoxic T-cell proximity to cancer cells correlated with survival, whereas bulk collagen-I and αSMA-positive fibroblast content did not correlate with T-cell paucity [44].

Direct tumor measurements demonstrate hypoxia in human pancreatic cancer [45]. Hypoxia and poor perfusion provide a rationale for physiologic conditioning, but improved oxygenation or delivery alone cannot establish restored antitumor immunity [46].

### 3.3. Suppressive Myeloid and Regulatory-Cell Programs

KRAS-driven GM-CSF promotes suppressive myeloid recruitment in experimental PDAC [47,48]. Recruitment pathways compensate: increased tumor neutrophils followed CCR2 inhibition in patients, and combined CCR2/CXCR2 targeting improved activity in mouse models [49]. CSF1R blockade and subset-depletion studies likewise demonstrate context-dependent immune effects [50,51]. The CXCL1–CXCR2–TNF/TNFR2 circuitry connects myeloid recruitment, stromal inflammation and T-cell dysfunction, with therapeutic causality established experimentally [52].

Macrophage or myeloid abundance alone cannot establish suppressive function [49,50].

### 3.4. T-Cell Dysfunction and Adaptive Resistance

Persistent stimulation can establish dysfunctional T-cell states, including TOX-dependent transcriptional and epigenetic programs demonstrated in mice [53]. Inhibitory receptors also mark activation or adaptation [54,55]. PDAC tissue studies document PD-1, LAG-3, TIGIT and related pathways, but expression and prognostic associations do not establish treatment-predictive performance [56,57,58].

### 3.5. The Four-Barrier Scheme: Status, Justification and Limits

Single-cell and spatial studies identify diverse malignant, fibroblast and immune states [38,59,60]. Distinct sub-microenvironments can coexist within one tumor [61], limiting bulk classifications. These findings help formulate assayable hypotheses; they do not currently provide a universal dominant-barrier score.

The four groups condense mechanisms already represented in the cancer–immunity cycle and immune-inflamed, excluded and desert descriptions [62,63,64]. Their purpose here is to connect each clinical comparison to a proposed component function and the evidence measuring it. Assignments overlap: CXCL1–CXCR2 signaling links myeloid recruitment with spatial exclusion [52]. The scheme has not been prospectively validated for treatment selection.

The cancer–immunity cycle describes linked processes, immunotypes summarize spatial patterns, and molecular classifications emphasize malignant-cell programs [59,60,62,63,64]. The four groups connect these perspectives to testable component functions.

**Figure 1:**
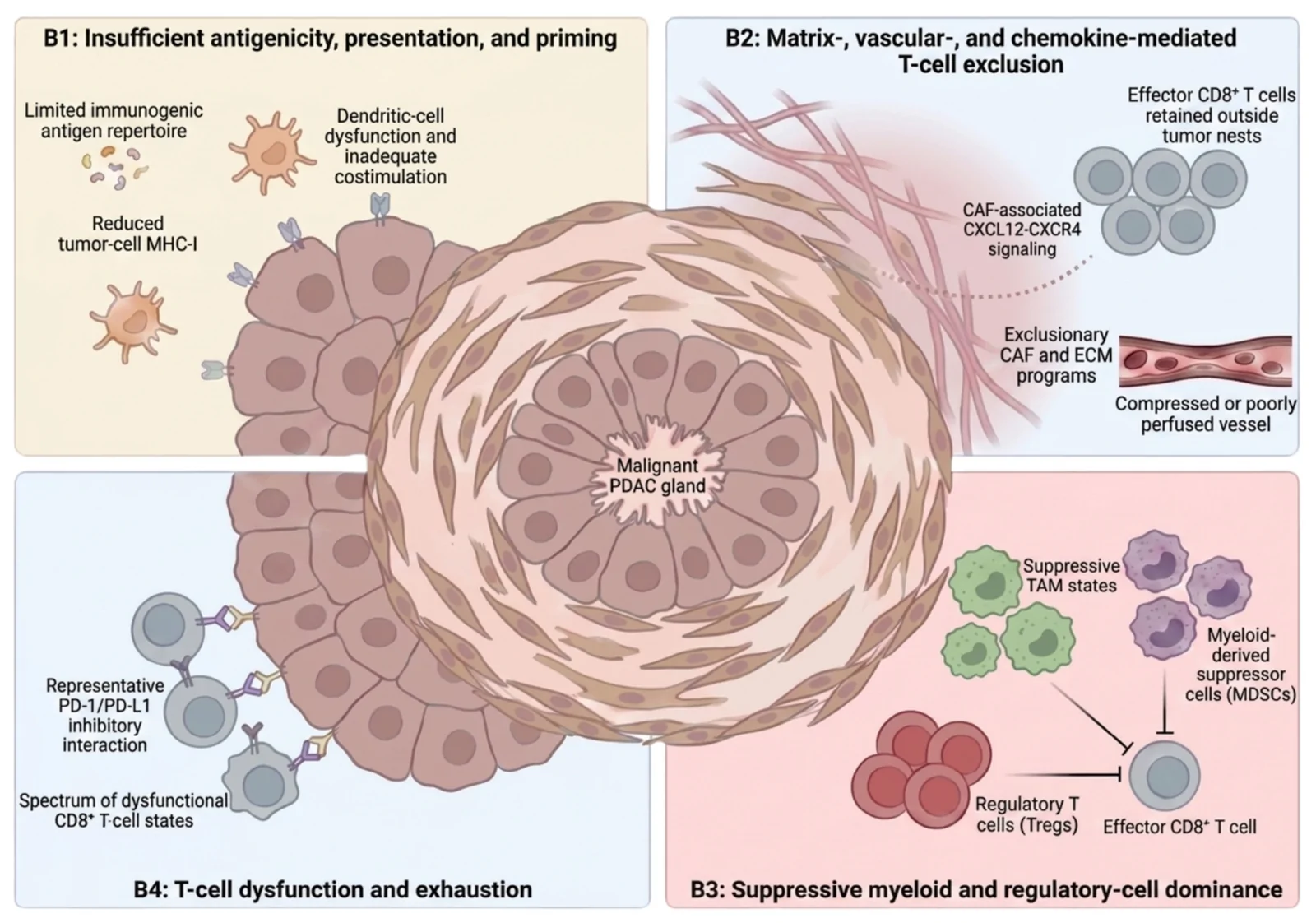
Four overlapping groups of immune resistance in pancreatic ductal adenocarcinoma: insufficient antigenicity, presentation and priming (**B1**); matrix-, vascular- and chemokine-associated exclusion (**B2**); suppressive myeloid and regulatory-cell programs (**B3**); T-cell dysfunction (**B4**). This conceptual scheme organizes evidence appraisal; it is not a validated clinical classification or a ranking of causal dominance.

DC cross-priming is rate-limiting in particular murine PDAC models and can be restored by CD40 agonism, alone or with checkpoint blockade [65,66]. Other models require additional costimulatory or inhibitory-pathway interventions for durable control [67,68]. The effects of stromal depletion vary with the targeted population [36,37,40]. These experiments identify context-specific dependencies, not a fixed hierarchy of barriers in patients. These four overlapping groups of immune resistance are summarized in Figure 1.

## 4. Overview of Immunotherapy Modalities Relevant to Combinations

Combination components may supply antigen, support priming, alter access or suppressive programs, or sustain effector function. Table 1 summarizes overlapping activities and human evidence without ranking efficacy.

Stromal, trafficking and myeloid agents may alter access or suppressive functions [69,70,71,72]. Cellular approaches can supply effectors but remain constrained by recognition, persistence and the microenvironment [73,74,75]. Nominal target biology should therefore be distinguished from the effect measured at a clinical dose.

**Table 1:** Functional roles of modalities used in PDAC immunotherapy combinations.

Modality or Component	Intended Function	Barrier Link	Human Evidence	Interpretive limitation
PD-1/PD-L1 blockade	Support tumor-reactive T-cell function	B4; overlapping roles	T1–T3 [8,76]	Limited activity in unselected PDAC; molecular selection matters.
CTLA-4 blockade	Modify priming, regulatory and effector responses	B1/B3/B4	T1–T3 [76,77]	Incremental benefit and immune toxicity require an actual component comparison.
CD40 agonism	Activate antigen-presenting cells and myeloid programs	B1/B3	T2/T3 [78,79,80]	Pharmacodynamics and uncontrolled response do not establish an added benefit.
Vaccines	Generate antigen-specific immunity	B1	T1–T3 [81,82,83,84,85,86]	Large trials generally negative; KG4 pooled randomized and nonrandomized controls.
Oncolytic viruses	Local replication, antigen release and inflammation	B1/B2	T1–T3 [87,88,89]	Antiviral responses and delivery do not establish tumor-directed immunity.
Innate agonists	Activate innate sensing and priming	B1/B3	T2 human [90]; T4 models [91,92]	Separate experimental STING/TLR rationale from human IMM-101 evidence.
Chemotherapy	Cytotoxic backbone; schedule-dependent immune effects	B1/B3	Backbone in T1–T3 combinations	Immune contribution requires a suitable comparison.
Radiotherapy	Local control; antigen and innate effects	B1/B2/B3	T2/T3 [93,94]	Systemic activity differs from response inside irradiated lesions.
FAK/matrix/CAF strategies	Modify stromal and immune programs	B2; experimental B1 after FAK depletion	T3 [95]	Kinase inhibition does not reproduce all effects of FAK depletion [70].
Hyaluronan-directed strategies	Alter matrix and access	B2	T2/T3 [96,97]	Limited activity; on-treatment substrate modification not established.
CXCL12–CXCR4 blockade	Alter immune-cell distribution	B2/B3	T2/T3 [98,99,100]	Cell accumulation does not identify trafficking versus local expansion.
Myeloid/suppressive-pathway agents	Alter recruitment or cell function	B3; overlapping B1/B2	T1–T3 [101,102,103,104]	Markers of abundance do not establish a suppressive function.
Adenosine-pathway agents	Reduce adenosine-associated suppression	B3/B4	T2/T3 [105,106]	Shared-agent randomization does not isolate that agent.
Cellular therapies	Supply or redirect effector cells	B1/B4; access constraints	Randomized and nonrandomized [73,107,108]; some prospective design details remain unresolved	Manufacturing, prior-treatment selection and persistence matter.
Bispecific T-cell engagers	Redirect T-cell recognition	B1/B4	Platform context [75]	A review-level class description does not establish a PDAC combination tier.

Table 1 notes: B1–B4 refer to the overlapping groups in Section 3. Tiers describe the study design (Section 2.4), not efficacy or clinical readiness. Mechanisms established in the experimental models are distinguished from the human pharmacodynamic findings.

## 5. Clinical Evidence: Combination Immunotherapy in PDAC

The nonexhaustive supplementary inventory contains 84 retained study/cohort entries; shared cohorts and heterogeneous population definitions prevent a defensible unique-patient total. Clinical comparisons should be read within their disease setting, treatment line and analysis population. Table 2 selects informative studies; Supplementary Table S5 supplies a broader inventory and report-specific limitations. Responses with an active chemotherapy backbone, or survival relative to historical experience, alone do not identify an immune component’s contribution. Published reviews and quantitative syntheses provide complementary summaries with differing scopes; they do not replace interpretation of each trial’s treatment contrast [15,16,109].

### 5.1. Chemoimmunotherapy Combinations

PA.7 provides a randomized test of adding durvalumab and tremelimumab to gemcitabine/nab-paclitaxel in 180 previously untreated patients with metastatic PDAC. The 2026 final analysis, at an 81.6-month median follow-up, confirmed no significant OS improvement: 9.84 versus 8.76 months (HR 0.89, 95% CI 0.64–1.22); PFS was also similar [76,110]. The 2022 publication was the primary analysis. The trial targeted an OS HR of 0.65 with 80% power at a two-sided 10% significance level; the final report’s 95% CI includes both benefit and harm and does not establish equivalence. Two grade 5 events were considered at least possibly immunotherapy-related [76]. The result applies to the tested dose and schedule in an unselected population; it does not identify whether alternative sequencing or biomarker selection would change activity.

PRINCE tested each arm against a 35% historical 1-year OS benchmark rather than through a powered between-arm comparison. Only nivolumab/chemotherapy met that criterion (57.7%); sotigalimab/chemotherapy and the triplet did not [79]. Its 105-patient efficacy set included 93 randomized and dosed phase 2 patients plus 12 phase 1b patients. The absent chemotherapy-only arm and historical benchmark limit attribution of benefits. Paired tumor analyses in 14 patients and exploratory blood correlatives identify mechanistic associations but do not validate biomarkers or establish incremental benefits over chemotherapy [78,79]. Differences in eligibility, supportive care, post-protocol treatment and assessment era can alter a historical survival benchmark; these factors were not balanced against a randomized chemotherapy-only arm.

CISPD3 directly tested sintilimab added to mFOLFIRINOX and failed its primary OS endpoint despite a higher reported ORR [111]. NASCA met its randomized ORR endpoint (51.1% versus 24.4%) and showed longer PFS, while OS remained uncertain (HR 0.77, 95% CI 0.47–1.28) [112]. NASCA changed the chemotherapy backbone and added both camrelizumab and surufatinib, so it cannot isolate the PD-1 contribution. Its immune analyses used baseline tissue from 26 experimental-arm patients. Uncontrolled checkpoint–chemotherapy studies similarly cannot establish the checkpoint contribution [113,114,115,116,117,118]. In the neoadjuvant mFOLFIRINOX/nivolumab pilot, 22 of 28 treated patients underwent resection, and a median OS of 35 months was estimated in 26 patients with completed treatment and available records [115]. Five paired tumor RNA sets inform treatment-associated biology, while conditional survival populations and external comparisons limit clinical attribution.

OPTIMIZE-1 met its primary response criterion in 57 patients who received at least two cycles; 70 patients were treated overall [80]. The full update reported ORR 24/57 (42.1%) and a median OS of 14.9 months, alongside a sensitivity analysis including all 65 RP2D patients with ORR 24/65 (36.9%) and an OS of 14.0 months [119]. Eight RP2D patients discontinued early, including for progression, toxicity or clinical deterioration. Both populations should therefore accompany interpretation of the response signal. The three evaluable paired tumor biopsies were all from responders. These clinical and pharmacodynamic findings support controlled testing without establishing mitazalimab’s incremental benefit [119].

Other CD40 studies provide blood pharmacodynamics and limited tissue observations. CP-870,893 plus gemcitabine produced four partial responses and peripheral immune activation [120]. Neoadjuvant selicrelumab yielded T cell-enriched tissue in 9 of 11 resections compared with external untreated or chemotherapy/chemoradiation cohorts, without paired pre-treatment tumors [121]. IMAGE-1’s randomized safety-focused comparison of IMM-101 plus gemcitabine showed an uncertain overall OS effect (HR 0.68; 95% CI, 0.44–1.04); favorable metastatic-subgroup findings require caution alongside the small locally advanced subgroup’s opposite result [90]. No on-treatment immunologic assay was reported in that study.

Recent myeloid and stromal combinations illustrate the distinction between pharmacodynamic activity and clinical attribution. ARC-8 randomized zimberelimab addition to quemliclustat/chemotherapy; its apparent OS advantage for pooled quemliclustat-treated patients came from a propensity-matched external comparison [105]. BMS-813160 reported paired tumor changes, including increased CD163-positive cell density in 76% of BMS-treated patients, but clinical comparisons used treated populations and were affected by differential pre-treatment attrition [103]. CD163 density alone does not establish a suppressive function. In daNIS-1, paired tumor RNA analyses supported TGF-β and CAF pathway suppression without improved clinical outcomes [122]. These observations show that the measured pharmacodynamic changes did not establish clinical benefit in these studies.

### 5.2. Immunotherapy Plus Radiotherapy

Radiotherapy can provide local tumor control and alter antigen release, innate signaling, and immune-cell composition. These effects depend on dose, fractionation and tissue context, as illustrated by preclinical experiments and human tissue studies [123,124,125]. Clinical combinations therefore need to distinguish local radiation effects from systemic activity. The metastatic studies of Xie and Parikh assessed responses outside irradiated lesions, an appropriate way to address that question [94,126]. Xie reported one confirmed and one unconfirmed partial response among 39 response-evaluable patients; survival analyses included 58 treated patients. Parikh reported three responses among all 25 patients with PDAC. Restricting the latter analysis to 17 patients who reached radiation increased the response estimate, making the intention-to-treat result the more informative summary [126].

Randomized designs have addressed different components of this strategy. Katz et al. compared neoadjuvant chemoradiotherapy with or without pembrolizumab in 37 patients, estimating safety and intratumoral CD8-cell density rather than powering a clinical efficacy comparison [127]. Resection-tissue assays were available in smaller, panel-specific subsets; these limitations do not imply that early discontinuations were omitted from survival analysis. CheckPAC randomized 88 patients to stereotactic body radiotherapy and nivolumab with or without ipilimumab, with 84 initiating treatment [93]. The dual-checkpoint arm met its clinical-benefit screening criterion and had six partial responses, versus one with nivolumab alone. Median overall survival was 3.8 months in both arms. Each arm was assessed independently, so the study provides a signal for further testing without establishing comparative survival benefit or the contribution of radiation.

Further intensification has shown limited activity. TRIPLE-R reported no objective response against a prespecified 15% expansion threshold in 26 patients receiving tocilizumab, ipilimumab, nivolumab and stereotactic radiotherapy [128]. Median OS was 5.3 months (95% CI, 2.3–8.0). CA209-9KH evaluated nivolumab and stereotactic radiotherapy in 15 patients whose nonmetastatic disease remained controlled after four cycles of FOLFIRINOX; its survival estimates begin at study entry after induction and are not directly comparable with estimates measured from initial chemotherapy [129].

THAG reported a 51.8% response rate in 56 patients with borderline resectable or locally advanced disease; 22 underwent resection, of whom 20 had an R0 resection [130]. This single-arm result supports further evaluation, with surgical selection and overlapping earlier reports explicitly accounted for. Across these studies, controlled efficacy estimates, out-of-field responses and tissue pharmacodynamics answer complementary questions; none should substitute for the others.

### 5.3. Immunotherapy Plus Stromal, Trafficking, or Myeloid-Targeted Therapy

The CXCL12–CXCR4 blockade has produced human pharmacodynamic evidence alongside modest clinical activity. COMBAT cohort 1 combined motixafortide with pembrolizumab without chemotherapy and reported increased tumor CD8-cell infiltration [98]. Its separate chemotherapy cohort added nanoliposomal irinotecan and fluorouracil/leucovorin, with a confirmed response rate of 13.2% [99]. These cohorts share a protocol but contain distinct participants; the initial chemotherapy report overlaps with the later full cohort. Plerixafor plus cemiplimab produced no objective response among 21 patients, although 2 had stable disease, including one for nine months [131]. Fourteen paired tumor biopsies showed increased immune-cell density and altered spatial organization, with macrophage-associated suppressive programs. These findings support biological activity but do not directly distinguish trafficking from local expansion or prove the human mechanism of resistance.

MORPHEUS-PDAC compared experimental atezolizumab combinations with concurrent chemotherapy controls. The motixafortide arm had no objective response, and the non-PEGPH20 arms closed after a preliminary evaluation for limited activity [100]. Atezolizumab plus PEGPH20 produced four responses among 66 treated patients, versus one among 42 shared chemotherapy controls, without a persuasive progression-free or overall-survival signal [96]. The separate eight-patient PEGPH20–pembrolizumab study also reported no objective response [97]. The limited activity informs clinical development; the absence of demonstrated on-treatment hyaluronan depletion separately limits mechanistic interpretation. Shared MORPHEUS controls and later treatment crossovers must be counted once.

FAK-directed treatment also requires the separation of pharmacology from broader target biology. Defactinib, pembrolizumab and gemcitabine produced paired tumor changes in a small phase I study, including reduced phosphorylated FAK and increased CD8 infiltration [95]. Disease control in refractory and maintenance cohorts is descriptive, particularly after selection for prior chemotherapy control. The published correction removes erroneous heart-failure attribution to study drugs [95]. Preclinical restoration of antigen presentation after FAK depletion was kinase-independent and was not reproduced by the tested FAK kinase inhibitors; it should not be presented as an established action of clinical defactinib [70].

Direct myeloid modulation has supplied both tissue evidence and controlled clinical tests. PF-04136309 plus FOLFIRINOX reduced tumor-associated macrophages in six paired biopsies and produced responses in 16 of 33 evaluable patients; its small nonrandomized control group cannot establish the added clinical effect [132]. In the neoadjuvant BMS-813160 study, 38 tumor specimens came from 24 patients, rather than 38 paired biopsies [133]. Only the initial allocation was randomized; subsequent expansion was single-arm. Reduced intratumoral monocytes and macrophages therefore support pharmacodynamic activity, while the eight chemotherapy controls provide limited comparative precision. Canakinumab, spartalizumab and chemotherapy reduced circulating monocytic suppressor cells in ten patients and increased the intratumoral CD8:Treg ratio in six paired samples, with smaller changes in tumor myeloid populations [134]. Clinical findings remain heterogeneous. Entinostat plus nivolumab missed its response endpoint despite responses in 3 of 27 evaluable patients (11.1%; 95% CI, 2.4–29.2) and informative lead-in tissue sampling [101]. Galunisertib plus durvalumab established a recommended dose with limited activity [102]. Galunisertib plus gemcitabine met its prespecified Bayesian survival criterion, but the result was sensitive to historical-control borrowing and warrants confirmation [104]. Randomized oleclumab and acalabrutinib combinations did not establish a clinical advantage, while exploratory biomarker results remain candidates for validation [106,135]. Lenvatinib plus pembrolizumab produced a 7.8% response rate among 103 patients, with grade 3–4 treatment-related adverse events in 59.2% [136]. These results favor component-specific development with adequate controls, pharmacodynamic sampling and tolerability assessment.

### 5.4. Dual, Multi-Agent and Vaccine Combinations

Dual checkpoint blockade has shown limited activity in unselected PDAC [8,137]. REVOLUTION cohort A met its stage-1 activity criterion but did not proceed to expansion, so its signal does not establish benefit over chemotherapy [138].

Vaccines can generate antigen-specific T cells, but immunogenicity does not establish clinical benefit. Autogene cevumeran induced neoantigen responses in 8 of 16 vaccinated patients; the association with longer recurrence-free survival persisted in a landmark analysis after the priming series and at an extended follow-up [83,139]. The landmark analysis addresses a specific guarantee-time concern but cannot remove prognostic differences between immune responders and nonresponders. A pooled mutant KRAS vaccine with nivolumab and ipilimumab induced tumor-matched responses in 10 of 12 patients; survival comparisons used a small, outcome-associated immune subgroup and remain exploratory [84,84]. Vaccine-expanded clones were found in selected tissue. The autogene liver lesion contained a rare matched TP53 signal but no histologically demonstrable malignant cells, so it was a putative micrometastasis rather than a confirmed tumor-response biopsy. In the GVAX/CRS-207 program, an early randomized survival signal was followed by negative studies; adding nivolumab did not improve survival, and GVAX plus ipilimumab maintenance was inferior to continued chemotherapy [77,140,141,142,143,144]. Tumor recruitment of antigen-specific clones can coexist with myeloid enrichment and little clinical activity [145].

Larger randomized trials reinforce the distinction between immune induction and efficacy. TeloVac enrolled 1062 patients and found no overall-survival improvement with either sequential or concurrent GV1001 plus gemcitabine–capecitabine [81]. Algenpantucel-L did not improve survival in borderline resectable or locally advanced disease despite encouraging earlier uncontrolled adjuvant results, and elpamotide also failed its primary survival endpoint [82,86,146]. Peripheral immune assays do not resolve whether these treatments induced an adequate response within tumors. TeloVac measured blood and skin responses; elpamotide assessed a peripheral cytotoxic-lymphocyte gene panel without a significant post-vaccination increase [81,86]. WT1 and survivin2B peptide-vaccine studies also linked post-treatment immune measures to outcomes in selected subsets, without establishing that these associations predicted a randomized treatment benefit [147,148]. An earlier single-arm WT1–gemcitabine study is additional clinical context outside the fixed inventory and does not establish a randomized vaccine contribution [149].

KG4/2015 reported longer overall survival with GV1001 plus gemcitabine–capecitabine, but its published comparison combines randomized and nonrandomized patients [85,85]. Of 111 patients with eotaxin above 81.02 pg/mL, 75 were randomized to vaccination and 36 to chemotherapy; another 37 eotaxin-low patients entered the control group without randomization. The reported median survival of 11.3 versus 7.5 months (*p* = 0.021) compares the vaccine group with these pooled controls. Differential withdrawal and the use of a copula-based survival estimator further require attention to censoring assumptions. A high-eotaxin randomized comparison was planned as secondary, but its separate effect estimate was not located in the obtained report and supplements. This signal warrants independent evaluation with a contemporary backbone and a prespecified biomarker comparison; it does not yet establish the vaccine contribution in a randomized biomarker-selected population.

Cellular combinations remain early in development. In TACTOPS, 13 patients with disease controlled by chemotherapy received nonengineered multiantigen-specific T cells while continuing standard treatment; three responded, and selected blood and tumor analyses demonstrated persistence and tumor-associated clonotypes [73]. Prior disease control, concurrent chemotherapy and procurement-to-infusion selection limit attribution, and the prespecified infusion-feasibility criterion was not met. Randomized electroporation plus Vγ9Vδ2-cell therapy reported longer median survival, but incompletely reported effect precision and inconsistent adverse-event counts constrain interpretation [107]. Its immune measurements were peripheral, and staff-rated performance status should not be called patient-reported quality of life. The randomized S-1/CIK report did not establish an overall-survival benefit [108]. NK-cell and adjuvant γδ-cell studies support feasibility with selected clinical signals, while their evaluability and manufacturing restrictions require explicit denominators [150,151].

### 5.5. Maintenance and PARP-Inhibitor Combinations

Maintenance studies select patients whose disease has already remained controlled under chemotherapy. In the randomized niraparib study, each checkpoint-inhibitor arm was evaluated separately against a prespecified six-month progression-free survival benchmark [152,152]. Niraparib–ipilimumab met that benchmark in the per-protocol population, but its intention-to-treat confidence interval did not exclude the null benchmark; niraparib–nivolumab did not meet it. The design did not formally compare the two arms and included no PARP-inhibitor-alone control. These findings support further testing, while platinum sensitivity, analysis exclusions and immune toxicity limit attribution of benefit to the CTLA-4 blockade.

POLAR evaluated pembrolizumab–olaparib in three nonrandomized maintenance cohorts [153]. In the core-HRD cohort, the reported response rate was 7 of 20 patients with measurable disease (35%), and six-month progression-free survival was 64%; the study reported that neither co-primary endpoint was met. The protocol’s 43% response and 77% progression-free survival values were design alternatives, rather than the final decision boundaries: the planned cohort of 33 required at least 12 responses or 23 patients progression-free at six months. Central response assessment is reported as RECIST 1.1, whereas the protocol endpoint description specifies iRECIST, an inconsistency that should be clarified without treating the reported response rate as wholly undefined. Platinum selection, mixed histology and the absence of a control prevent attribution to pembrolizumab. Baseline HRD and immune correlates are exploratory associations, not validated predictors of added checkpoint benefit.

SWOG S2001 provides a randomized test of pembrolizumab addition to olaparib in germline BRCA1/2-mutated disease [154]. The planned interim analysis included 68 eligible patients and led to closure for futility: median progression-free survival was 8.2 versus 6.4 months (*p* = 0.82). The design targeted a hazard ratio of 0.6 with 80% power; the conference abstract reports no treatment-effect HR or confidence interval, limiting assessment of the precision of this preliminary negative result. Overall-survival follow-up remains immature, and the conference report does not establish added clinical benefit. In pre-treated disease, a 36-patient, four-arm study of CBP501, cisplatin and nivolumab used activity rules for individual arms; its small numerical differences cannot establish a CBP501 contribution [155]. DAPPER found limited clinical activity and no significant increase in intratumoral T cells with durvalumab plus olaparib or cediranib [156]. The earlier CBP501 phase Ib conference report is also contextual; its pancreatic expansion and efficacy populations differ from later summaries [157].

**Table 2:** Selected clinical studies of combination immunotherapy in pancreatic cancer.

Study (Registry)	Setting; Line	Regimen vs. Comparator	n	Tier	Primary Endpoint and Result	Key Outcome	Human Biological Measurements	Flags
Chemotherapy-based combinations								
PA.7 [76,110]; NCT02879318	Metastatic; 1L	Gem/nab-P ± durvalumab/tremelimumab	180 randomized	T1	OS: no	OS HR 0.89 (95% CI 0.64–1.22)	Baseline plasma and archival tissue; no reported on-treatment immune assay	c
NASCA [112]; NCT05218889	Advanced; 1L	Camrelizumab/surufatinib/nab-P/S-1 versus Gem/nab-P	90 randomized	T1	ORR: yes	ORR 51.1% versus 24.4%; OS uncertain	Baseline tissue subset; multiple treatment components changed	a; c
CISPD3 [111]; NCT03977272	Metastatic/recurrent; 1L	mFOLFIRINOX ± sintilimab	110 randomized	T1	OS: no	Higher secondary ORR; no primary OS benefit	Immune-assay reporting unresolved from the accessed abstract/registry	None assigned
PRINCE [78,79]; NCT03214250	Metastatic; 1L	Sotigalimab and/or nivolumab with chemotherapy	105 efficacy; includes 12 phase 1b	T2	Only nivolumab arm met historical OS benchmark	1-year OS 57.7% in nivolumab arm	14 paired tumors; exploratory correlates	a; b; c; d
OPTIMIZE-1 [80,119]; NCT04888312	Metastatic; 1L	Mitazalimab + mFOLFIRINOX	70 treated; 65 RP2D; 57 efficacy	T3	Response criterion: yes	ORR 24/57; all-RP2D sensitivity 24/65	Three paired biopsies; all responders	a; b; c; d; e
Radiotherapy- and local ablation-based combinations								
CheckPAC [93]; NCT02866383	Metastatic; refractory	SBRT/nivolumab ± ipilimumab	88 randomized; 84 treated	T2	Dual-checkpoint arm met CBR screen	Six versus one partial response; OS 3.8 months both	Baseline/on-treatment tumor IHC; pairing not established; serial blood	b; d
Vaccine and antigen-directed combinations								
KG4/2015 [85,85]; NCT02854072	Advanced; 1L	GemCap ± GV1001	111 randomized high-eotaxin; 37 nonrandom controls	T1/T3	Reported pooled OS: positive	Pooled OS 11.3 versus 7.5 months	Blood/skin vaccine responses; high-stratum comparative result not located	c; e
Autogene cevumeran [83,139]; NCT04161755	Resected; adjuvant	Atezolizumab/vaccine/mFOLFIRINOX	16 vaccinated; 15 chemotherapy	T3	Safety primary; immune response exploratory	8/16 immune responders; RFS association	Blood and selected lesions; landmark analysis performed	a; d
PARP-inhibitor and maintenance combinations								
Niraparib combinations [152,152]; NCT03404960	Platinum-controlled maintenance	Niraparib + nivolumab or ipilimumab	91 randomized; 84 efficacy	T2	Ipilimumab PP benchmark met	ITT interval did not exclude null benchmark	No verified on-treatment tumor immune result	a; b; d; g
POLAR [153]; NCT04666740	Maintenance; genomic cohorts	Olaparib + pembrolizumab	63 treated; core HRD 33	T3	Co-primary criteria: no	Core-HRD ORR 7/20; 6-month PFS 64%	Baseline tissue and serial ctDNA; assay/criterion discrepancies	a; b; e; g
SWOG S2001 [154]; NCT04548752	gBRCA1/2 maintenance	Olaparib ± pembrolizumab	73 enrolled; 68 eligible	T1	PFS: futility stop	PFS 8.2 versus 6.4 months; *p* = 0.82	Translational results not reported in conference abstract	f

Table 2 notes: Comparisons are within trials. Enrolled, treated and evaluable populations are labeled; response and survival results from different studies are not ranked. Tiers and descriptive flags are defined in Section 2.4. Additional denominators, source-access limits and correlative details appear in Table S5.

## 6. Mechanistic Barrier Map: Matching Combination Components to Immune Resistance in PDAC

Combination design should specify the proposed contribution of each component, how it might interact with other components, and the clinical comparison needed to test for benefits. The four groups provide a checklist for these hypotheses [5,26].

### 6.1. Barrier 1: Insufficient Antigenicity, Presentation, and Priming

For antigen-supplying or priming strategies, informative endpoints include antigen-specific responses and recognition of naturally processed tumor antigens. Delivery or replication is relevant to oncolytic viruses; altered presentation or innate sensing may be relevant to cytotoxic conditioning [29,30,31]. The assay should test the claimed component function and report its evaluable denominator.

Response frequency, clonal breadth, persistence and tumor recognition provide complementary information. A small melanoma study associated durable checkpoint responses with persistent, polyclonal neoantigen-specific T cells [158]. Experimental studies show that peptide-reactive cells need not recognize tumor cells and that antigen hierarchy or persistent vaccine depots can limit activity [159,160,161,162,163]. These findings support functional validation where feasible; they do not establish a universal PDAC surrogate endpoint.

### 6.2. Barrier 2: T-Cell Exclusion by Matrix, Vasculature, and Chemokine Programs

For a strategy intended to reverse exclusion, spatial tissue analysis can test entry into malignant glands rather than accumulation in blood or stroma. Where repeat biopsy is safe and informative, paired samples strengthen this inference. Bulk CD8 counts alone cannot resolve location or tumor specificity [44].

Candidate measurements include CAF state and location, tumor-reactive T-cell proximity, and compensatory myeloid changes [40,44,61,164,165,166]. Their interpretation is context-specific: selective LRRC15-positive CAF depletion improved checkpoint activity in mice, whereas other stromal deletions accelerated disease [36,40,164]. These observations motivate prospective pharmacodynamic studies; they do not define a validated universal test separating beneficial remodeling from harmful remodeling.

### 6.3. Barrier 3: Suppressive Myeloid and Regulatory-Cell Programs

For myeloid-directed treatment, specify whether the aim is altered recruitment, depletion or functional reprogramming. Cell state, suppressive activity and effector-cell function can distinguish these effects; target occupancy or peripheral cell counts alone cannot establish them. CD40 agonism also supports priming, illustrating overlapping assignments [49,167,168].

Compensation warrants measurement: human CCR2 inhibition was associated with increased tumor neutrophils [49]. In mouse PDAC, durable activity was achieved with 4-1BB agonism plus LAG-3 and CXCR1/2 inhibition [67], while a separate study implicated several inhibitory checkpoints in resistance to chemoimmunotherapy [68]. These are distinct experimental regimens, not proof that every myeloid strategy requires multi-checkpoint blockade.

### 6.4. Barrier 4: T-Cell Dysfunction and Exhaustion

PD-1/PD-L1 blockade can support tumor-reactive T-cell function, while CTLA-4 interventions can affect priming and regulatory cells as well as effector responses [64]. Negative checkpoint trials establish limited activity in the studied populations; they do not identify which upstream mechanism failed [8,137]. Functional immune measurements and regimen-specific toxicity should inform further combinations.

### 6.5. Integrating Barriers, Sequence, and Pharmacodynamic Proof

Sequence and concurrent dosing should be tested as pharmacologic hypotheses, considering exposure, immune kinetics and cumulative toxicity. A lead-in can isolate a short-term biological change, but the appropriate design depends on the question and does not by itself establish clinical contribution.

Interpret results separately at target engagement, downstream immune change and clinical benefit. Demonstrating one does not establish the next. Tissue accessibility, missing samples, assay performance and alternative explanations should limit the strength of a mechanistic conclusion.

A dominant-barrier claim would require evidence that modifying the proposed limitation changes the relevant immune response more than competing explanations predict. The present scheme supplies no common scale for ranking its four groups. Trials can instead prespecify a narrower mechanistic hypothesis, an appropriate assay and sampling subset, and the uncertainty that remains when sampling is incomplete. These are research recommendations, not universal biopsy eligibility or advancement requirements.

**Figure 2:**
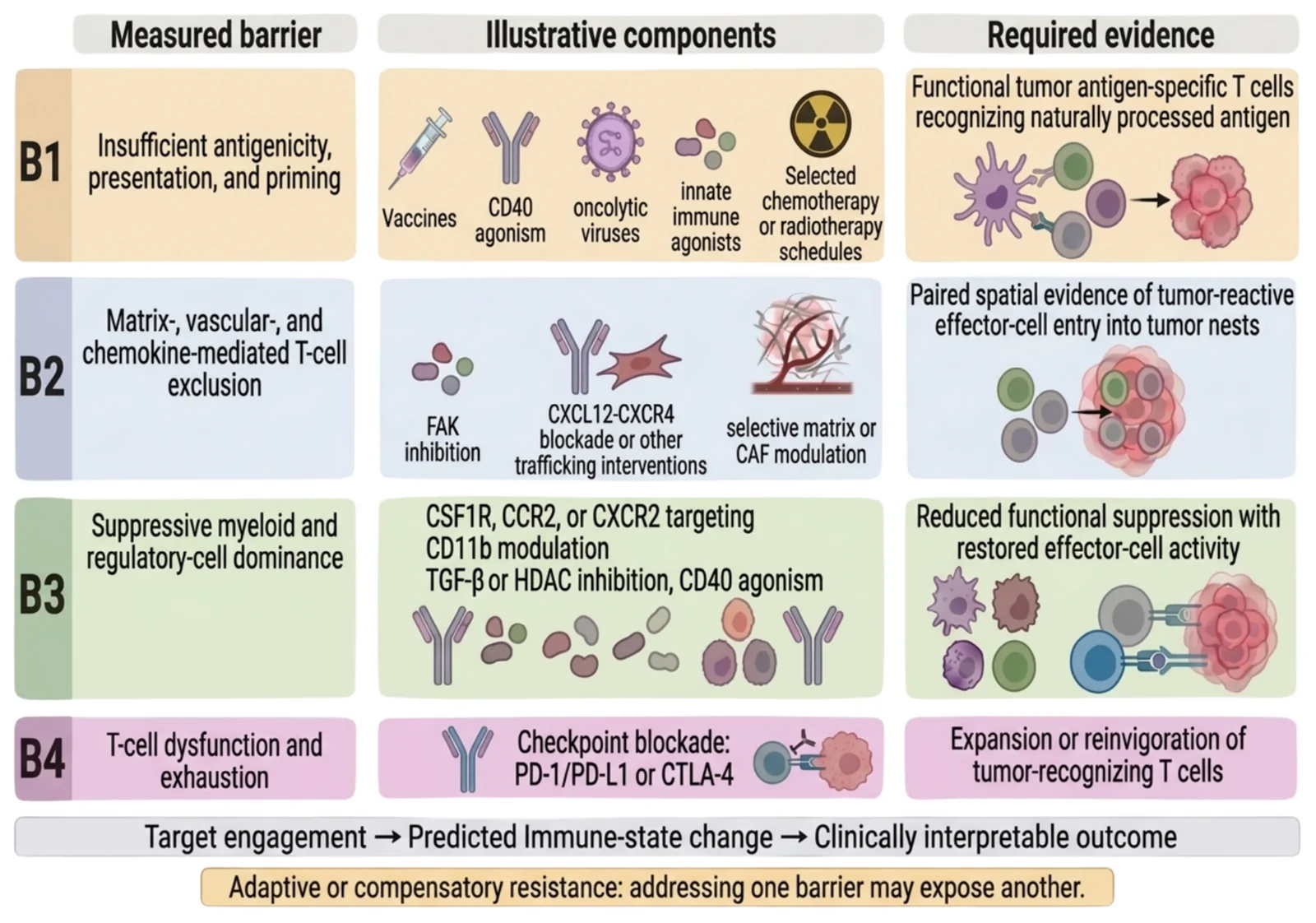
Component functions and proposed evidence within the four-barrier scheme. “Required evidence” denotes substantiation of the specified mechanism claim, not universal assay, biopsy eligibility or clinical advancement requirements. Components and mechanisms overlap; CTLA-4 can affect priming and regulatory cells, and CD40 spans priming and myeloid programs. Assay selection and tissue sampling depend on the question, safety and feasibility. The scheme is not a validated clinical treatment-selection model.

This barrier-matched combination logic is summarized in Figure 2.

## 7. Biomarkers and Patient Selection

Biomarkers can guide treatment selection, enrich a trial, describe prognosis or measure a biological response. Regulatory authorization and evidence for treatment-effect modification should be reported separately. dMMR/MSI-H and TMB-high support tumor-agnostic treatment selection under their applicable conditions; neither identifies the best PDAC combination. Exploratory associations require assay validation and independent evaluation for their intended use.

### 7.1. Clinical Selection Now

#### 7.1.1. dMMR/MSI-H

dMMR/MSI-H is an established selection biomarker for tumor-agnostic pembrolizumab, although PDAC-specific response estimates remain imprecise. It does not establish which additional immune or targeted component should be combined with the PD-1 blockade. One series found dMMR in 7 of 833 PDACs (0.8%) [169]. Prevalence varies with histology and assay, with enrichment in uncommon medullary and mucinous/colloid tumors; conventional morphology does not exclude the biomarker [170].

KEYNOTE-158 included 22 patients with previously treated MSI-H/dMMR pancreatic cancer. Four had confirmed responses (18.2%; 95% CI, 5.2–40.3), with median PFS and OS of 2.1 and 4.0 months [9]. Retrospective series report higher response proportions, but differences in referral, treatment, assay and outcome availability prevent direct comparison [171,172]. These findings support access to an established molecularly selected treatment while recognizing that benefit is not universal.

The current US indication covers adult and pediatric patients with unresectable or metastatic MSI-H/dMMR solid tumors, determined by an FDA-authorized test, after progression on prior treatment when no satisfactory alternative exists [173,174]. FDA-authorized routes include the VENTANA MMR RxDx immunohistochemistry panel for dMMR solid tumors and sequencing assays with the relevant indication [175,176]. The label recommends confirmation of a local MSI-H/dMMR result using an FDA-authorized test when feasible; it does not require routine duplicate testing after every authorized result [174]. Discordance can occur: the Mayo series reported discrepant results in 19% of its selected cohort and 26% of cases evaluable by at least two methods [177]. A responding MMR-deficient/MSS case also had other potentially relevant molecular features [178]. These observations support specimen review and orthogonal adjudication rather than a universal testing sequence.

A practical workflow starts with the available specimen and treatment decision. Select an appropriate validated tissue assay or panel covering the relevant biomarkers, then review tumor content and assay limitations. IHC, PCR and NGS provide complementary information; discordant findings warrant pathological and molecular review with complementary testing where feasible. Sequencing can assess other actionable alterations and TMB within the available tissue. Plasma testing may help when tissue is limited, but a negative result with low tumor shedding does not exclude an alteration. Apply the indication’s treatment and authorized-assay conditions when selecting therapy. This is a specimen-aware workflow, not a mandatory order of IHC, PCR and NGS (Section 7.4) [169,170,172,173,174,175,176,177,178].

#### 7.1.2. Tumor Mutational Burden-High

TMB-high status provides another tumor-agnostic pembrolizumab indication under specified prior-treatment and assay conditions, using a threshold of at least 10 mutations per megabase. Accelerated approval rested on response rate and durability; the KEYNOTE-158 TMB-high efficacy cohort did not include PDAC [174,179,180]. TMB-high PDAC is uncommon and overlaps partly with dMMR/MSI-H, while some microsatellite-stable tumors also have high TMB [181,182]. In a retrospective series, 10 TMB-high and 41 TMB-low checkpoint-treated patients had median OS of 25.7 and 5.2 months, respectively (HR 0.32; 95% CI, 0.11–0.91) [183]. This association supports clinical relevance but does not establish a PDAC-specific treatment interaction; small groups, residual confounding and overlapping molecular features limit inference. TMB also does not measure T-cell access or function.

### 7.2. Trial-Enrichment Markers Under Validation

#### 7.2.1. The PA.7 Concurrent DNA Damage–Repair Alteration Signature

HRD is not a validated predictor of added checkpoint benefit in PDAC. Cohort studies have reported differing relationships between HRD, mutation burden and intratumoral immunity [184,185]. PA.7’s exploratory four-gene rule is a candidate biomarker requiring independent validation [110]. Nineteen genes were selected because mutations occurred in immunotherapy-arm patients surviving over three years; arm-specific Lasso models and subsequent biological selection identified BRCA1, POLE, ATM and FANCA. Somatic and germline-likely status were counted separately for each gene, yielding eight possible “hits”; ≥2 hits does not necessarily mean mutations in two different genes. Of 173 patients, 18 were positive, including 11 in the immunotherapy arm, one of whom was MSI-high. Within that arm, OS was 26.16 versus 9.72 months (HR 0.34, 95% CI 0.16–0.68), with treatment-interaction *p* = 0.003. The same cohort derived and evaluated the rule, leaving multiplicity and model-selection uncertainty unresolved.

Archival whole-genome sequencing was available in 46 patients. Twelve of fifteen germline-likely and two of seven somatic plasma variants had matched tissues; five of six plasma-positive cases were tissue-positive, with three additional tissue-only positives [110]. Sampling intervals varied widely. This small, partially concordant technical comparison is not an independent predictive validation. The variant set included variants of uncertain significance and neutral annotations and should not be equated with pathogenic DDR mutations or functional HRD. Clinical application requires a locked assay and rule, as well as independent evidence of treatment interaction; it should not be generalized to any BRCA mutation, PARP sensitivity or checkpoint monotherapy.

POLAR cannot distinguish platinum-sensitive biology and olaparib activity from pembrolizumab contribution, while preliminary SWOG S2001 results did not demonstrate benefit from adding pembrolizumab in germline BRCA1/2-mutated maintenance disease [153,154]. Neither result validates generic HRD as a predictor of checkpoint-combination benefit.

#### 7.2.2. Barrier-State Enrichment

Spatial T-cell localization, tertiary lymphoid structures, CAF programs and myeloid states are candidate research markers [186,187]. PRINCE immune signatures and CheckPAC circulating-protein analyses remain exploratory [79,188]. CheckPAC did not associate PD-L1 staining at a 1% threshold with clinical benefit [93]; this assay should not be generalized as a PDAC combination-selection test.

B-cell and lymphoid-structure associations with checkpoint response in other cancers do not establish predictive use in PDAC [189]. PDAC spatial and lymphoid findings are principally prognostic or exploratory [166,190]. Trials should prespecify the compartment, assay and treatment contrast, distinguishing cell state and location from abundance.

### 7.3. Exploratory Correlatives

Microbiome features and single-cell or spatial signatures can generate testable hypotheses but are not established PDAC treatment selectors [191,192,193,194]. Contamination, antibiotic exposure, tissue handling and analytic choices require control. Experimental perturbations can strengthen mechanistic inference while leaving human therapeutic benefit unresolved.

### 7.4. Standardization, Specimen Adequacy and Trial Use

Biomarker protocols should define the specimen, timing, assay, quality criteria, threshold, analysis population and intended role. Baseline tissue can support selection, while on-treatment tissue can assess target engagement and spatial change [95,101,131]. Serial blood assays can measure circulating tumor markers [195] or immune responses. Blood immunogenicity may be appropriate after complete resection, when no residual tumor is available for biopsy [83,84,139]. These endpoints answer different questions. Report biomarker-negative and unevaluable patients separately and distinguish the clinical population from the paired-sample subset.

Specimen feasibility should be established for the intended assay. The ESGE technical and technology review recommends end-cutting fine-needle biopsy (FNB) for solid pancreatic lesions, with technique and processing adapted to the clinical setting [196]. In a randomized study of 370 patients, including 234 with pancreatic lesions, FNB with macroscopic on-site evaluation was noninferior to conventional three-pass sampling for diagnostic accuracy [197]. Diagnostic adequacy does not establish that paired samples will support sequencing, multiplex immunophenotyping or spatial analysis. These research assays need their own quality criteria and acquisition plans.

Molecular-testing yield depends on the whole care pathway. In Know Your Tumor, 1082 of 1856 referred patients received a molecular report; deaths, health concerns, loss to follow-up and other barriers contributed to attrition [198]. High adequacy among specimens already submitted for testing does not establish the cause of earlier losses [199]. One retrospective EUS series found sequencing adequacy in 70.1% of the samples, with higher adequacy for FNB than FNA, but results vary with specimen processing and assay requirements [200,201]. COMPASS obtained successful whole-genome sequencing in 62 of 63 biopsied patients with pancreatic cancer, including 61 with PDAC, using a selected percutaneous-biopsy workflow and tumor enrichment [202]. These studies do not establish a universal one-third loss rate for paired biopsies. Report collection and assay success at each time point, with reasons for missing samples. ctDNA detection also depends on disease stage and assay: negative plasma testing is especially limited in localized disease and cannot substitute for spatial tumor measurements [203,204,205].

Table 3 places the retained markers in this hierarchy.

## 8. Challenges, Limitations, and Safety Considerations

Safety interpretation requires the event category, attribution rule, population and exposure period. A grade 3 event is not synonymous with a serious adverse event, and investigator-attributed toxicity is not identical to all treatment-emergent events. Concurrent comparisons can estimate incremental burden for the components actually differing between arms.

### 8.1. Regimen-Specific Toxicity

Table S6 reports regimen-specific safety with its original denominators and attribution categories. PA.7 reported grade ≥ 3 adverse events in 100 of 119 combination-treated and 44 of 58 chemotherapy-treated patients; two grade 5 events were considered at least possibly immunotherapy-related [76]. Two grade 5 events in the chemotherapy arm were also considered at least possibly treatment-related. Such within-trial estimates are more informative than toxicity ranges pooled across different populations and reporting rules.

Dose-finding studies can identify serious hazards even when they cannot estimate incremental efficacy. Ipilimumab plus gemcitabine reported grade ≥ 3 treatment-related adverse events in 16 of 21 patients [118]. A tremelimumab–gemcitabine study reported a fatal treatment-related diarrhea/dehydration event [117]. For defactinib, the published correction attributes a heart-failure event to causes unrelated to the study combination [95]. Attribution must follow the corrected report.

POLAR consistently reported no grade 4 or 5 treatment-related adverse events [153]. Its grade 3 reporting nevertheless requires clarification: anemia appears as 10 patients in the Results section and its table caption but 13 in Table 3 of the POLAR report, and colitis is described among grade 3 events in the narrative but appears as grade 2 in the table. These discrepancies limit precise event-specific estimates; they do not make all reported safety outcomes uninterpretable. The discordant grade 3 counts and response-criterion descriptions warrant correction or author clarification; unaffected safety findings remain interpretable.

Maintenance treatment may reduce cytotoxic exposure while introducing immune toxicity. Niraparib–ipilimumab had more grade 3–4 treatment-related events than niraparib–nivolumab in the reported safety populations [152]. GVAX–ipilimumab maintenance collected adverse events only in the experimental arm, preventing a balanced toxicity comparison with continued chemotherapy [77]. Benefit and burden require assessment within the same randomized clinical question. Table 4 summarizes regimen-specific safety findings and the limitations of their interpretation.

**Table 4:** Regimen-specific safety of combination immunotherapy in PDAC, summarized by regimen class.

Regimen Context	Event Category	Exposure/Population	Informative Comparison	Reported Finding or Interpretation
Checkpoint plus chemotherapy	Grade ≥ 3 AEs; possible immune attribution	PA.7: 119/58 treated	Same chemotherapy backbone [76]	100/119 versus 44/58; two possible immune-related grade 5 events in the combination arm and two possible treatment-related grade 5 events in controls.
CD40 plus chemotherapy	Study-specific treatment-related events	Dose-finding and selected efficacy cohorts	PRINCE lacks chemotherapy alone [78,79,80]	Whole-regimen and CD40-attributed events must remain separate.
Radio-immunotherapy	Regimen-related events	Different sites, fractionation and treatment lines	CheckPAC varies ipilimumab, not radiation [93]	Randomization can inform the added checkpoint contrast; it cannot isolate radiation toxicity.
FAK/trafficking combinations	Investigator attribution; corrected where published	Small early-phase cohorts	Generally limited comparative precision [95,131]	Defactinib notice removes erroneous heart-failure attribution.
Myeloid combinations	Study-specific categories	Treated populations; variable attrition	BMS and other concurrent comparisons [103,104,135]	Use the safety population and changed components; do not rank pooled class rates.
Vaccine combinations	Vaccine versus complete-regimen attribution	Perioperative and advanced settings	GVAX–ipilimumab collected experimental-arm AEs only [77]	Comparative tolerability cannot be estimated from one-arm collection.
Dual checkpoint blockade	Grade ≥ 3 treatment-related AEs	64 treated in durvalumab ± tremelimumab study	32 combination versus 32 monotherapy [8]	7/32 versus 2/32; precision is limited.
PARP/checkpoint maintenance	Treatment-related events	Selected nonprogressing patients	Niraparib ICI-partner assignment [152]; uncontrolled POLAR [153]	POLAR grade 3 anemia/colitis details conflict; no grade 4–5 treatment-related events reported.
Perioperative checkpoint combination	Grade ≥ 3 events at least possibly related	37 randomized and treated	Pembrolizumab addition [127]	9/24 versus 4/13; small numbers do not establish added harm.

Table 4 notes: Rates retain the source’s event and attribution category. Differing denominators, exposure and disease settings prevent comparison between rows. No event rate should be interpreted as the toxicity of an individual component unless the design and attribution support it.

### 8.2. Feasibility in the Perioperative Setting

Perioperative studies must report all enrolled patients alongside those reaching treatment, exploration and resection. Progression, toxicity, logistics and surgical selection can each prevent resection; an uncontrolled nonresection rate does not establish treatment-related harm. Operative morbidity, treatment delays and postoperative recovery are distinct outcomes. In the small randomized neoadjuvant pembrolizumab study, grade ≥ 3 events at least possibly related to therapy occurred in 9 of 24 versus 4 of 13 patients, and 17 versus 7 underwent pancreatectomy [127]. These estimates describe feasibility with substantial uncertainty, rather than proving added harm or benefit. Tissue denominators differed by assay and should not be substituted for the randomized populations.

Single-arm neoadjuvant and vaccine studies often restrict biological analyses to adequate resection specimens [115,121,144,206]. Selected resected-patient survival can be clinically informative but cannot establish the effect of the neoadjuvant regimen. Trials should prospectively report surgical timing, reasons for nonresection, and morbidity, with a contemporary comparator when clinical benefit is the question.

Personalized vaccines introduce manufacturing and timing constraints in addition to toxicity concerns [83,139]; shared-antigen peptide vaccines avoid individualized manufacturing but retain the timing constraint [84]. Future adjuvant studies should report manufacturing failures, time from surgery to first dose, missed chemotherapy, and recurrence before dosing as feasibility outcomes.

### 8.3. Quality of Life and Treatment Burden

Quality of life should accompany efficacy and safety assessment. In the metastatic chemotherapy trial, FOLFIRINOX delayed definitive QoL deterioration compared with gemcitabine despite greater toxicity [2]. For immunotherapy combinations, PA.7 found no significant increase in deterioration at 8 and 16 weeks of physical function or global health on QLQ-C30, although missing assessments limit interpretation [76,207]. In uncontrolled CA209-9KH, global health declined alongside progression, preventing attribution to treatment alone [129]. An unreported QoL decline is not evidence that a regimen has no treatment burden.

QLQ-C30 assesses pain, appetite, constipation and diarrhea; PAN26 adds pancreatic-specific pain, digestive symptoms, bowel urgency and jaundice-related problems [207,208]. PAN26 meaningful-change thresholds derive from resected patients receiving adjuvant chemotherapy, so instrument choice must reflect the setting [209,210]. Where validated, report PAN26 alongside QLQ-C30 at baseline, fixed follow-up intervals, discontinuation or progression, and feasible post-treatment follow-up. Prespecify meaningful deterioration, confirmation rules, and handling of death and missing questionnaires. A patient-reported primary or co-primary endpoint merits consideration when reduced burden is central, particularly in maintenance studies.

Relevant outcomes include global health, physical and role function, pain, fatigue, appetite, pancreatic symptoms, steroid exposure and treatment discontinuation. Perioperative and maintenance studies should also assess return to usual activity, nutritional and endocrine consequences, and time off cytotoxic therapy. These are proposed priorities for a trial design, not evidence that a particular combination improves them.

### 8.4. Design Limitations That Affect Safety and Efficacy Interpretation

Small, open-label studies often combine heterogeneous treatment histories, post-treatment selection and limited sampling. A shared immune agent cannot have its own contribution estimated by randomizing only the other component. Conversely, randomization between experimental regimens can still answer the specific component contrast that differs. External controls and historical benchmarks require assumptions about comparability; dose-finding participants should be identified separately when included in later efficacy analyses [15,109]. The descriptive flags in Table S5 identify the affected analysis without converting every limitation into a study-wide judgment.

Endpoints should match the clinical setting. Response rates can screen metastatic regimens, but clinical-benefit rates depend on the required duration of stable disease. Resection and R0 rates require explicit surgical denominators, and recurrence comparisons between immune responders and nonresponders require a justified landmark and recognition of residual confounding. Reports should distinguish the prespecified response criterion, the criterion actually implemented, and the analysis population. POLAR’s iRECIST/RECIST 1.1 discrepancy illustrates why these details matter [153].

Sampling can select patients who remain sufficiently well for an on-treatment biopsy or who have analyzable tissue. Studies should report collection, assay success and missingness by time point, while retaining the appropriate clinical analysis population. Missing tissue limits mechanistic inference; it does not prove target failure. Advancement decisions should integrate clinical signal, feasibility, biological evidence and treatment burden rather than require one universal biopsy rule.

Table 5 distinguishes human biological observations from clinical evidence and readiness.

Table 6 compares the focus and scope of six selected reviews. This purposive comparison identifies complementary contributions rather than establishing that no previous review considered a particular mechanism or trial limitation.

**Table 6:** Scope and contribution of selected reviews of PDAC immunotherapy.

Review	Article Type	Principal Focus	Clinical Scope	Mechanistic Contribution	Review Basis	Complement to This Review
Hilmi 2023 [13]	Narrative	Immune landscape and resistance	Novel combinations and biomarkers	Heterogeneity and stromal crosstalk	Stated scope; full text limited	Individualized strategies
Ju 2024 [12]	Narrative	Barriers and opportunities	Therapeutic strategies	TME, mouse models and intrinsic pathways	Stated scope in primary abstract	Model-specific mechanism context
Farhangnia 2024 [14]	Narrative	Current/future immunotherapies	Broad modality coverage	Suppressive TME and platforms	Stated scope in primary abstract	Breadth of therapeutic classes
Shakiba/Tuveson 2025 [11]	Narrative	Macrophages and fibroblasts	Mechanism-led implications	CAF/TAM heterogeneity and spatial organization	Stated scope; full text limited	Detailed stromal/myeloid biology
Al-Khinji 2025 [15]	Systematic review/meta-analysis	Checkpoint clinical outcomes	Studies through February 2024	Clinical synthesis with bias assessment	Methods stated in primary abstract	Broader outcome synthesis
Qi 2026 [16]	Systematic review/meta-analysis	First-line ICI combinations	Advanced disease; search through April 2026	RCT and single-arm analyses separated	Methods stated in primary abstract	Focused first-line estimates
Present review	Narrative	Component function and tested contrast	Nonexhaustive study/cohort inventory	Overlapping four-group heuristic	Study design and assay appraisal	Links clinical inference to actual biological measurements

Table 6 notes: Scope statements derive from the reviews’ stated aims and methods. Full-text access is identified where relevant; unavailable details are not coded as absence. Publication year alone does not establish coverage of a later trial report.

## 9. Future Directions

The priorities below distinguish changes feasible in current trials from early clinical signals and more exploratory platforms. The organizing question is whether a defined component improves a relevant outcome with acceptable burden.

### 9.1. Priority 1: Trial-Design Improvements That Can Be Implemented Now (B1–B4)

Trials can specify the biological role of each component and choose a comparator that tests its contribution. Baseline stratification should use an assay with a defined context of use; an unvalidated barrier score should not become an automatic eligibility gate. Pharmacodynamic endpoints and sampling subsets should match the intended claim and disease setting.

Dose, sequence or an added third component may be tested through randomized or factorial comparisons when feasible. A short lead-in can examine an early biological change, with clinical contribution tested separately. Adaptive enrichment requires prespecified biomarker rules, operating characteristics and adaptation criteria [211].

Sampling feasibility should inform the design: estimate acquisition and assay yield, record missing specimens, and justify the immune-endpoint sample size. Standardized processing, assay validation and central analysis may improve comparability. Blood-based immunogenicity or molecular-residual-disease endpoints can be appropriate when repeated tumor tissue is unavailable, provided their limitations are explicit. Sample-size planning should incorporate marker prevalence and expected assay yield; small positive subgroups require prespecified interaction analyses and uncertainty estimates.

### 9.2. Priority 2: Early Human Signals Requiring Controlled Validation (Principally B1)

Personalized and shared-antigen vaccines warrant controlled testing in resected PDAC because early studies demonstrate immunogenicity and persistent vaccine-expanded T cells [83,84,139,212]. Trials should test recurrence, manufacturing feasibility and access, and directly determine the contribution of checkpoint blockade. ELI-002 also produced persistent KRAS-specific responses after standard treatment, but its phase 1 cohort comprised 20 patients with PDAC and 5 with colorectal cancer, and it tested vaccine monotherapy [213]. The strongest reported immune–survival separation used an outcome-optimized response threshold in this mixed cohort; subsequent therapy and censoring further limit interpretation. It supports the platform’s immunogenicity, not the efficacy of an immunotherapy combination.

Trafficking, myeloid and stromal combinations should test the proposed biological change in an informative subset while preserving a clinical comparison [95,98,101]. A measured change can guide further investigation without being treated as a validated surrogate for benefit.

The PA.7 four-gene status-hit rule warrants independent replication with a locked assay and prespecified interaction analysis [110]. Maintenance studies should separate PARP-inhibitor activity from the added immune component and assess time off chemotherapy alongside toxicity and disease control [152,153,154].

Shared-control platforms can compare several strategies within a common population, but allocation, concurrent controls and changing standards require careful design. MORPHEUS demonstrates feasibility and the need to distinguish shared controls from new participants [100]. Such platforms do not by themselves validate cross-arm mechanistic rankings. Comparative claims still require adequate allocation and power for the relevant contrast.

### 9.3. Priority 3: Early Clinical Platforms Needing Solutions for Delivery and Resistance (Principally B1 and B2, with B3 and B4 as Resistance Constraints)

Oncolytic and innate platforms need evidence of delivery and a tumor-directed immune response to substantiate those particular mechanism claims, with antiviral responses distinguished from antitumor activity [88,89]. Clinical efficacy requires controlled confirmation.

Cellular and T cell-redirecting strategies face antigen heterogeneity, trafficking, persistence and normal-tissue toxicity [73,74,75]. TACTOPS supplies early combination context in a selected cohort continuing standard treatment; small engineered-cell studies provide separate platform evidence. Additional agents should address a defined, testable limitation rather than simply increase component count.

### 9.4. Priority 4: Enabling Technologies and Exploratory Modifiers (Measurement of B1–B4)

Single-cell, spatial and serial blood technologies can define heterogeneity and treatment-associated change [24,25,61,195]. Their utility depends on assay performance and whether the result informs a prespecified decision. Molecular response, immune activation and spatial redistribution remain different endpoints.

Microbiome studies provide human associations and experimental mechanisms, but no established PDAC therapeutic benefit [191,192,193]. MITRIC tested fecal transfer with continued checkpoint therapy in a mixed-cancer cohort that included one MSI-H PDAC patient, who progressed [214]. This small clinical observation prevents calling the approach wholly untested while providing no basis for efficacy claims.

Priorities should remain responsive to controlled clinical results, reproducible biological findings and patient burden.

## 10. Conclusions

Combination immunotherapy remains investigational for most patients with PDAC. Negative randomized survival studies constrain routine checkpoint addition to chemotherapy, while early response and immunogenicity signals justify focused testing rather than broad adoption. Positive findings require attention to the actual contrast: NASCA changed several components, KG4/2015 pooled randomized and nonrandomized controls, and early vaccine benefits were not consistently confirmed. Tumor-agnostic treatment selection in dMMR/MSI-H or TMB-high disease remains distinct from evidence that adding another component improves PDAC outcomes. The four-barrier heuristic organizes overlapping mechanisms and the measurements needed to test a proposed component function. It does not establish a universal causal hierarchy or a validated clinical algorithm. Further progress depends on appropriate comparators, independent biomarker validation, setting-specific sampling and patient-reported outcomes. Clinical benefit, target engagement and downstream immune change should be appraised separately, with conclusions limited to what each study measured. Priorities include resected vaccine cohorts, controlled component tests in platinum-sensitive germline BRCA1/2 maintenance disease, and independently validated molecular or immune research subsets.

## Figures and Tables

**Table 3:** Clinical and investigational biomarkers for combination immunotherapy in PDAC.

Biomarker or State	Evidence Status	Assay or Definition	Current Role	Principal Limitation	Barrier Link
dMMR/MSI-H	Established tumor-agnostic selection [9,169]	Validated IHC, PCR or NGS; authorized assay for indication	Pembrolizumab under label conditions [173,174,175]	Rare; discordance requires review; added-combination benefit unknown	B1
TMB-high	Tumor-agnostic selection [179,180,181]	≥10 mutations/Mb under assay-specific conditions	Label-based treatment selection	Limited PDAC evidence; molecular overlap and assay dependence	B1
PA.7 four-gene status-hit rule	Exploratory treatment-interaction signal [110]	≥2 somatic/germline-likely status hits across BRCA1, POLE, ATM, FANCA	Independent validation research	Same-cohort derivation; 18 positive; not equivalent to functional HRD	B1 hypothesis
Core HRD	Maintenance enrichment; added ICI benefit unvalidated [153,154]	BRCA1/2 or PALB2-defined cohorts; study-specific rules	PARP/immune combination trials	Platinum selection; POLAR uncontrolled; S2001 preliminary negative	B1 hypothesis
T-cell/spatial state	Exploratory [166,186,187,189,190]	Compartment-specific IHC, RNA or spatial assay	Research stratification and pharmacodynamics	Prognostic association is not treatment prediction	B1/B2/B4
CAF/stromal state	Exploratory [61,164,165]	Cell state, location and functional context	Mechanistic research	Heterogeneous tumor-promoting and restraining functions	B2/B3
Myeloid state	Exploratory [49,79,103]	Phenotype, location and functional assays	Pharmacodynamic research	Abundance alone does not establish suppression	B3
Microbiome features	Exploratory [191,192,193]	Controlled specimen and sequencing workflow	Hypothesis generation	Contamination, confounding and unestablished clinical utility	Several

Table 3 notes: Biomarker roles are context-specific. A tumor-agnostic indication does not establish the added benefit of a PDAC combination. Regulatory conditions, assay limitations and exploratory analyses are detailed in Section 7.

**Table 5:** Biological evidence, comparative clinical findings and readiness by regimen class.

Regimen Class	Target Engagement or Proximal Effects	Downstream Tumor Immune Change	Comparative Clinical Finding	Design Context	Clinical Status
Checkpoint plus chemotherapy	Immune assays vary	Limited subsets; not universal target-occupancy data	PA.7 and CISPD3 missed primary OS; NASCA changed several components [76,111,112]	T1–T3	Investigational
CD40 plus chemotherapy	Peripheral and tissue pharmacodynamics	Selected paired or resection specimens	Uncontrolled OPTIMIZE signal; PRINCE tests historical arm benchmarks [79,119,121]	T2/T3	Investigational
Radio-immunotherapy	Some out-of-field responses and tissue effects	Sampling differs by trial	CheckPAC tested each arm separately and did not establish comparative survival benefit [93]	T2/T3	Investigational
Stromal/trafficking combinations	Human spatial and pathway changes	Tissue observations in COMBAT; paired subsets in plerixafor and defactinib	No established routine benefit; experimental FAK biology is not identical to kinase inhibition [70,95,98,131]	T2/T3	Investigational
Myeloid combinations	Recruitment and pathway changes	Cell density does not establish function	Galunisertib result was sensitive to external borrowing; other controlled studies mixed/negative [103,104]	T1–T3	Investigational
Vaccines with checkpoint blockade	Antigen-specific responses; selected clonotypes	Some paired studies and selected lesions	GVAX–ipilimumab maintenance was inferior; nivolumab addition did not improve OS; other comparisons exploratory [77,140,141,142,145]	T1–T3	Investigational
Dual checkpoint blockade	Limited clinical immune inference	Trial-dependent	Limited unselected activity; more agents do not ensure benefit [8,137]	T2/T3	Investigational
PARP/checkpoint maintenance	Exploratory molecular correlates	Baseline and serial blood; limited tumor dynamics	POLAR endpoints unmet; S2001 preliminary negative; niraparib signal depends on analysis set [152,153,154]	T1–T3	Investigational
dMMR/MSI-H PD-1 monotherapy	Established molecular selection	Diagnostic assay, not a combination mechanism test	Tumor-agnostic authorization; small PDAC response cohort [9,174]	Separate single-agent context	Established under applicable label

Table 5 notes: Human pharmacodynamic observations do not necessarily involve direct target engagement. Clinical findings refer to the tested regimen and comparator, not an entire class. All listed combination classes remain investigational for routine PDAC treatment; biomarker-defined tumor-agnostic monotherapy indications are addressed separately in Section 7.

## Data Availability

No new primary or participant-level data were generated. All data analyzed in this review were extracted from the cited publications and, where indicated, from the corresponding trial registry records. The review does produce one derived dataset: the coding matrix assigning an evidence tier, per-study bias flags, a target-barrier assignment and a barrier-measurement status to each of the 84 studies, which is reported in full in Supplementary Table S5. The complete search strings, the study-flow counts and the exclusion breakdown are provided in the Supplementary Material so that the search can be re-executed; one unresolved element of the retrieval-stream record is declared in Supplementary Section S3.3.

## Supplementary Materials

The following supporting information can be downloaded at: https://www.mdpi.com/article/10.3390/cancers18183019/s1. Sections S1–S3 retain the historical search strings and reported flow with explicit provenance limitations; Section S4 describes reference verification; Sections S5–S6 define coding and source-access conventions; Table S5 retains 84 of the 85 supplied study/cohort entries; Table S6 gives report-specific safety data; and Section S9 provides extended notes. The supplementary reference list contains 95 index, linked, and notice entries. Shared publications have separate main and supplementary citation numbers; additional inventory references are included here [215,216,217,218,219,113,220,221,222,94,223,224,225,226,227,228,229].

## Abbreviations

The following abbreviations are used in this manuscript and its Supplementary Material:

ASCO	American Society of Clinical Oncology
*ATM*	ataxia-telangiectasia mutated
B1–B4	barriers 1–4
BICR	blinded independent central review
*BRCA1/2*	breast cancer susceptibility gene 1/2
BTK	Bruton tyrosine kinase
CA19-9	carbohydrate antigen 19-9
CAF	cancer-associated fibroblast
CAR	chimeric antigen receptor
CCR2	C-C motif chemokine receptor 2
CCR5	C-C motif chemokine receptor 5
CD11b	cluster of differentiation 11b
CD137	cluster of differentiation 137
CD163	cluster of differentiation 163
CD3	cluster of differentiation 3
CD4	cluster of differentiation 4
CD40	cluster of differentiation 40
CD68	cluster of differentiation 68
CD73	cluster of differentiation 73 (ecto-5′-nucleotidase)
CD8	cluster of differentiation 8
cDC1	conventional type 1 dendritic cell
CEA	carcinoembryonic antigen
ChiCTR	Chinese Clinical Trial Registry
CI	confidence interval
CIK	cytokine-induced killer
CPS	combined positive score
CR	complete response
CrI	credible interval
CSF1R	colony-stimulating factor 1 receptor
CTCAE	Common Terminology Criteria for Adverse Events
ctDNA	circulating tumor DNA
CTL	cytotoxic T lymphocyte
CTLA-4	cytotoxic T-lymphocyte-associated protein 4
CXCL1	C-X-C motif chemokine ligand 1
CXCL12	C-X-C motif chemokine ligand 12
CXCR2	C-X-C motif chemokine receptor 2
CXCR4	C-X-C motif chemokine receptor 4
Cy	cyclophosphamide
CyTOF	mass cytometry by time of flight
DCR	disease-control rate
DDR	DNA damage repair
DFS	disease-free survival
DLT	dose-limiting toxicity
dMMR	mismatch repair deficient
DNA	deoxyribonucleic acid
DoR	duration of response
DTH	delayed-type hypersensitivity
ELISA	enzyme-linked immunosorbent assay
ELISPOT	enzyme-linked immunospot
EORTC QLQ-C30	European Organization for Research and Treatment of Cancer Quality of Life Questionnaire Core 30
EORTC QLQ-PAN26	European Organization for Research and Treatment of Cancer Quality of Life Questionnaire Pancreatic Cancer Module 26
ESMO	European Society for Medical Oncology
FAK	focal adhesion kinase
*FANCA*	Fanconi anemia complementation group A
FAS	full analysis set
FDA	Food and Drug Administration
FFPE	formalin-fixed, paraffin-embedded
FOLFIRINOX	fluorouracil, leucovorin (folinic acid), irinotecan, and oxaliplatin
GM-CSF	granulocyte–macrophage colony-stimulating factor
GVAX	granulocyte–macrophage colony-stimulating factor-secreting allogeneic tumor cell vaccine
HDAC	histone deacetylase
HLA	human leukocyte antigen
HLA-DR	human leukocyte antigen DR isotype
HR	hazard ratio
HRD	homologous recombination deficiency
ICTRP	International Clinical Trials Registry Platform
IFN-γ	interferon gamma
IHC	immunohistochemistry
IL-6	interleukin 6
IRE	irreversible electroporation
iRECIST	Immunotherapy Response Evaluation Criteria in Solid Tumors
ISRCTN	International Standard Randomized Controlled Trial Number
ITT	intention to treat
*KRAS*	Kirsten rat sarcoma viral oncogene homolog
LAG-3	lymphocyte-activation gene 3
LDH	lactate dehydrogenase
LMR	lymphocyte-to-monocyte ratio
*LRRC15*	leucine-rich repeat containing 15
Mb	megabase
MDSC	myeloid-derived suppressor cell
mFOLFOX6	modified fluorouracil, leucovorin (folinic acid), and oxaliplatin regimen 6
MHC	major histocompatibility complex
MHC-I	major histocompatibility complex class I
mIF	multiplex immunofluorescence
mIHC	multiplexed immunohistochemistry
MINORS	methodological index for nonrandomized studies
mITT	modified intention to treat
MMR	mismatch repair
MSI	microsatellite instability
MSI-H	microsatellite instability-high
MTD	maximum tolerated dose
NALIRIFOX	liposomal irinotecan, fluorouracil, leucovorin, and oxaliplatin
NCBI	National Center for Biotechnology Information
NCT	ClinicalTrials.gov trial registration identifier
NGS	next-generation sequencing
NK	natural killer
NLR	neutrophil-to-lymphocyte ratio
ORR	objective response rate
OS	overall survival
*PALB2*	partner and localizer of *BRCA2*
PARP	poly(adenosine diphosphate-ribose) polymerase
PBMC	peripheral blood mononuclear cell
PD-1	programmed cell death protein 1
PDAC	pancreatic ductal adenocarcinoma
PD-L1	programmed death-ligand 1
PFS	progression-free survival
PLR	platelet-to-lymphocyte ratio
*POLE*	DNA polymerase epsilon, catalytic subunit
PR	partial response
PRISMA	Preferred Reporting Items for Systematic Reviews and Meta-Analyses
QLQ-C30	Quality of Life Questionnaire Core 30
QLQ-PAN26	Quality of Life Questionnaire Pancreatic Cancer Module 26
QoL	quality of life
RECIST	Response Evaluation Criteria in Solid Tumors
RNA	ribonucleic acid
RoB 2	revised Cochrane risk-of-bias tool for randomized trials
ROBINS-I	Risk Of Bias In Nonrandomized Studies of Interventions
ROI	region of interest
RP2D	recommended phase 2 dose
SANRA	Scale for the Assessment of Narrative Review Articles
SBRT	stereotactic body radiotherapy
SWOG	SWOG Cancer Research Network
TAM	tumor-associated macrophage
TCR	T-cell receptor
TEAE	treatment-emergent adverse event
TGF-β	transforming growth factor beta
TIGIT	T-cell immunoreceptor with immunoglobulin and immunoreceptor tyrosine-based inhibitory motif domains
TIL	tumor-infiltrating lymphocyte
TIM-3	T-cell immunoglobulin and mucin domain-containing protein 3
TMB	tumor mutational burden
TNF	tumor necrosis factor
TNFR2	tumor necrosis factor receptor 2
TOX	thymocyte selection-associated high mobility group box protein
TPS	tumor proportion score
TRAE	treatment-related adverse event
T1–T4	evidence tiers 1–4 (author-defined design descriptors; Section 2.4)
Treg	regulatory T cell
TTP	time to progression
UMIN	University hospital Medical Information Network
US	United States
VEGFR2	vascular endothelial growth factor receptor 2
WES	whole-exome sequencing
WHO	World Health Organization
WT1	Wilms tumor 1

## References

[1] Siegel R.L., Kratzer T.B., Wagle N.S., Sung H., Jemal A. (2026). Cancer statistics, 2026. CA Cancer J. Clin..

[2] Conroy T., Desseigne F., Ychou M., Bouché O., Guimbaud R., Bécouarn Y., Adenis A., Raoul J.L., Gourgou-Bourgade S., de la Fouchardière C. (2011). FOLFIRINOX versus Gemcitabine for Metastatic Pancreatic Cancer. N. Engl. J. Med..

[3] Von Hoff D.D., Ervin T., Arena F.P., Chiorean E.G., Infante J., Moore M., Seay T., Tjulandin S.A., Ma W.W., Saleh M.N. (2013). Increased Survival in Pancreatic Cancer with nab-Paclitaxel plus Gemcitabine. N. Engl. J. Med..

[4] Wainberg Z.A., Melisi D., Macarulla T., Pazo Cid R., Chandana S.R., De La Fouchardière C., Dean A., Kiss I., Lee W.J., Goetze T.O. (2023). NALIRIFOX versus nab-paclitaxel and gemcitabine in treatment-naive patients with metastatic pancreatic ductal adenocarcinoma (NAPOLI 3): A randomised, open-label, phase 3 trial. Lancet.

[5] Sherman M.H., Beatty G.L. (2023). Tumor Microenvironment in Pancreatic Cancer Pathogenesis and Therapeutic Resistance. Annu. Rev. Pathol..

[6] Royal R.E., Levy C., Turner K., Mathur A., Hughes M., Kammula U.S., Sherry R.M., Topalian S.L., Yang J.C., Lowy I. (2010). Phase 2 Trial of Single Agent Ipilimumab (Anti-CTLA-4) for Locally Advanced or Metastatic Pancreatic Adenocarcinoma. J. Immunother..

[7] Brahmer J.R., Tykodi S.S., Chow L.Q.M., Hwu W.J., Topalian S.L., Hwu P., Drake C.G., Camacho L.H., Kauh J., Odunsi K. (2012). Safety and Activity of Anti–PD-L1 Antibody in Patients with Advanced Cancer. N. Engl. J. Med..

[8] O’Reilly E.M., Oh D.Y., Dhani N., Renouf D.J., Lee M.A., Sun W., Fisher G., Hezel A., Chang S.C., Vlahovic G. (2019). Durvalumab with or Without Tremelimumab for Patients with Metastatic Pancreatic Ductal Adenocarcinoma: A Phase 2 Randomized Clinical Trial. JAMA Oncol..

[9] Marabelle A., Le D.T., Ascierto P.A., Di Giacomo A.M., De Jesus-Acosta A., Delord J.P., Geva R., Gottfried M., Penel N., Hansen A.R. (2020). Efficacy of Pembrolizumab in Patients with Noncolorectal High Microsatellite Instability/Mismatch Repair–Deficient Cancer: Results From the Phase II KEYNOTE-158 Study. J. Clin. Oncol..

[10] Kaplon H. (2022). Translational Learnings in the Development of Chemo-Immunotherapy Combination to Bypass the Cold Tumor Microenvironment in Pancreatic Ductal Adenocarcinoma. Front. Oncol..

[11] Shakiba M., Tuveson D.A. (2025). Macrophages and fibroblasts as regulators of the immune response in pancreatic cancer. Nat. Immunol..

[12] Ju Y., Xu D., Liao M.M., Sun Y., Bao W.D., Yao F., Ma L. (2024). Barriers and opportunities in pancreatic cancer immunotherapy. npj Precis. Oncol..

[13] Hilmi M., Delaye M., Muzzolini M., Nicolle R., Cros J., Hammel P., Cardot-Ruffino V., Neuzillet C. (2023). The immunological landscape in pancreatic ductal adenocarcinoma and overcoming resistance to immunotherapy. Lancet Gastroenterol. Hepatol..

[14] Farhangnia P., Khorramdelazad H., Nickho H., Delbandi A.A. (2024). Current and future immunotherapeutic approaches in pancreatic cancer treatment. J. Hematol. Oncol..

[15] Al-Khinji A., Al-Korbi N., Al-Kuwari S., Al-Hor A., Malouche D. (2025). Immune checkpoint inhibitors in pancreatic adenocarcinoma: A systematic review and meta analysis of clinical outcomes. Front. Oncol..

[16] Qi X., Yang H., Zhang L., Yu P., Cheng C. (2026). Advancing precision immunotherapy in advanced pancreatic cancer: A systematic review and meta-analysis of first-line ICI-based combinations. Front. Immunol..

[17] OCEBM Levels of Evidence Working Group (2011). The Oxford 2011 Levels of Evidence. https://www.cebm.ox.ac.uk/resources/levels-of-evidence/ocebm-levels-of-evidence.

[18] Sterne J.A.C., Savović J., Page M.J., Elbers R.G., Blencowe N.S., Boutron I., Cates C.J., Cheng H.Y., Corbett M.S., Eldridge S.M. (2019). RoB 2: A revised tool for assessing risk of bias in randomised trials. BMJ.

[19] Sterne J.A.C., Hernán M.A., Reeves B.C., Savović J., Berkman N.D., Viswanathan M., Henry D., Altman D.G., Ansari M.T., Boutron I. (2016). ROBINS-I: A tool for assessing risk of bias in non-randomised studies of interventions. BMJ.

[20] Munn Z., Barker T.H., Moola S., Tufanaru C., Stern C., McArthur A., Stephenson M., Aromataris E. (2020). Methodological quality of case series studies: An introduction to the JBI critical appraisal tool. JBI Evid. Synth..

[21] Slim K., Nini E., Forestier D., Kwiatkowski F., Panis Y., Chipponi J. (2003). Methodological index for non-randomized studies (MINORS): Development and validation of a new instrument. ANZ J. Surg..

[22] Baethge C., Goldbeck-Wood S., Mertens S. (2019). SANRA—A scale for the quality assessment of narrative review articles. Res. Integr. Peer Rev..

[23] de Santiago I., Yau C., Heij L., Middleton M.R., Markowetz F., Grabsch H.I., Dustin M.L., Sivakumar S. (2019). Immunophenotypes of pancreatic ductal adenocarcinoma: Meta-analysis of transcriptional subtypes. Int. J. Cancer.

[24] Liu H., Chen M., Hong B., Xiao Y., Chen Q., Qian Y. (2025). Single-nucleus RNA sequencing and spatial transcriptomics reveal an immunosuppressive tumor microenvironment related to metastatic dissemination during pancreatic cancer liver metastasis. Theranostics.

[25] Khaliq A.M., Rajamohan M., Saeed O., Mansouri K., Adil A., Zhang C., Turk A., Carstens J.L., House M., Hayat S. (2024). Spatial transcriptomic analysis of primary and metastatic pancreatic cancers highlights tumor microenvironmental heterogeneity. Nat. Genet..

[26] Beatty G.L., Werba G., Lyssiotis C.A., Simeone D.M. (2021). The biological underpinnings of therapeutic resistance in pancreatic cancer. Genes Dev..

[27] Balachandran V.P., Łuksza M., Zhao J.N., Makarov V., Moral J.A., Remark R., Herbst B., Askan G., Bhanot U., Senbabaoglu Y. (2017). Identification of unique neoantigen qualities in long-term survivors of pancreatic cancer. Nature.

[28] Łuksza M., Sethna Z.M., Rojas L.A., Lihm J., Bravi B., Elhanati Y., Soares K., Amisaki M., Dobrin A., Hoyos D. (2022). Neoantigen quality predicts immunoediting in survivors of pancreatic cancer. Nature.

[29] Yamamoto K., Venida A., Yano J., Biancur D.E., Kakiuchi M., Gupta S., Sohn A.S.W., Mukhopadhyay S., Lin E.Y., Parker S.J. (2020). Autophagy promotes immune evasion of pancreatic cancer by degrading MHC-I. Nature.

[30] Estephan H., Tailor A., Parker R., Kreamer M., Papandreou I., Campo L., Easton A., Moon E.J., Denko N.C., Ternette N. (2025). Hypoxia promotes tumor immune evasion by suppressing MHC-I expression and antigen presentation. EMBO J..

[31] Hegde S., Krisnawan V.E., Herzog B.H., Zuo C., Breden M.A., Knolhoff B.L., Hogg G.D., Tang J.P., Baer J.M., Mpoy C. (2020). Dendritic Cell Paucity Leads to Dysfunctional Immune Surveillance in Pancreatic Cancer. Cancer Cell.

[32] Provenzano P.P., Cuevas C., Chang A.E., Goel V.K., Von Hoff D.D., Hingorani S.R. (2012). Enzymatic Targeting of the Stroma Ablates Physical Barriers to Treatment of Pancreatic Ductal Adenocarcinoma. Cancer Cell.

[33] Jacobetz M.A., Chan D.S., Neesse A., Bapiro T.E., Cook N., Frese K.K., Feig C., Nakagawa T., Caldwell M.E., Zecchini H.I. (2013). Hyaluronan impairs vascular function and drug delivery in a mouse model of pancreatic cancer. Gut.

[34] Feig C., Jones J.O., Kraman M., Wells R.J.B., Deonarine A., Chan D.S., Connell C.M., Roberts E.W., Zhao Q., Caballero O.L. (2013). Targeting CXCL12 from FAP-expressing carcinoma-associated fibroblasts synergizes with anti–PD-L1 immunotherapy in pancreatic cancer. Proc. Natl. Acad. Sci. USA.

[35] Ene-Obong A., Clear A.J., Watt J., Wang J., Fatah R., Riches J.C., Marshall J.F., Chin-Aleong J., Chelala C., Gribben J.G. (2013). Activated Pancreatic Stellate Cells Sequester CD8+ T Cells to Reduce Their Infiltration of the Juxtatumoral Compartment of Pancreatic Ductal Adenocarcinoma. Gastroenterology.

[36] Özdemir B.C., Pentcheva-Hoang T., Carstens J.L., Zheng X., Wu C.C., Simpson T.R., Laklai H., Sugimoto H., Kahlert C., Novitskiy S.V. (2014). Depletion of Carcinoma-Associated Fibroblasts and Fibrosis Induces Immunosuppression and Accelerates Pancreas Cancer with Reduced Survival. Cancer Cell.

[37] Rhim A.D., Oberstein P.E., Thomas D.H., Mirek E.T., Palermo C.F., Sastra S.A., Dekleva E.N., Saunders T., Becerra C.P., Tattersall I.W. (2014). Stromal Elements Act to Restrain, Rather Than Support, Pancreatic Ductal Adenocarcinoma. Cancer Cell.

[38] Elyada E., Bolisetty M., Laise P., Flynn W.F., Courtois E.T., Burkhart R.A., Teinor J.A., Belleau P., Biffi G., Lucito M.S. (2019). Cross-Species Single-Cell Analysis of Pancreatic Ductal Adenocarcinoma Reveals Antigen-Presenting Cancer-Associated Fibroblasts. Cancer Discov..

[39] Sherman M.H., Pasca di Magliano M. (2023). Cancer-Associated Fibroblasts: Lessons from Pancreatic Cancer. Annu. Rev. Cancer Biol..

[40] Chen Y., Kim J., Yang S., Wang H., Wu C.J., Sugimoto H., LeBleu V.S., Kalluri R. (2021). Type I collagen deletion in αSMA+ myofibroblasts augments immune suppression and accelerates progression of pancreatic cancer. Cancer Cell.

[41] Masugi Y. (2022). The Desmoplastic Stroma of Pancreatic Cancer: Multilayered Levels of Heterogeneity, Clinical Significance, and Therapeutic Opportunities. Cancers.

[42] Steele C.W., Karim S.A., Leach J.D.G., Bailey P., Upstill-Goddard R., Rishi L., Foth M., Bryson S., McDaid K., Wilson Z. (2016). CXCR2 Inhibition Profoundly Suppresses Metastases and Augments Immunotherapy in Pancreatic Ductal Adenocarcinoma. Cancer Cell.

[43] Gorchs L., Oosthoek M., Yucel-Lindberg T., Moro C.F., Kaipe H. (2022). Chemokine Receptor Expression on T Cells Is Modulated by CAFs and Chemokines Affect the Spatial Distribution of T Cells in Pancreatic Tumors. Cancers.

[44] Carstens J.L., Correa de Sampaio P., Yang D., Barua S., Wang H., Rao A., Allison J.P., LeBleu V.S., Kalluri R. (2017). Spatial computation of intratumoral T cells correlates with survival of patients with pancreatic cancer. Nat. Commun..

[45] Koong A.C., Mehta V.K., Le Q.T., Fisher G.A., Terris D.J., Brown J.M., Bastidas A.J., Vierra M. (2000). Pancreatic tumors show high levels of hypoxia. Int. J. Radiat. Oncol. Biol. Phys..

[46] Shah V.M., Sheppard B.C., Sears R.C., Alani A.W.G. (2020). Hypoxia: Friend or Foe for drug delivery in Pancreatic Cancer. Cancer Lett..

[47] Bayne L.J., Beatty G.L., Jhala N., Clark C.E., Rhim A.D., Stanger B.Z., Vonderheide R.H. (2012). Tumor-Derived Granulocyte-Macrophage Colony-Stimulating Factor Regulates Myeloid Inflammation and T Cell Immunity in Pancreatic Cancer. Cancer Cell.

[48] Pylayeva-Gupta Y., Lee K.E., Hajdu C.H., Miller G., Bar-Sagi D. (2012). Oncogenic Kras-Induced GM-CSF Production Promotes the Development of Pancreatic Neoplasia. Cancer Cell.

[49] Nywening T.M., Belt B.A., Cullinan D.R., Panni R.Z., Han B.J., Sanford D.E., Jacobs R.C., Ye J., Patel A.A., Gillanders W.E. (2018). Targeting both tumour-associated CXCR2+ neutrophils and CCR2+ macrophages disrupts myeloid recruitment and improves chemotherapeutic responses in pancreatic ductal adenocarcinoma. Gut.

[50] Zhu Y., Knolhoff B.L., Meyer M.A., Nywening T.M., West B.L., Luo J., Wang-Gillam A., Goedegebuure S.P., Linehan D.C., DeNardo D.G. (2014). CSF1/CSF1R Blockade Reprograms Tumor-Infiltrating Macrophages and Improves Response to T-cell Checkpoint Immunotherapy in Pancreatic Cancer Models. Cancer Res..

[51] Stromnes I.M., Brockenbrough J.S., Izeradjene K., Carlson M.A., Cuevas C., Simmons R.M., Greenberg P.D., Hingorani S.R. (2014). Targeted depletion of an MDSC subset unmasks pancreatic ductal adenocarcinoma to adaptive immunity. Gut.

[52] Bianchi A., De Castro Silva I., Deshpande N.U., Singh S., Mehra S., Garrido V.T., Guo X., Nivelo L.A., Kolonias D.S., Saigh S.J. (2023). Cell-Autonomous Cxcl1 Sustains Tolerogenic Circuitries and Stromal Inflammation via Neutrophil-Derived TNF in Pancreatic Cancer. Cancer Discov..

[53] Khan O., Giles J.R., McDonald S., Manne S., Ngiow S.F., Patel K.P., Werner M.T., Huang A.C., Alexander K.A., Wu J.E. (2019). TOX transcriptionally and epigenetically programs CD8+ T cell exhaustion. Nature.

[54] Wherry E.J., Kurachi M. (2015). Molecular and cellular insights into T cell exhaustion. Nat. Rev. Immunol..

[55] Ajina R., Weiner L.M. (2020). T-Cell Immunity in Pancreatic Cancer. Pancreas.

[56] Nakayama C., Tanoue K., Idichi T., Shimomura H., Kita Y., Hozaka Y., Shinden Y., Matsushita D., Nakajo A., Arigami T. (2022). Implications of PD-1, Tim-3, and TIGIT Expression for Cancer Immunity and Pancreatic Cancer Prognosis. Anticancer Res..

[57] Seifert L., Plesca I., Müller L., Sommer U., Heiduk M., von Renesse J., Digomann D., Glück J., Klimova A., Weitz J. (2021). LAG-3-Expressing Tumor-Infiltrating T Cells Are Associated with Reduced Disease-Free Survival in Pancreatic Cancer. Cancers.

[58] Quatannens D., Verhoeven Y., van der Heijden S., Peeters D., Claes J., Hermans C., Lambrechts H., Zwaenepoel K., van Dam P., Peeters M. (2026). Protein-level profiling of TIGIT axis components in human PDAC reveals immune–suppressive expression patterns. Cancer Immunol. Immunother..

[59] Steele N.G., Carpenter E.S., Kemp S.B., Sirihorachai V.R., The S., Delrosario L., Lazarus J., Amir E.A.D., Gunchick V., Espinoza C. (2020). Multimodal mapping of the tumor and peripheral blood immune landscape in human pancreatic cancer. Nat. Cancer.

[60] Hwang W.L., Jagadeesh K.A., Guo J.A., Hoffman H.I., Yadollahpour P., Reeves J.W., Mohan R., Drokhlyansky E., Van Wittenberghe N., Ashenberg O. (2022). Single-nucleus and spatial transcriptome profiling of pancreatic cancer identifies multicellular dynamics associated with neoadjuvant treatment. Nat. Genet..

[61] Grünwald B.T., Devisme A., Andrieux G., Vyas F., Aliar K., McCloskey C.W., Macklin A., Jang G.H., Denroche R., Romero J.M. (2021). Spatially confined sub-tumor microenvironments in pancreatic cancer. Cell.

[62] Chen D.S., Mellman I. (2013). Oncology Meets Immunology: The Cancer-Immunity Cycle. Immunity.

[63] Chen D.S., Mellman I. (2017). Elements of cancer immunity and the cancer–immune set point. Nature.

[64] Mellman I., Chen D.S., Powles T., Turley S.J. (2023). The cancer-immunity cycle: Indication, genotype, and immunotype. Immunity.

[65] Diamond M.S., Lin J.H., Vonderheide R.H. (2021). Site-Dependent Immune Escape Due to Impaired Dendritic Cell Cross-Priming. Cancer Immunol. Res..

[66] Morrison A.H., Diamond M.S., Hay C.A., Byrne K.T., Vonderheide R.H. (2020). Sufficiency of CD40 activation and immune checkpoint blockade for T cell priming and tumor immunity. Proc. Natl. Acad. Sci. USA.

[67] Gulhati P., Schalck A., Jiang S., Shang X., Wu C.J., Hou P., Ruiz S.H., Soto L.S., Parra E., Ying H. (2023). Targeting T cell checkpoints 41BB and LAG3 and myeloid cell CXCR1/CXCR2 results in antitumor immunity and durable response in pancreatic cancer. Nat. Cancer.

[68] Stone M.L., Herrera V.M., Li Y., Coho H., Xue Y., Graham K., Ratmansky J., Delman D., O’Brien S., Beatty G.L. (2025). Nonredundant Immune Checkpoints Direct Therapeutic Resistance to Chemoimmunotherapy in Pancreatic Ductal Adenocarcinoma. Cancer Immunol. Res..

[69] Jiang H., Hegde S., Knolhoff B.L., Zhu Y., Herndon J.M., Meyer M.A., Nywening T.M., Hawkins W.G., Shapiro I.M., Weaver D.T. (2016). Targeting focal adhesion kinase renders pancreatic cancers responsive to checkpoint immunotherapy. Nat. Med..

[70] Canel M., Sławińska A.D., Lonergan D.W., Kallor A.A., Upstill-Goddard R., Davidson C., von Kriegsheim A., Biankin A.V., Byron A., Alfaro J. (2024). FAK suppresses antigen processing and presentation to promote immune evasion in pancreatic cancer. Gut.

[71] Bazan-Peregrino M., Garcia-Carbonero R., Laquente B., Álvarez R., Mato-Berciano A., Gimenez-Alejandre M., Morgado S., Rodríguez-García A., Maliandi M.V., Riesco M.C. (2021). VCN-01 disrupts pancreatic cancer stroma and exerts antitumor effects. J. Immunother. Cancer.

[72] Van Cutsem E., Tempero M.A., Sigal D., Oh D.Y., Fazio N., Macarulla T., Hitre E., Hammel P., Hendifar A.E., Bates S.E. (2020). Randomized Phase III Trial of Pegvorhyaluronidase Alfa with Nab-Paclitaxel Plus Gemcitabine for Patients with Hyaluronan-High Metastatic Pancreatic Adenocarcinoma. J. Clin. Oncol..

[73] Musher B.L., Vasileiou S., Smaglo B.G., Robertson C.S., Wu M., Wang T., Watanabe A., Kuvalekar M., Velazquez Y., Ketkar S. (2026). Autologous multiantigen-targeted T cell therapy for pancreatic cancer: A phase 1/2 trial. Nat. Med..

[74] Xu X., Gu H., Cha Z., Zhang B., Yuan S., Ding X., Gao L., Gao S., Wu X., Gu L. (2026). T cell receptor gene therapy targeting KRAS G12V for advanced pancreatic cancer in a single-arm phase 1/2 clinical trial. Mol. Ther..

[75] Seo H., Go D., Jung S.Y., Han S., Nguyen V.Q., Kwak M., Um W. (2025). Bispecific Antibody and Antibody-Drug Conjugate as Novel Candidates for Treating Pancreatic Ductal Adenocarcinoma. Biomolecules.

[76] Renouf D.J., Loree J.M., Knox J.J., Topham J.T., Kavan P., Jonker D., Welch S., Couture F., Lemay F., Tehfe M. (2022). The CCTG PA.7 phase II trial of gemcitabine and nab-paclitaxel with or without durvalumab and tremelimumab as initial therapy in metastatic pancreatic ductal adenocarcinoma. Nat. Commun..

[77] Wu A.A., Bever K.M., Ho W.J., Fertig E.J., Niu N., Zheng L., Parkinson R.M., Durham J.N., Onners B., Ferguson A.K. (2020). A Phase II Study of Allogeneic GM-CSF–Transfected Pancreatic Tumor Vaccine (GVAX) with Ipilimumab as Maintenance Treatment for Metastatic Pancreatic Cancer. Clin. Cancer Res..

[78] O’Hara M.H., O’Reilly E.M., Varadhachary G., Wolff R.A., Wainberg Z.A., Ko A.H., Fisher G., Rahma O., Lyman J.P., Cabanski C.R. (2021). CD40 agonistic monoclonal antibody APX005M (sotigalimab) and chemotherapy, with or without nivolumab, for the treatment of metastatic pancreatic adenocarcinoma: An open-label, multicentre, phase 1b study. Lancet Oncol..

[79] Padrón L.J., Maurer D.M., O’Hara M.H., O’Reilly E.M., Wolff R.A., Wainberg Z.A., Ko A.H., Fisher G., Rahma O., Lyman J.P. (2022). Sotigalimab and/or nivolumab with chemotherapy in first-line metastatic pancreatic cancer: Clinical and immunologic analyses from the randomized phase 2 PRINCE trial. Nat. Med..

[80] Van Laethem J.L., Borbath I., Prenen H., Geboes K.P., Lambert A., Mitry E., Cassier P.A., Blanc J.F., Pilla L., Batlle J.F. (2024). Combining CD40 agonist mitazalimab with mFOLFIRINOX in previously untreated metastatic pancreatic ductal adenocarcinoma (OPTIMIZE-1): A single-arm, multicentre phase 1b/2 study. Lancet Oncol..

[81] Middleton G., Silcocks P., Cox T., Valle J., Wadsley J., Propper D., Coxon F., Ross P., Madhusudan S., Roques T. (2014). Gemcitabine and capecitabine with or without telomerase peptide vaccine GV1001 in patients with locally advanced or metastatic pancreatic cancer (TeloVac): An open-label, randomised, phase 3 trial. Lancet Oncol..

[82] Hewitt D.B., Nissen N., Hatoum H., Musher B., Seng J., Coveler A.L., Al-Rajabi R., Yeo C.J., Leiby B., Banks J. (2022). A Phase 3 Randomized Clinical Trial of Chemotherapy with or Without Algenpantucel-L (HyperAcute-Pancreas) Immunotherapy in Subjects with Borderline Resectable or Locally Advanced Unresectable Pancreatic Cancer. Ann. Surg..

[83] Rojas L.A., Sethna Z., Soares K.C., Olcese C., Pang N., Patterson E., Lihm J., Ceglia N., Guasp P., Chu A. (2023). Personalized RNA neoantigen vaccines stimulate T cells in pancreatic cancer. Nature.

[84] Huff A.L., Haldar S.D., Girgis A.A., Wang H.H., Danilova L., Heumann T., Berg M., Wang Y., Andaloori L., Hernandez A. (2026). Mutant KRAS vaccine with dual checkpoint blockade in resected pancreatic cancer: A phase I trial. Nat. Commun..

[85] Jo J.H., Kim Y.T., Choi H.S., Kim H.G., Lee H.S., Choi Y.W., Kim D.U., Lee K.H., Kim E.J., Han J.H. (2024). Efficacy of GV1001 with gemcitabine/capecitabine in previously untreated patients with advanced pancreatic ductal adenocarcinoma having high serum eotaxin levels (KG4/2015): An open-label, randomised, Phase 3 trial. Br. J. Cancer.

[86] Yamaue H., Tsunoda T., Tani M., Miyazawa M., Yamao K., Mizuno N., Okusaka T., Ueno H., Boku N., Fukutomi A. (2015). Randomized phase II/III clinical trial of elpamotide for patients with advanced pancreatic cancer: PEGASUS-PC Study. Cancer Sci..

[87] Noonan A.M., Farren M.R., Geyer S.M., Huang Y., Tahiri S., Ahn D., Mikhail S., Ciombor K.K., Pant S., Aparo S. (2016). Randomized Phase 2 Trial of the Oncolytic Virus Pelareorep (Reolysin) in Upfront Treatment of Metastatic Pancreatic Adenocarcinoma. Mol. Ther..

[88] Mahalingam D., Wilkinson G.A., Eng K.H., Fields P., Raber P., Moseley J.L., Cheetham K., Coffey M., Nuovo G., Kalinski P. (2020). Pembrolizumab in Combination with the Oncolytic Virus Pelareorep and Chemotherapy in Patients with Advanced Pancreatic Adenocarcinoma: A Phase Ib Study. Clin. Cancer Res..

[89] Musher B.L., Rowinsky E.K., Smaglo B.G., Abidi W., Othman M., Patel K., Jawaid S., Jing J., Brisco A., Leen A.M. (2024). LOAd703, an oncolytic virus-based immunostimulatory gene therapy, combined with chemotherapy for unresectable or metastatic pancreatic cancer (LOKON001): Results from arm 1 of a non-randomised, single-centre, phase 1/2 study. Lancet Oncol..

[90] Dalgleish A.G., Stebbing J., Adamson D.J.A., Arif S.S., Bidoli P., Chang D., Cheeseman S., Diaz-Beveridge R., Fernandez-Martos C., Glynne-Jones R. (2016). Randomised, open-label, phase II study of gemcitabine with and without IMM-101 for advanced pancreatic cancer. Br. J. Cancer.

[91] Jing W., McAllister D., Vonderhaar E.P., Palen K., Riese M.J., Gershan J., Johnson B.D., Dwinell M.B. (2019). STING agonist inflames the pancreatic cancer immune microenvironment and reduces tumor burden in mouse models. J. Immunother. Cancer.

[92] Ye J., Mills B.N., Qin S.S., Garrett-Larsen J., Murphy J.D., Uccello T.P., Han B.J., Vrooman T.G., Johnston C.J., Lord E.M. (2022). Toll-like receptor 7/8 agonist R848 alters the immune tumor microenvironment and enhances SBRT-induced antitumor efficacy in murine models of pancreatic cancer. J. Immunother. Cancer.

[93] Chen I.M., Johansen J.S., Theile S., Hjaltelin J.X., Novitski S.I., Brunak S., Hasselby J.P., Willemoe G.L., Lorentzen T., Madsen K. (2022). Randomized Phase II Study of Nivolumab with or Without Ipilimumab Combined with Stereotactic Body Radiotherapy for Refractory Metastatic Pancreatic Cancer (CheckPAC). J. Clin. Oncol..

[94] Xie C., Duffy A.G., Brar G., Fioravanti S., Mabry-Hrones D., Walker M., Bonilla C.M., Wood B.J., Citrin D.E., Gil Ramirez E.M. (2020). Immune Checkpoint Blockade in Combination with Stereotactic Body Radiotherapy in Patients with Metastatic Pancreatic Ductal Adenocarcinoma. Clin. Cancer Res..

[95] Wang-Gillam A., Lim K.H., McWilliams R., Suresh R., Lockhart A.C., Brown A., Breden M., Belle J.I., Herndon J., Bogner S.J. (2022). Defactinib, Pembrolizumab, and Gemcitabine in Patients with Advanced Treatment Refractory Pancreatic Cancer: A Phase I Dose Escalation and Expansion Study. Clin. Cancer Res..

[96] Ko A.H., Kim K.P., Siveke J.T., Lopez C.D., Lacy J., O’Reilly E.M., Macarulla T., Manji G.A., Lee J., Ajani J. (2023). Atezolizumab Plus PEGPH20 Versus Chemotherapy in Advanced Pancreatic Ductal Adenocarcinoma and Gastric Cancer: MORPHEUS Phase Ib/II Umbrella Randomized Study Platform. Oncologist.

[97] Chiorean E.G., Damle S.R., Zhen D.B., Whittle M., George B., Hochster H., Coveler A.L., Hendifar A., Dragovich T., Safyan R.A. (2026). Phase II Study of Pegvorhyaluronidase Alfa (PEGPH20) and Pembrolizumab for Patients with Hyaluronan-High, Pretreated Metastatic Pancreatic Ductal Adenocarcinoma: PCRT16-001. Cancers.

[98] Bockorny B., Semenisty V., Macarulla T., Borazanci E., Wolpin B.M., Stemmer S.M., Golan T., Geva R., Borad M.J., Pedersen K.S. (2020). BL-8040, a CXCR4 antagonist, in combination with pembrolizumab and chemotherapy for pancreatic cancer: The COMBAT trial. Nat. Med..

[99] Bockorny B., Macarulla T., Semenisty V., Borazanci E., Feliu J., Ponz-Sarvise M., Abad D.G., Oberstein P., Alistar A., Muñoz A. (2021). Motixafortide and Pembrolizumab Combined to Nanoliposomal Irinotecan, Fluorouracil, and Folinic Acid in Metastatic Pancreatic Cancer: The COMBAT/KEYNOTE-202 Trial. Clin. Cancer Res..

[100] Manji G.A., Chung V., Oh D.Y., Lacy J., Lopez C.D., Ponz-Sarvisé M., Macarulla T., Thomas R., George B., Alistar A. (2026). Atezolizumab and motixafortide, cobimetinib or simlukafusp alfa in pretreated advanced pancreatic cancer: Phase I/IIb MORPHEUS-PDAC umbrella study. Oncologist.

[101] Baretti M., Danilova L., Durham J.N., Betts C.B., Cope L., Sidiropoulos D.N., Tandurella J.A., Charmsaz S., Gross N., Hernandez A. (2024). Entinostat in combination with nivolumab in metastatic pancreatic ductal adenocarcinoma: A phase 2 clinical trial. Nat. Commun..

[102] Melisi D., Oh D.Y., Hollebecque A., Calvo E., Varghese A., Borazanci E., Macarulla T., Merz V., Zecchetto C., Zhao Y. (2021). Safety and activity of the TGFβ receptor I kinase inhibitor galunisertib plus the anti-PD-L1 antibody durvalumab in metastatic pancreatic cancer. J. Immunother. Cancer.

[103] Le D.T., Folprecht G., Varghese A.M., Gutierrez M., Noel M., Trikalinos N.A., Chen E., Dayyani F., Davis S.L., Ma W.W. (2026). Phase 1b/2 study of BMS-813160, a CCR2/5 dual antagonist, in combination with chemotherapy or nivolumab in patients with advanced pancreatic or colorectal cancer. J. Immunother. Cancer.

[104] Melisi D., Garcia-Carbonero R., Macarulla T., Pezet D., Deplanque G., Fuchs M., Trojan J., Oettle H., Kozloff M., Cleverly A. (2018). Galunisertib plus gemcitabine vs. gemcitabine for first-line treatment of patients with unresectable pancreatic cancer. Br. J. Cancer.

[105] Wainberg Z.A., Manji G.A., Bahary N., Ulahannan S.V., Pant S., Spigel D.R., Uboha N.V., Oberstein P.E., Saeed A., Beagle B. (2026). Quemliclustat and chemotherapy with or without zimberelimab in metastatic pancreatic adenocarcinoma: A randomized phase 1 trial. Nat. Med..

[106] Coveler A.L., Reilley M.J., Zalupski M., Macarulla T., Fountzilas C., Ponz-Sarvisé M., Nagrial A., Uboha N.V., Frentzas S., Overman M. (2024). A Phase Ib/II Randomized Clinical Trial of Oleclumab with or without Durvalumab plus Chemotherapy in Patients with Metastatic Pancreatic Ductal Adenocarcinoma. Clin. Cancer Res..

[107] Lin M., Zhang X., Liang S., Luo H., Alnaggar M., Liu A., Yin Z., Chen J., Niu L., Jiang Y. (2020). Irreversible electroporation plus allogenic Vγ9Vδ2 T cells enhances antitumor effect for locally advanced pancreatic cancer patients. Signal Transduct. Target. Ther..

[108] Wang M., Shi S.B., Qi J.L., Tang X.Y., Tian J. (2013). S-1 plus CIK as second-line treatment for advanced pancreatic cancer. Med. Oncol..

[109] Principe D.R., Korc M., Kamath S.D., Munshi H.G., Rana A. (2021). Trials and tribulations of pancreatic cancer immunotherapy. Cancer Lett..

[110] Renouf D.J., Topham J.T., Loree J.M., Schaeffer D.F., Knox J.J., Kavan P., Jonker D., Welch S., Couture F., Lemay F. (2026). DNA Repair gene alterations and efficacy from gemcitabine and nab-paclitaxel with/without durvalumab and tremelimumab in metastatic pancreatic ductal adenocarcinoma. Nat. Commun..

[111] Fu Q., Chen Y., Huang D., Guo C., Zhang X., Xiao W., Xue X., Zhang Q., Li X., Gao S. (2023). Sintilimab Plus Modified FOLFIRINOX in Metastatic or Recurrent Pancreatic Cancer: The Randomized Phase II CISPD3 Trial. Ann. Surg. Oncol..

[112] Jia R., Si H.Y., Fan M.J., Zhang N., Deng G.C., Liu F.F., Han L., Gou M.M., Tan Z.L., Zhang X. (2025). First-line treatment with chemotherapy, surufatinib (an angio-immuno kinase inhibitor), and camrelizumab (an anti-PD-1 antibody) for locally advanced or metastatic pancreatic ductal adenocarcinoma: A phase Ib/II randomized study. Signal Transduct. Target. Ther..

[113] Weiss G.J., Blaydorn L., Beck J., Bornemann-Kolatzki K., Urnovitz H., Schütz E., Khemka V. (2018). Phase Ib/II study of gemcitabine, nab-paclitaxel, and pembrolizumab in metastatic pancreatic adenocarcinoma. Investig. New Drugs.

[114] Morizane C., Ueno M., Ikeda M., Sudo K., Hirashima Y., Kuroda M., Ueno S., Okusaka T., Furuse J. (2024). A Phase 2 study of nivolumab in combination with modified FOLFIRINOX for metastatic pancreatic cancer. BJC Rep..

[115] Wainberg Z.A., Link J.M., Premji A., Zheng S., Srienc M., Hammons M., Kim S.E., Li L., Liu Z., Tsvetkova O. (2026). Neoadjuvant modified FOLFIRINOX plus nivolumab in borderline-resectable pancreatic ductal adenocarcinoma: A pilot phase 1 trial. Nat. Commun..

[116] Wainberg Z.A., Hochster H.S., Kim E.J., George B., Kaylan A., Chiorean E.G., Waterhouse D.M., Guiterrez M., Parikh A., Jain R. (2020). Open-label, Phase I Study of Nivolumab Combined with nab-Paclitaxel Plus Gemcitabine in Advanced Pancreatic Cancer. Clin. Cancer Res..

[117] Aglietta M., Barone C., Sawyer M.B., Moore M.J., Miller W.H., Bagalà C., Colombi F., Cagnazzo C., Gioeni L., Wang E. (2014). A phase I dose escalation trial of tremelimumab (CP-675,206) in combination with gemcitabine in chemotherapy-naive patients with metastatic pancreatic cancer. Ann. Oncol..

[118] Kamath S.D., Kalyan A., Kircher S., Nimeiri H., Fought A.J., Benson A., Mulcahy M. (2020). Ipilimumab and Gemcitabine for Advanced Pancreatic Cancer: A Phase Ib Study. Oncologist.

[119] Van Laethem J.L., Geboes K., Borbath I., Macarulla Mercade T., Lambert A., Cassier P., Prenen H., Mitry E., Blanc J.F., Pilla L. (2025). CD40 agonist mitazalimab with mFOLFIRINOX in untreated metastatic pancreatic cancer: Biomarkers associated with outcomes from OPTIMIZE-1. Cell Rep. Med..

[120] Beatty G.L., Torigian D.A., Chiorean E.G., Saboury B., Brothers A., Alavi A., Troxel A.B., Sun W., Teitelbaum U.R., Vonderheide R.H. (2013). A Phase I Study of an Agonist CD40 Monoclonal Antibody (CP-870,893) in Combination with Gemcitabine in Patients with Advanced Pancreatic Ductal Adenocarcinoma. Clin. Cancer Res..

[121] Byrne K.T., Betts C.B., Mick R., Sivagnanam S., Bajor D.L., Laheru D.A., Chiorean E.G., O’Hara M.H., Liudahl S.M., Newcomb C. (2021). Neoadjuvant Selicrelumab, an Agonist CD40 Antibody, Induces Changes in the Tumor Microenvironment in Patients with Resectable Pancreatic Cancer. Clin. Cancer Res..

[122] Pelletier M., Yang J., Joshi M., Grauel A., Abecunas C., Altzerinakou M.A., Zhou Z., Zhu X., Clark K., Li L. (2026). Biomarker Analysis from Patients with Metastatic PDAC Treated with TGFβ Antibody NIS793 plus Abraxane + Gemcitabine versus Abraxane + Gemcitabine Alone in a Phase II, Open-Label, Randomized Study. Clin. Cancer Res..

[123] Mills B.N., Qiu H., Drage M.G., Chen C., Mathew J.S., Garrett-Larsen J., Ye J., Uccello T.P., Murphy J.D., Belt B.A. (2022). Modulation of the Human Pancreatic Ductal Adenocarcinoma Immune Microenvironment by Stereotactic Body Radiotherapy. Clin. Cancer Res..

[124] Kerschbaum-Gruber S., Kleinwächter A., Popova K., Kneringer A., Appel L.M., Stasny K., Röhrer A., Dias A.B., Benedum J., Walch L. (2024). Cytosolic nucleic acid sensors and interferon beta-1 activation drive radiation-induced anti-tumour immune effects in human pancreatic cancer cells. Front. Immunol..

[125] Farren M.R., Sayegh L., Ware M.B., Chen H.R., Gong J., Liang Y., Krasinskas A., Maithel S.K., Zaidi M., Sarmiento J.M. (2020). Immunologic alterations in the pancreatic cancer microenvironment of patients treated with neoadjuvant chemotherapy and radiotherapy. JCI Insight.

[126] Parikh A.R., Szabolcs A., Allen J.N., Clark J.W., Wo J.Y., Raabe M., Thel H., Hoyos D., Mehta A., Arshad S. (2021). Radiation therapy enhances immunotherapy response in microsatellite stable colorectal and pancreatic adenocarcinoma in a phase II trial. Nat. Cancer.

[127] Katz M.H.G., Petroni G.R., Bauer T., Reilley M.J., Wolpin B.M., Stucky C.C., Bekaii-Saab T.S., Elias R., Merchant N., Dias Costa A. (2023). Multicenter randomized controlled trial of neoadjuvant chemoradiotherapy alone or in combination with pembrolizumab in patients with resectable or borderline resectable pancreatic adenocarcinoma. J. Immunother. Cancer.

[128] Chen I.M., Donia M., Chamberlain C.A., Jensen A.W.P., Draghi A., Theile S., Madsen K., Hasselby J.P., Toxværd A., Høgdall E. (2023). Phase 2 study of ipilimumab, nivolumab, and tocilizumab combined with stereotactic body radiotherapy in patients with refractory pancreatic cancer (TRIPLE-R). Eur. J. Cancer.

[129] Vošmik M., John S., Dvořák J., Mohelníková-Duchoňová B., Melichar B., Lohynská R., Ryška A., Banni A.M., Krempová J., Sirák I. (2024). Stereotactic Radiotherapy Plus Nivolumab in Patients with Locally Advanced Pancreatic Cancer: Results from Phase 1/2 Clinical CA209-9KH Trial. Oncol. Ther..

[130] Ni J., Sun Y., Cheng H., Bai X., Tong F., Zhang X., Wang X., Mao L., Li G., Shen S. (2026). Tislelizumab and Hypofractionated Radiotherapy plus Nab-Paclitaxel/Gemcitabine as Conversion Therapy for BRPC/LAPC: A Phase II Trial with Dynamic Biomarker Monitoring. Clin. Cancer Res..

[131] Bever K.M., Shin S.M., Durham J.N., Qi H., Hernandez A., Coyne E.M., Gross N.E., Charmsaz S., Suresh Babu J., Vargas Carvajal D.C. (2025). A phase 2 trial of CXCR4 antagonism and PD1 inhibition in metastatic pancreatic adenocarcinoma reveals recruitment of T cells but also immunosuppressive macrophages. Oncoimmunology.

[132] Nywening T.M., Wang-Gillam A., Sanford D.E., Belt B.A., Panni R.Z., Cusworth B.M., Toriola A.T., Nieman R.K., Worley L.A., Yano M. (2016). Targeting tumour-associated macrophages with CCR2 inhibition in combination with FOLFIRINOX in patients with borderline resectable and locally advanced pancreatic cancer: A single-centre, open-label, dose-finding, non-randomised, phase 1b trial. Lancet Oncol..

[133] Grierson P.M., Wolf C., Suresh R., Wang-Gillam A., Tan B.R., Ratner L., Oppelt P., Aranha O., Frith A., Pedersen K.S. (2025). Neoadjuvant BMS-813160, Nivolumab, Gemcitabine, and Nab-Paclitaxel for Patients with Pancreatic Cancer. Clin. Cancer Res..

[134] Oberstein P.E., Dias Costa A., Kawaler E.A., Cardot-Ruffino V., Rahma O.E., Beri N., Singh H., Abrams T.A., Biller L.H., Cleary J.M. (2024). Blockade of IL1β and PD1 with Combination Chemotherapy Reduces Systemic Myeloid Suppression in Metastatic Pancreatic Cancer with Heterogeneous Effects in the Tumor. Cancer Immunol. Res..

[135] Overman M., Javle M., Davis R.E., Vats P., Kumar-Sinha C., Xiao L., Mettu N.B., Parra E.R., Benson A.B., Lopez C.D. (2020). Randomized phase II study of the Bruton tyrosine kinase inhibitor acalabrutinib, alone or with pembrolizumab in patients with advanced pancreatic cancer. J. Immunother. Cancer.

[136] Ponz-Sarvisé M., Rha S.Y., Gomez-Roca C.A., Ortega Morán L., Gill S., Tortora G., Geva R., Saada-Bouzid E., Santoro A., Kim T.W. (2026). Lenvatinib plus Pembrolizumab for Patients with Previously Treated Advanced Gastric, Biliary Tract, or Pancreatic Cancer: Results from the Phase II LEAP-005 Study. Cancer Res. Commun..

[137] Callahan M., Amin A., Kaye F.J., Morse M.A., Taylor M.H., Peltola K.J., Sharma P., O’Reilly E.M., Meadows Shropshire S., O’Brien S. (2024). Nivolumab monotherapy or combination with ipilimumab with or without cobimetinib in previously treated patients with pancreatic adenocarcinoma (CheckMate 032). J. Immunother. Cancer.

[138] O’Reilly E.M., Cabanski C.R., Lyman J.P., Wainberg Z.A., Fisher G.A., Wolff R.A., Ko A.H., O’Hara M.H., Spencer C.N., Yu J.X. (2026). Clinical and translational results from a phase 1 trial of gemcitabine/nab-paclitaxel with nivolumab/ipilimumab or hydroxychloroquine/ipilimumab in untreated metastatic pancreatic adenocarcinoma. J. Immunother. Cancer.

[139] Sethna Z., Guasp P., Reiche C., Milighetti M., Ceglia N., Patterson E., Lihm J., Payne G., Lyudovyk O., Rojas L.A. (2025). RNA neoantigen vaccines prime long-lived CD8+ T cells in pancreatic cancer. Nature.

[140] Le D.T., Wang-Gillam A., Picozzi V., Greten T.F., Crocenzi T., Springett G., Morse M., Zeh H., Cohen D., Fine R.L. (2015). Safety and Survival with GVAX Pancreas Prime and Listeria Monocytogenes–Expressing Mesothelin (CRS-207) Boost Vaccines for Metastatic Pancreatic Cancer. J. Clin. Oncol..

[141] Tsujikawa T., Crocenzi T., Durham J.N., Sugar E.A., Wu A.A., Onners B., Nauroth J.M., Anders R.A., Fertig E.J., Laheru D.A. (2020). Evaluation of Cyclophosphamide/GVAX Pancreas Followed by Listeria-Mesothelin (CRS-207) with or without Nivolumab in Patients with Pancreatic Cancer. Clin. Cancer Res..

[142] Le D.T., Picozzi V.J., Ko A.H., Wainberg Z.A., Kindler H., Wang-Gillam A., Oberstein P., Morse M.A., Zeh H.J., Weekes C. (2019). Results from a Phase IIb, Randomized, Multicenter Study of GVAX Pancreas and CRS-207 Compared with Chemotherapy in Adults with Previously Treated Metastatic Pancreatic Adenocarcinoma (ECLIPSE Study). Clin. Cancer Res..

[143] Le D.T., Lutz E., Uram J.N., Sugar E.A., Onners B., Solt S., Zheng L., Diaz L.A., Donehower R.C., Jaffee E.M. (2013). Evaluation of Ipilimumab in Combination with Allogeneic Pancreatic Tumor Cells Transfected with a GM-CSF Gene in Previously Treated Pancreatic Cancer. J. Immunother..

[144] Agarwal P., Guo M., Munjal K., Qi H., Parkinson R., Ferguson A., Mitchell C., Harrison J., Anders R.A., Thompson E.D. (2025). A Phase II Study of Neoadjuvant GVAX and Cyclophosphamide Combined with Nivolumab and SBRT Followed by Surgery in Borderline Resectable Pancreatic Adenocarcinoma. Clin. Cancer Res..

[145] Bever K.M., Huff A.L., Danilova L., Wetzel M., Tandurella J.A., Durham J.N., Wang T., Lee J.W., Coyne E.M., Montagne J.M. (2026). A Randomized Phase II Study: CRS207/GVAX plus Anti–PD-1 and Anti-CTLA4 Recruits Mesothelin- and mKRAS-Specific T Cells into PDAC. Cancer Immunol. Res..

[146] Hardacre J.M., Mulcahy M., Small W., Talamonti M., Obel J., Krishnamurthi S., Rocha-Lima C.S., Safran H., Lenz H.J., Chiorean E.G. (2013). Addition of Algenpantucel-L Immunotherapy to Standard Adjuvant Therapy for Pancreatic Cancer: A Phase 2 Study. J. Gastrointest. Surg..

[147] Nishida S., Ishikawa T., Egawa S., Koido S., Yanagimoto H., Ishii J., Kanno Y., Kokura S., Yasuda H., Oba M.S. (2018). Combination Gemcitabine and WT1 Peptide Vaccination Improves Progression-Free Survival in Advanced Pancreatic Ductal Adenocarcinoma: A Phase II Randomized Study. Cancer Immunol. Res..

[148] Shima H., Tsurita G., Wada S., Hirohashi Y., Yasui H., Hayashi H., Miyakoshi T., Watanabe K., Murai A., Asanuma H. (2019). Randomized phase II trial of survivin 2B peptide vaccination for patients with HLA-A24-positive pancreatic adenocarcinoma. Cancer Sci..

[149] Nishida S., Koido S., Takeda Y., Homma S., Komita H., Takahara A., Morita S., Ito T., Morimoto S., Hara K. (2014). Wilms Tumor Gene (WT1) Peptide–based Cancer Vaccine Combined with Gemcitabine for Patients with Advanced Pancreatic Cancer. J. Immunother..

[150] Tan Q., Li Y., Liu C., Xu J., Tong J., Yu J., Huang Y., Hu X., Qin S., Xiao F. (2025). Dynamic transcriptional immune landscape in response to NK-cell therapy combined with gemcitabine plus S-1 in advanced pancreatic cancer: A phase 1b/2 trial. Signal Transduct. Target. Ther..

[151] Aoki T., Matsushita H., Hoshikawa M., Hasegawa K., Kokudo N., Kakimi K. (2017). Adjuvant combination therapy with gemcitabine and autologous γδ T-cell transfer in patients with curatively resected pancreatic cancer. Cytotherapy.

[152] Reiss K.A., Mick R., Teitelbaum U., O’Hara M., Schneider C., Massa R., Karasic T., Tondon R., Onyiah C., Gosselin M.K. (2022). Niraparib plus nivolumab or niraparib plus ipilimumab in patients with platinum-sensitive advanced pancreatic cancer: A randomised, phase 1b/2 trial. Lancet Oncol..

[153] Park W., O’Connor C.A., Chou J.F., Hilmi M., Tarcan Z., Schwartz C., Larsen M., Homsi R., Sivaprakasam K., Umeda S. (2026). Pembrolizumab and olaparib in homologous-recombination-deficient metastatic pancreatic cancer: The phase 2 POLAR trial. Nat. Med..

[154] Chung V., Guthrie K.A., Pishvaian M.J., Lowy A.M., Sohal D., Colby S., Reiss K.A., Khan G., Winograd R., Meyerhardt J.A. (2026). Randomized phase II trial of olaparib and pembrolizumab vs olaparib alone as maintenance therapy in metastatic pancreatic cancer patients with germline BRCA1 or BRCA2 (gBRCA1/2) mutations: SWOG S2001. J. Clin. Oncol..

[155] Enzler T., Nguyen A., Misleh J., Cline V.J., Johns M., Shumway N., Paulson S., Siegel R., Larson T., Messersmith W. (2024). A multicenter, randomized phase 2 study to establish combinations of CBP501, cisplatin and nivolumab for ≥3rd-line treatment of patients with advanced pancreatic adenocarcinoma. Eur. J. Cancer.

[156] Hernando-Calvo A., Han M., Ayodele O., Wang B.X., Bruce J.P., Abbas-Aghababazadeh F., Vila-Casadesús M., Sanz-Garcia E., Yang S.Y.C., Berman H.K. (2024). A Phase II, Open-Label, Randomized Trial of Durvalumab with Olaparib or Cediranib in Patients with Mismatch Repair—Proficient Colorectal or Pancreatic Cancer. Clin. Colorectal Cancer.

[157] Matrana M.R., Tsai F., Cleary J.M., Satti S., Borazanci E., Estes J., Moser J., Do K.T., Du L., Sharma S. (2020). Phase Ib clinical study of CBP501, cisplatin, and nivolumab administered every three weeks in patients with advanced refractory tumors: Efficacy in dose-escalation and expansion cohorts. J. Clin. Oncol..

[158] Puig-Saus C., Sennino B., Peng S., Wang C.L., Pan Z., Yuen B., Purandare B., An D., Quach B.B., Nguyen D. (2023). Neoantigen-targeted CD8+ T cell responses with PD-1 blockade therapy. Nature.

[159] Gubin M.M., Zhang X., Schuster H., Caron E., Ward J.P., Noguchi T., Ivanova Y., Hundal J., Arthur C.D., Krebber W.J. (2014). Checkpoint blockade cancer immunotherapy targets tumour-specific mutant antigens. Nature.

[160] Bassani-Sternberg M., Bräunlein E., Klar R., Engleitner T., Sinitcyn P., Audehm S., Straub M., Weber J., Slotta-Huspenina J., Specht K. (2016). Direct identification of clinically relevant neoepitopes presented on native human melanoma tissue by mass spectrometry. Nat. Commun..

[161] Martin S.D., Brown S.D., Wick D.A., Nielsen J.S., Kroeger D.R., Twumasi-Boateng K., Holt R.A., Nelson B.H. (2016). Low Mutation Burden in Ovarian Cancer May Limit the Utility of Neoantigen-Targeted Vaccines. PLoS ONE.

[162] Burger M.L., Cruz A.M., Crossland G.E., Gaglia G., Ritch C.C., Blatt S.E., Bhutkar A., Canner D., Kienka T., Tavana S.Z. (2021). Antigen dominance hierarchies shape TCF1+ progenitor CD8 T cell phenotypes in tumors. Cell.

[163] Hailemichael Y., Dai Z., Jaffarzad N., Ye Y., Medina M.A., Huang X.F., Dorta-Estremera S.M., Greeley N.R., Nitti G., Peng W. (2013). Persistent antigen at vaccination sites induces tumor-specific CD8+ T cell sequestration, dysfunction and deletion. Nat. Med..

[164] Krishnamurty A.T., Shyer J.A., Thai M., Gandham V., Buechler M.B., Yang Y.A., Pradhan R.N., Wang A.W., Sanchez P.L., Qu Y. (2022). LRRC15+ myofibroblasts dictate the stromal setpoint to suppress tumour immunity. Nature.

[165] Mizutani Y., Kobayashi H., Iida T., Asai N., Masamune A., Hara A., Esaki N., Ushida K., Mii S., Shiraki Y. (2019). Meflin-Positive Cancer-Associated Fibroblasts Inhibit Pancreatic Carcinogenesis. Cancer Res..

[166] Mi H., Sivagnanam S., Betts C.B., Liudahl S.M., Jaffee E.M., Coussens L.M., Popel A.S. (2022). Quantitative Spatial Profiling of Immune Populations in Pancreatic Ductal Adenocarcinoma Reveals Tumor Microenvironment Heterogeneity and Prognostic Biomarkers. Cancer Res..

[167] Ho W.J., Jaffee E.M. (2021). Macrophage-Targeting by CSF1/1R Blockade in Pancreatic Cancers. Cancer Res..

[168] Vonderheide R.H. (2020). CD40 Agonist Antibodies in Cancer Immunotherapy. Annu. Rev. Med..

[169] Hu Z.I., Shia J., Stadler Z.K., Varghese A.M., Capanu M., Salo-Mullen E., Lowery M.A., Diaz L.A., Mandelker D., Yu K.H. (2018). Evaluating Mismatch Repair Deficiency in Pancreatic Adenocarcinoma: Challenges and Recommendations. Clin. Cancer Res..

[170] Luchini C., Brosens L.A.A., Wood L.D., Chatterjee D., Shin J.I., Sciammarella C., Fiadone G., Malleo G., Salvia R., Kryklyva V. (2021). Comprehensive characterisation of pancreatic ductal adenocarcinoma with microsatellite instability: Histology, molecular pathology and clinical implications. Gut.

[171] Taïeb J., Sayah L., Heinrich K., Kunzmann V., Boileve A., Cirkel G., Lonardi S., Chibaudel B., Turpin A., Beller T. (2023). Efficacy of immune checkpoint inhibitors in microsatellite unstable/mismatch repair-deficient advanced pancreatic adenocarcinoma: An AGEO European Cohort. Eur. J. Cancer.

[172] Chakrabarti S., Bucheit L., Starr J.S., Innis-Shelton R., Shergill A., Dada H., Resta R., Wagner S., Fei N., Kasi P.M. (2022). Detection of microsatellite instability-high (MSI-H) by liquid biopsy predicts robust and durable response to immunotherapy in patients with pancreatic cancer. J. Immunother. Cancer.

[173] Marcus L., Lemery S.J., Keegan P., Pazdur R. (2019). FDA Approval Summary: Pembrolizumab for the Treatment of Microsatellite Instability-High Solid Tumors. Clin. Cancer Res..

[174] U.S. Food and Drug Administration (2026). KEYTRUDA (Pembrolizumab) Injection, for Intravenous Use: Highlights of Prescribing Information (BLA 125514, Supplement 190).

[175] Lin D.I., Quintanilha J.C.F., Danziger N., Lang L., Levitan D., Hayne C., Hiemenz M.C., Smith D.L., Albacker L.A., Leibowitz J. (2024). Pan-tumor validation of a NGS fraction-based MSI analysis as a predictor of response to Pembrolizumab. npj Precis. Oncol..

[176] U.S. Food and Drug Administration (2022). VENTANA MMR RxDx Panel: Premarket Approval P210001, Supplement S001.

[177] Coston T., Desai A., Babiker H., Sonbol M.B., Chakrabarti S., Mahipal A., McWilliams R., Ma W.W., Bekaii-Saab T.S., Stauffer J. (2023). Efficacy of Immune Checkpoint Inhibition and Cytotoxic Chemotherapy in Mismatch Repair-Deficient and Microsatellite Instability-High Pancreatic Cancer: Mayo Clinic Experience. JCO Precis. Oncol..

[178] Pahl H.L., Lassmann S., Schultheis A.M., Rau S., Börries M., Duyster J., Zaiss M., Quante M., Becker H., MTB-FR Network (2025). Response to PD-1 inhibition in MMRd/MSS pancreatic ductal adenocarcinoma: The relevance of parallel testing. J. Cancer Res. Clin. Oncol..

[179] Marcus L., Fashoyin-Aje L.A., Donoghue M., Yuan M., Rodriguez L., Gallagher P.S., Philip R., Ghosh S., Theoret M.R., Beaver J.A. (2021). FDA Approval Summary: Pembrolizumab for the Treatment of Tumor Mutational Burden–High Solid Tumors. Clin. Cancer Res..

[180] Marabelle A., Fakih M., Lopez J., Shah M., Shapira-Frommer R., Nakagawa K., Chung H.C., Kindler H.L., Lopez-Martin J.A., Miller W.H. (2020). Association of tumour mutational burden with outcomes in patients with advanced solid tumours treated with pembrolizumab: Prospective biomarker analysis of the multicohort, open-label, phase 2 KEYNOTE-158 study. Lancet Oncol..

[181] Lawlor R.T., Mattiolo P., Mafficini A., Hong S.M., Piredda M.L., Taormina S.V., Malleo G., Marchegiani G., Pea A., Salvia R. (2021). Tumor Mutational Burden as a Potential Biomarker for Immunotherapy in Pancreatic Cancer: Systematic Review and Still-Open Questions. Cancers.

[182] Karamitopoulou E., Andreou A., Wenning A.S., Gloor B., Perren A. (2022). High tumor mutational burden (TMB) identifies a microsatellite stable pancreatic cancer subset with prolonged survival and strong anti-tumor immunity. Eur. J. Cancer.

[183] Quintanilha J.C.F., Storandt M.H., Graf R.P., Li G., Keller R., Lin D.I., Ross J.S., Huang R.S.P., Schrock A.B., Oxnard G.R. (2023). Tumor Mutational Burden in Real-World Patients with Pancreatic Cancer: Genomic Alterations and Predictive Value for Immune Checkpoint Inhibitor Effectiveness. JCO Precis. Oncol..

[184] Lei M., Gai J., McPhaul T.J., Luo H., Lin P., Liu D., Pishvaian M., Roberts N.J., Wu K., He J. (2025). Homologous recombination-DNA damage response defects increase TMB and neoantigen load, but not effector T cell density and clonal diversity in pancreatic cancer. Exp. Hematol. Oncol..

[185] Golesworthy B., Wang Y., Tanti A., Pacis A., Romero J.M., Cuggia A., Domecq C., Bourdel G., Denroche R.E., Jang G.H. (2022). Intra-Tumoral CD8+ T-Cell Infiltration and PD-L1 Positivity in Homologous Recombination Deficient Pancreatic Ductal Adenocarcinoma. Front. Oncol..

[186] Gao Z., Azar J., Zhu H., Williams-Perez S., Kang S.W., Marginean C., Rubinstein M.P., Makawita S., Lee H.S., Camp E.R. (2024). Translational and oncologic significance of tertiary lymphoid structures in pancreatic adenocarcinoma. Front. Immunol..

[187] Croft W., Pearce H., Margielewska-Davies S., Lim L., Nicol S.M., Zayou F., Blakeway D., Marcon F., Powell-Brett S., Mahon B. (2023). Spatial determination and prognostic impact of the fibroblast transcriptome in pancreatic ductal adenocarcinoma. Elife.

[188] Christensen T.D., Maag E., Theile S., Madsen K., Lindgaard S.C., Hasselby J.P., Nielsen D.L., Johansen J.S., Chen I.M. (2024). Circulating immune-related proteins associated with immune checkpoint inhibitor efficacy in patients with pancreatic ductal adenocarcinoma. ESMO Open.

[189] Helmink B.A., Reddy S.M., Gao J., Zhang S., Basar R., Thakur R., Yizhak K., Sade-Feldman M., Blando J., Han G. (2020). B cells and tertiary lymphoid structures promote immunotherapy response. Nature.

[190] Gunderson A.J., Rajamanickam V., Bui C., Bernard B., Pucilowska J., Ballesteros-Merino C., Schmidt M., McCarty K., Philips M., Piening B. (2021). Germinal center reactions in tertiary lymphoid structures associate with neoantigen burden, humoral immunity and long-term survivorship in pancreatic cancer. Oncoimmunology.

[191] Rogers S., Charles A., Thomas R.M. (2023). The Prospect of Harnessing the Microbiome to Improve Immunotherapeutic Response in Pancreatic Cancer. Cancers.

[192] Hayashi M., Ikenaga N., Nakata K., Luo H., Zhong P., Date S., Oyama K., Higashijima N., Kubo A., Iwamoto C. (2023). Intratumor Fusobacterium nucleatum promotes the progression of pancreatic cancer via the CXCL1-CXCR2 axis. Cancer Sci..

[193] Riquelme E., Zhang Y., Zhang L., Montiel M., Zoltan M., Dong W., Quesada P., Sahin I., Chandra V., San Lucas A. (2019). Tumor Microbiome Diversity and Composition Influence Pancreatic Cancer Outcomes. Cell.

[194] Wu M., Li G., Li Y., Chen K., Xu M., Li D., Xu C., Shen M., Li W., Cao J. (2025). Multi-omics analyses inform mechanisms of immunotherapy response in pancreatic cancer. Front. Immunol..

[195] Dopico P.J., Le M.C.N., Burgess B., Yang Z., Zhao Y., Wang Y., George T.J., Fan Z.H. (2022). Longitudinal Study of Circulating Biomarkers in Patients with Resectable Pancreatic Ductal Adenocarcinoma. Biosensors.

[196] Facciorusso A., Arvanitakis M., Crinò S.F., Fabbri C., Fornelli A., Leeds J., Archibugi L., Carrara S., Dhar J., Gkolfakis P. (2025). Endoscopic ultrasound-guided tissue sampling: European Society of Gastrointestinal Endoscopy (ESGE) Technical and Technology Review. Endoscopy.

[197] Mangiavillano B., Crinò S.F., Facciorusso A., Di Matteo F., Barbera C., Larghi A., Rizzatti G., Carrara S., Spadaccini M., Auriemma F. (2023). Endoscopic ultrasound-guided fine-needle biopsy with or without macroscopic on-site evaluation: A randomized controlled noninferiority trial. Endoscopy.

[198] Pishvaian M.J., Blais E.M., Brody J.R., Lyons E., DeArbeloa P., Hendifar A., Mikhail S., Chung V., Sahai V., Sohal D.P.S. (2020). Overall survival in patients with pancreatic cancer receiving matched therapies following molecular profiling: A retrospective analysis of the Know Your Tumor registry trial. Lancet Oncol..

[199] Pishvaian M.J., Bender R.J., Halverson D., Rahib L., Hendifar A.E., Mikhail S., Chung V., Picozzi V.J., Sohal D., Blais E.M. (2018). Molecular Profiling of Patients with Pancreatic Cancer: Initial Results from the Know Your Tumor Initiative. Clin. Cancer Res..

[200] Elhanafi S., Mahmud N., Vergara N., Kochman M.L., Das K.K., Ginsberg G.G., Rajala M., Chandrasekhara V. (2019). Comparison of endoscopic ultrasound tissue acquisition methods for genomic analysis of pancreatic cancer. J. Gastroenterol. Hepatol..

[201] Mohamed W.T., Jahagirdar V., Jaber F., Ahmed M.K., Fatima I., Bierman T., Fu Z., Jones P.G., Hassan A.F., Faber E. (2024). Endoscopic Ultrasound-Guided Fine-Needle Biopsy Versus Aspiration for Tissue Sampling Adequacy for Molecular Testing in Pancreatic Ductal Adenocarcinoma. Cancers.

[202] Aung K.L., Fischer S.E., Denroche R.E., Jang G.H., Dodd A., Creighton S., Southwood B., Liang S.B., Chadwick D., Zhang A. (2018). Genomics-Driven Precision Medicine for Advanced Pancreatic Cancer: Early Results from the COMPASS Trial. Clin. Cancer Res..

[203] Botrus G., Uson Junior P.L.S., Raman P., Kaufman A.E., Kosiorek H., Yin J., Fu Y., Majeed U., Sonbol M.B., Ahn D.H. (2022). Circulating Cell-Free Tumor DNA in Advanced Pancreatic Adenocarcinoma Identifies Patients with Worse Overall Survival. Front. Oncol..

[204] Bettegowda C., Sausen M., Leary R.J., Kinde I., Wang Y., Agrawal N., Bartlett B.R., Wang H., Luber B., Alani R.M. (2014). Detection of Circulating Tumor DNA in Early- and Late-Stage Human Malignancies. Sci. Transl. Med..

[205] Cohen J.D., Javed A.A., Thoburn C., Wong F., Tie J., Gibbs P., Schmidt C.M., Yip-Schneider M.T., Allen P.J., Schattner M. (2017). Combined circulating tumor DNA and protein biomarker-based liquid biopsy for the earlier detection of pancreatic cancers. Proc. Natl. Acad. Sci. USA.

[206] Heumann T., Judkins C., Li K., Lim S.J., Hoare J., Parkinson R., Cao H., Zhang T., Gai J., Celiker B. (2023). A platform trial of neoadjuvant and adjuvant antitumor vaccination alone or in combination with PD-1 antagonist and CD137 agonist antibodies in patients with resectable pancreatic adenocarcinoma. Nat. Commun..

[207] Aaronson N.K., Ahmedzai S., Bergman B., Bullinger M., Cull A., Duez N.J., Filiberti A., Flechtner H., Fleishman S.B., de Haes J.C.J.M. (1993). The European Organization for Research and Treatment of Cancer QLQ-C30: A Quality-of-Life Instrument for Use in International Clinical Trials in Oncology. J. Natl. Cancer Inst..

[208] Fitzsimmons D., Johnson C.D., George S., Payne S., Sandberg A.A., Bassi C., Beger H.G., Birk D., Büchler M.W., Dervenis C. (1999). Development of a disease specific quality of life (QoL) questionnaire module to supplement the EORTC core cancer QoL questionnaire, the QLQ-C30 in patients with pancreatic cancer. Eur. J. Cancer.

[209] Reni M., Braverman J., Hendifar A., Li C.P., Macarulla T., Oh D.Y., Riess H., Tempero M., Lu B., Marcus J. (2021). Evaluation of Minimal Important Difference and Responder Definition in the EORTC QLQ-PAN26 Module for Assessing Health-Related Quality of Life in Patients with Surgically Resected Pancreatic Adenocarcinoma. Ann. Surg. Oncol..

[210] Maharaj A.D., Samoborec S., Evans S.M., Zalcberg J., Neale R.E., Goldstein D., Merrett N., White K., Croagh D., Pilgrim C.H.C. (2020). Patient-reported outcome measures (PROMs) in pancreatic cancer: A systematic review. HPB.

[211] Tu Y., Renfro L.A. (2024). Latest Developments in “Adaptive Enrichment” Clinical Trial Designs in Oncology. Ther. Innov. Regul. Sci..

[212] Liang K.L., Azad N.S. (2025). Immune-Based Strategies for Pancreatic Cancer in the Adjuvant Setting. Cancers.

[213] Wainberg Z.A., Weekes C.D., Furqan M., Kasi P.M., Devoe C.E., Leal A.D., Chung V., Perry J.R., Kheoh T., McNeil L.K. (2025). Lymph node-targeted, mKRAS-specific amphiphile vaccine in pancreatic and colorectal cancer: Phase 1 AMPLIFY-201 trial final results. Nat. Med..

[214] Ullern A., Garborg K.K., Chauhan S.K., Holm K., Bang C., Dunn C., Johnsen P.H., Hov J.E.R., Kyte J.A. (2026). Safety and efficacy of fecal microbiota transplantation in solid cancers resistant to immune checkpoint inhibitors: Results of the MITRIC trial. J. Immunother. Cancer.

[215] Hill C.S., Parkinson R., Jaffee E.M., Sugar E., Zheng L., Onners B., Weiss M.J., Wolfgang C.L., Cameron J.L., Pawlik T.M. (2025). Phase 1 Study of Adjuvant Allogeneic Granulocyte-Macrophage Colony-Stimulating Factor–Transduced Pancreatic Tumor Cell Vaccine, Low-Dose Cyclophosphamide, and Stereotactic Body Radiation Therapy Followed by FOLFIRINOX in High-Risk Resected Pancreatic Ductal Adenocarcinoma. Int. J. Radiat. Oncol. Biol. Phys..

[216] Hahn A., Irenaeus S., Sandin L.C., Nordström C., Wenthe J., Lövgren T., Eriksson E., Alsaqal S., Pahnke S., Sundin A. (2026). Tumor Microenvironment Gene Engineering with LOAd703 in Patients with Solid Malignancies: LOKON002 Phase I/IIb Clinical Trial. Clin. Cancer Res..

[217] Miyazawa M., Katsuda M., Maguchi H., Katanuma A., Ishii H., Ozaka M., Yamao K., Imaoka H., Kawai M., Hirono S. (2017). Phase II clinical trial using novel peptide cocktail vaccine as a postoperative adjuvant treatment for surgically resected pancreatic cancer patients. Int. J. Cancer.

[218] Du J., Lu C., Mao L., Zhu Y., Kong W., Shen S., Tang M., Bao S., Cheng H., Li G. (2023). PD-1 blockade plus chemoradiotherapy as preoperative therapy for patients with BRPC/LAPC: A biomolecular exploratory, phase II trial. Cell Rep. Med..

[219] Suzuki N., Hazama S., Iguchi H., Uesugi K., Tanaka H., Hirakawa K., Aruga A., Hatori T., Ishizaki H., Umeda Y. (2017). Phase II clinical trial of peptide cocktail therapy for patients with advanced pancreatic cancer: VENUS-PC study. Cancer Sci..

[220] Sha H., Tong F., Ni J., Sun Y., Zhu Y., Qi L., Li X., Li W., Yang Y., Gu Q. (2024). First-line penpulimab (an anti-PD1 antibody) and anlotinib (an angiogenesis inhibitor) with nab-paclitaxel/gemcitabine (PAAG) in metastatic pancreatic cancer: A prospective, multicentre, biomolecular exploratory, phase II trial. Signal Transduct. Target. Ther..

[221] Cheng K., Li X., Lv W., Zhao G., Zhou R., Chang C., Yang H., Li R., Li Z., Chen Y. (2024). Spatial interactions of immune cells as potential predictors to efficacy of toripalimab plus chemotherapy in locally advanced or metastatic pancreatic ductal adenocarcinoma: A phase Ib/II trial. Signal Transduct. Target. Ther..

[222] Xue R., Wei M., Yuan J., Li Z., Zhou Y., Xue Z., Wu Y., Han H., Zhou J., Yu X. (2025). A phase 1b/2 study of first-line anti-PD-L1/TGF-βRII fusion protein SHR-1701 combined with nab-paclitaxel and gemcitabine for advanced pancreatic ductal adenocarcinoma. Signal Transduct. Target. Ther..

[223] Wang Q., Tong F., Qiao L., Qi L., Sun Y., Zhu Y., Ni J., Liu J., Kong W., Liu B. (2024). Hypofractionated radiotherapy plus PD-1 antibody and SOX chemotherapy as second-line therapy in metastatic pancreatic cancer: A single-arm, phase II clinical trial. Cancer Immunol. Immunother..

[224] Safyan R.A., White R.A., Gonda T.A., Lee S.M., Han J., Kuriakose N., Yamamoto N.K., Kugel S., Jamison J.K., Manji G.A. (2026). Phase 2 study of azacitidine plus pembrolizumab as second-line treatment in patients with locally advanced or metastatic pancreatic ductal adenocarcinoma. Oncologist.

[225] Johnson M., Dudek A.Z., Sukari A., Call J., Kunk P.R., Lewis K., Gainor J.F., Sarantopoulos J., Lee P., Golden A. (2022). ARRY-382 in Combination with Pembrolizumab in Patients with Advanced Solid Tumors: Results from a Phase 1b/2 Study. Clin. Cancer Res..

[226] Lee V., Sachidanand A.S., Rodriguez C., Wang J., Onners B., Qi H., Parkinson R., McPhaul T., Durham J., Ding D. (2026). The combination of a cancer vaccine, pembrolizumab, and stereotactic body radiation in patients with locally advanced pancreatic cancer: A single-arm, phase II study. Nat. Commun..

[227] Wang J., Gai J., Zhang T., Niu N., Qi H., Thomas D.L., Li K., Xia T., Rodriguez C., Parkinson R. (2024). Neoadjuvant radioimmunotherapy in pancreatic cancer enhances effector T cell infiltration and shortens their distances to tumor cells. Sci. Adv..

[228] Palmer D.H., Valle J.W., Ma Y.T., Faluyi O., Neoptolemos J.P., Jensen Gjertsen T., Iversen B., Amund Eriksen J., Møller A.S., Aksnes A.K. (2020). TG01/GM-CSF and adjuvant gemcitabine in patients with resected RAS-mutant adenocarcinoma of the pancreas (CT TG01-01): A single-arm, phase 1/2 trial. Br. J. Cancer.

[229] Ota S., Miyashita M., Yamagishi Y., Ogasawara M. (2021). Baseline immunity predicts prognosis of pancreatic cancer patients treated with WT1 and/or MUC1 peptide-loaded dendritic cell vaccination and a standard chemotherapy. Hum. Vaccin. Immunother..

