# Supplementary material for: Combination Immunotherapy in Pancreatic Cancer: Current Clinical Evidence and Mechanistic Rationale for Overcoming a “Cold” Tumor
Source: Cancers (Basel). 2026 Sep 17;18(18):3019. doi: 10.3390/cancers18183019 (PMC13605460; doi:10.3390/cancers18183019)
Supplement: Supplementary file 1 [file cancers-18-03019-s001.zip › cancers-4524404-supplementary.pdf]

Supplementary Material

Combination Immunotherapy in Pancreatic Cancer: Current Clinical Evidence and Mechanistic Rationale for Overcoming a "Cold" Tumor

Bode T. Eisenmenger, Abraham S. Rappaport, Liam H. Connell, Victor T. Petruc, Ethan J. Riordan, Mark R. Wakefield and Yujiang Fang

Submitted to *Cancers* (MDPI), manuscript ID: cancers-4524404. Correspondence: yujiang.fang@dmu.edu

S1. Complete PubMed/MEDLINE search strings

The supplied working review described execution of the two PubMed/MEDLINE strings below through NCBI E-utilities, with an English-language restriction and a publication cutoff of 29 August 2026. It reported 3,410 and 4,089 records. The strings are retained exactly as the documented historical queries. The revision did not recover the complete historical retrieval and screening logs; re-execution against a changing index cannot validate those earlier counts or decisions. The source audit through 5 September 2026 is described separately in Sections S3.3 and S4.

S1.1. Structured query as first executed

```
(("pancreatic ductal adenocarcinoma"[tiab] OR "pancreatic ductal adenocarcinomas"[tiab] OR "pancreatic adenocarcinoma"[tiab] OR "pancreatic cancer"[tiab] OR "pancreatic neoplasm*"[tiab] OR "Pancreatic Neoplasms"[Mesh] OR "Carcinoma, Pancreatic Ductal"[Mesh] OR PDAC[tiab])) AND ((immunotherap*[tiab] OR "Immunotherapy"[Mesh] OR "immune checkpoint*"[tiab] OR "checkpoint blockade"[tiab] OR "checkpoint inhibitor*"[tiab] OR "PD-1"[tiab] OR "PD-L1"[tiab] OR "CTLA-4"[tiab] OR "LAG-3"[tiab] OR "TIGIT"[tiab] OR pembrolizumab[tiab] OR nivolumab[tiab] OR durvalumab[tiab] OR tremelimumab[tiab] OR ipilimumab[tiab] OR atezolizumab[tiab] OR "cancer vaccine*"[tiab] OR "Cancer Vaccines"[Mesh] OR GVAX[tiab] OR "CRS-207"[tiab] OR neoantigen[tiab] OR "CD40 agonis*"[tiab] OR sotigalimab[tiab] OR mitazalimab[tiab] OR "oncolytic"[tiab] OR pelareorep[tiab] OR "chimeric antigen receptor"[tiab] OR "CAR-T"[tiab] OR "T-cell receptor"[tiab] OR "bispecific"[tiab] OR "adoptive cell*"[tiab] OR motixafortide[tiab] OR "BL-8040"[tiab] OR defactinib[tiab] OR entinostat[tiab] OR galunisertib[tiab] OR "CXCR4"[tiab] OR "CSF1R"[tiab] OR "CCR2"[tiab] OR "CXCR2"[tiab] OR "STING agonis*"[tiab])) AND (("Clinical Trial"[pt] OR "Randomized Controlled Trial"[pt] OR "Clinical Trials as Topic"[Mesh] OR "phase 1"[tiab] OR "phase I"[tiab] OR "phase 2"[tiab] OR "phase II"[tiab] OR "phase 3"[tiab] OR "phase III"[tiab] OR "single-arm"[tiab] OR "single arm"[tiab] OR trial[tiab] OR "cohort"[tiab] OR patients[tiab])) AND ("humans"[MeSH Terms] OR patients[tiab] OR participants[tiab]) AND english[lang] AND ("1900/01/01"[Date - Publication] : "2026/08/29"[Date - Publication])
```

S1.2. Amended query

```
(("pancreatic ductal adenocarcinoma"[tiab] OR "pancreatic ductal adenocarcinomas"[tiab] OR "pancreatic adenocarcinoma"[tiab] OR "pancreatic cancer"[tiab] OR "pancreatic neoplasm*"[tiab] OR "Pancreatic Neoplasms"[Mesh] OR "Carcinoma, Pancreatic Ductal"[Mesh] OR PDAC[tiab])) AND ((immunotherap*[tiab] OR "Immunotherapy"[Mesh] OR "immune checkpoint*"[tiab] OR "checkpoint blockade"[tiab] OR "checkpoint inhibitor*"[tiab] OR "PD-1"[tiab] OR "PD-L1"[tiab] OR "CTLA-4"[tiab] OR "LAG-3"[tiab] OR "TIGIT"[tiab] OR pembrolizumab[tiab] OR nivolumab[tiab] OR durvalumab[tiab] OR tremelimumab[tiab] OR ipilimumab[tiab] OR atezolizumab[tiab] OR "cancer vaccine*"[tiab] OR "Cancer Vaccines"[Mesh] OR GVAX[tiab] OR "CRS-207"[tiab] OR neoantigen[tiab] OR "CD40 agonis*"[tiab] OR sotigalimab[tiab] OR mitazalimab[tiab] OR "oncolytic"[tiab] OR pelareorep[tiab] OR "chimeric antigen receptor"[tiab] OR "CAR-T"[tiab] OR "T-cell receptor"[tiab] OR "bispecific"[tiab] OR "adoptive cell*"[tiab] OR motixafortide[tiab] OR "BL-8040"[tiab] OR defactinib[tiab] OR entinostat[tiab] OR galunisertib[tiab] OR "CXCR4"[tiab] OR "CSF1R"[tiab] OR "CCR2"[tiab] OR "CXCR2"[tiab] OR "STING agonis*"[tiab] OR "PD1"[tiab] OR "PDL1"[tiab] OR vaccin*[tiab] OR "dendritic cell*"[tiab] OR "natural killer"[tiab] OR "NK cell*"[tiab] OR "cytokine-induced killer"[tiab] OR "gamma delta"[tiab] OR "tumour-infiltrating lymphocyte*"[tiab] OR "tumor-infiltrating lymphocyte*"[tiab] OR "toll-like receptor"[tiab] OR "CD40"[tiab] OR sintilimab[tiab] OR camrelizumab[tiab] OR toripalimab[tiab] OR tislelizumab[tiab] OR penpulimab[tiab] OR serplulimab[tiab] OR spartalizumab[tiab] OR zimberelimab[tiab] OR avelumab[tiab] OR cemiplimab[tiab] OR "SHR-1701"[tiab] OR GV1001[tiab] OR elpamotide[tiab] OR algenpantucel[tiab] OR "IMM-101"[tiab] OR selicrelumab[tiab] OR "CP-870,893"[tiab] OR plerixafor[tiab] OR lenvatinib[tiab] OR anlotinib[tiab] OR surufatinib[tiab] OR azacitidine[tiab] OR "ARRY-382"[tiab] OR "BMS-813160"[tiab] OR LOAd703[tiab] OR "TG01"[tiab] OR "OCV-C01"[tiab] OR "WT1"[tiab] OR "MUC1"[tiab] OR KIF20A[tiab] OR survivin[tiab])) AND (("Clinical Trial"[pt] OR "Randomized Controlled Trial"[pt] OR "Clinical Trials as Topic"[Mesh] OR "phase 1"[tiab] OR "phase I"[tiab] OR "phase 2"[tiab] OR "phase II"[tiab] OR "phase 3"[tiab] OR "phase III"[tiab] OR "single-arm"[tiab] OR "single arm"[tiab] OR trial[tiab] OR "cohort"[tiab] OR patients[tiab])) AND ("humans"[MeSH Terms] OR patients[tiab] OR participants[tiab]) AND english[lang] AND ("1900/01/01"[Date - Publication] : "2026/08/29"[Date - Publication])
```

The amendment adds, to the intervention block only: the unhyphenated spellings PD1 and PDL1; the generic stem vaccin*; dendritic-cell, natural-killer, $\gamma\delta$-T-cell, cytokine-induced killer and tumor-infiltrating lymphocyte terms; Toll-like receptor and CD40 as bare terms rather than as fixed agonist phrases; additional named checkpoint antibodies and fusion proteins (sintilimab, camrelizumab, toripalimab, tislelizumab, penpulimab, serplulimab, spartalizumab, zimberelimab, avelumab, cemiplimab, SHR-1701); and named

agents whose reports do not use a generic immunotherapy term (GV1001, elpamotide, algenpantucel, IMM-101, selicrelumab, CP-870,893, plerixafor, lenvatinib, anlotinib, surufatinib, azacitidine, ARRY-382, BMS-813160, LOAd703, TG01, OCV-C01, WT1, MUC1, KIF20A, survivin). The population and study-design blocks are unchanged.

S2. Study flow

The historical account reported: 3,410 structured-query records; six database-monograph records removed; 3,404 screened; 3,134 excluded at title/abstract stage; 270 assessed further; 214 excluded; and 56 retained. Tables S1 and S2 reproduce its category counts. The six named monograph identifiers were 21834179, 21834181, 21834182, 21882402, 21882406 and 22319803. These figures are arithmetically consistent but do not establish that each historical screening action occurred. Record-level exclusion decisions were unavailable. The reported exclusion of records without abstracts could have missed eligible material; the revision cannot quantify that risk from the retained account.

For the amended query, the historical account reported 4,089 records, 679 additional records, 653 exclusions, 26 further assessments and seven eligible reports. It stated that a per-record breakdown of the 19 later exclusions was not retained. These figures remain as historical reported counts, rather than independently verified revision-screening results.

The historical account attributed 26 further reports to registry, citation and conference searching. The corresponding record list and screening denominator were not supplied. Its independence from the authors' prior reading and its membership within either query stream therefore cannot be established.

The supplied account reconciled its streams as 56 + 7 + 26 = 89 reports, then 89 − 5 + 1 = 85 study entries after related reports and the S2001 conference abstract were considered. Following exclusion of the Zhu trial from the evidence synthesis, the reference list contains 95 entries: 84 row-index reports, five linked analyses and six notices. The retained inventory therefore comprises 84 study/cohort entries. Direct enumeration verifies the inventory and publication counts, not historical screening provenance or independent trial identity. The retained entries include related phases and platform cohorts, as explained in Section S5.6.

S2.1. Reasons for exclusion at title and abstract screening (prespecified query)

Table S1. Historically reported title/abstract exclusion categories; record-level decisions unavailable.

Reason	n
No immunotherapy component	1,487
Not a primary report of an interventional study (review, meta-analysis, editorial, comment, case report)	978
Not a prospective interventional trial report	441
Immunotherapy given as monotherapy, not in combination	72
Population not pancreatic	56
No clinical or translational outcome reported	48
No abstract available for screening	21
Protocol or trial-in-progress report without results	15
Preclinical only	9
Non-PDAC pancreatic histology	7
Total	3,134

S2.2. Reasons for exclusion at full-record eligibility assessment (prespecified query)

Table S2. Historically reported later exclusion categories; record-level decisions unavailable.

Reason	n
Correlative, biomarker or secondary report linked to a study already represented, or otherwise not meeting the tabulation criteria	131
Retrospective, observational or pooled synthesis rather than a prospective interventional trial	47
Published before 2013 and outside the checkpoint-era combination question	15
Preclinical or translational study without a prospective clinical cohort	14
Only immune component is a non-specific cytokine or nutritional immunomodulator	3

Reason	n
Basket or mixed-tumor study without pancreatic-specific efficacy outcomes	3
Fewer than 10 patients with pancreatic cancer enrolled	1
Total	214

S3. Sensitivity of the search, and what the original check did and did not test

The supplied document described known-item and externally referenced checks. Their reported aims can be assessed, but missing item lists limit independent validation.

S3.1. Known-item check (circular by construction)

The historical account described a 35-report known-item set, all retrieved by the final structured query, with an earlier combination-term requirement retrieving 30. The 35 identifiers and contemporaneous results were not supplied. Such a check can test retrieval of known material but cannot estimate sensitivity to unidentified studies; its numerical outcome is not independently verified here.

S3.2. Reported externally referenced check

The documented query amendment broadens intervention terms, including unhyphenated checkpoint names, generic vaccine and cellular terms, and named agents. Those textual changes are verifiable from Section S1. Without the external reference-set list and historical query outputs, they do not establish how many eligible studies were missed through retrieval rather than screening.

S3.3. What the audit found, and where it locates the limitation

The supplied document named seven amendment-reachable reports [S1–S7]. Their historical query membership cannot be validated by arithmetic or a current rerun. In particular, the residual group of 26 reports is not identified individually. It cannot establish that screening was the principal source of loss, the reasons any record was excluded, or the reliability cost of a single assessor. The revision audit checked the 85 supplied entries against obtainable primary reports and registries and identified additional relevant studies. The retained inventory is therefore explicitly nonexhaustive. The CCR2-inhibitor/FOLFIRINOX study and the combination arm of TACTOPS are discussed as additional clinical context in the manuscript, without being silently added to the supplied 85-entry dataset. Smaller or monotherapy reports, including ELI-002 and the single PDAC participant in MITRIC, inform platform or mechanistic context under a separate scope. No revised historical screening denominator, sensitivity estimate or unique-patient total is claimed. Additional qualifying reports outside the preserved matrix include Nishida et al. (2014), a 32-patient WT1–gemcitabine phase I study (DOI 10.1097/CJI.0000000000000020; primary abstract inspected), and Matrana et al. (2020), a CBP501/cisplatin/nivolumab phase Ib conference report (DOI 10.1200/JCO.2020.38.15_suppl.3059; full abstract inspected). The latter describes 13 pancreatic expansion patients and 8 then efficacy-evaluable patients, with mixed-tumor safety reported separately. These studies are retained as additional clinical evidence because the historical matrix is fixed, not because they fail the clarified clinical scope.

S4. Reference and DOI integrity audit

S4.1. Method

For the present revision, both reference lists and their DOI links were extracted from the authoritative working documents. Crossref and PubMed/Europe PMC metadata were compared with the listed identities, with publisher and registry records used to resolve discrepancies where accessible. Linked notices and source versions were checked separately from trial identity. Claim verification used the primary material actually obtained: full reports, targeted sections, primary abstracts, supplements and registries. Metadata retrieval alone was not treated as a full-text scientific review. Final citation, shared-entry and numbering checks were performed after integration.

S4.2. Results

The supplementary list retains 95 entries with separate S numbering. Shared main/supplement entries refer to the same publications. Correction notices are linked to their parent reports and do not constitute additional

clinical studies. The final reference-verification record distinguishes confirmed identity, actual source-access level and remaining notice-content limitations.

Differences caused by online publication versus issue year, markup, consortium placement or surname particles were resolved without treating them as misdirected DOIs. Confirmed corrections include the Adamson author initial in the IMM-101 report and the published title of the niraparib correction. Peer-reviewed online-first status is distinct from a conference abstract.

Reference entries retain the journal style and give up to ten authors before "et al." Additional linked notices were retained or added beside their reports. Bibliographic renumbering does not represent a new clinical result.

Source interpretation follows corrected information where available. The Zhu trial report, its linked notice and its same-cohort secondary analysis have been removed from the bibliographies and evidence synthesis. The defactinib correction changes adverse-event attribution. Other notices address reported dosing, mislabeled panels, units or bibliographic details. A notice's existence does not by itself invalidate all study findings. The contents of the notice linked to the Özdemir stromal-depletion report and the 2020 Know Your Tumor notice could not be fully inspected through the obtained routes; their identities and links are retained without an unsupported claim about their impact.

S5. Eligibility and coding rules

These definitions govern the revision's descriptive appraisal of the retained inventory. They do not demonstrate that the historical search exhaustively implemented its intended eligibility criteria.

S5.1. Inclusion criteria

The supplied tabulation generally selected prospective interventional pancreatic-cancer combinations published from 2013 onward, with at least 10 pancreatic patients and clinical efficacy results. Immune components include checkpoint inhibitors, costimulatory agonists, vaccines, oncolytic viruses, innate agonists, cellular products and selected stromal or myeloid interventions. After the exclusion of one trial, the retained inventory comprises 84 of the 85 supplied study/cohort entries. Additional relevant reports are identified as contextual evidence rather than included in inventory-derived counts. Scope exceptions and analysis populations are specified by entry; preservation of the matrix does not certify uniform eligibility.

S5.2. Exclusion criteria

Protocols without results, retrospective series, pooled syntheses, mixed-cancer studies without separable pancreatic efficacy, and monotherapy platforms fall outside the intended combination tabulation scope. They may inform contextual discussion. Mixed pancreatic histology is identified where present in a clinical report. Linked updates, correlatives and notices are distinguished from new cohorts; related phase and platform entries are retained with explicit overlap rather than treated as independent trials.

S5.3. Evidence-tier descriptor

The four tiers are author-defined design descriptors. They are not validated risk-of-bias scores, evidence-certainty grades or equivalents of Oxford levels. Statistical power, maturity, endpoint success, component attribution and clinical readiness are separate dimensions.

T1: A randomized trial with a prespecified comparative clinical-efficacy hypothesis. This includes comparative phase 2 designs and trials stopped at a planned interim analysis. A missing power detail does not by itself establish an unpowered design. The tier applies to the tested contrast, which may involve several differing components.

T2: Randomized exploratory or regimen-screening studies, studies testing each arm separately against a historical activity benchmark, or studies primarily assessing biological endpoints, safety or feasibility. Randomization may support an exploratory component contrast even when that contrast was not powered for efficacy.

T3: Prospective nonrandomized clinical studies, with or without a concurrent comparison, including historical-benchmark designs. A nonrandomized control does not convert a study to T1 or T2. Mixed platform designs are labeled by the relevant randomized and nonrandomized components.

T4: Preclinical or translational evidence without a clinical efficacy assessment in the relevant population. Clinical monotherapy platform evidence is identified separately rather than being mislabeled as preclinical.

S5.4. Per-study flags

Flags are descriptive and analysis-specific: (a) limitations of a concurrent same-backbone comparison for the claimed component effect; (b) a historical or external comparison; (c) an outcome-derived, post hoc or outcome-selected exploratory analysis; (d) incomplete or selected analysis populations; (e) a specific unresolved reporting discrepancy; (f) conference-abstract or non-peer-reviewed publication; (g) mixed histology or tumor type. Prespecified exploratory measurements do not automatically receive c, nor does an appropriate measurable-disease response population automatically receive d. Peer-reviewed online-first articles do not receive f solely because final pagination is unavailable. Corrected errors and distinct prespecified analysis sets are not automatically unresolved discrepancies. Each row identifies the component or analysis affected. An active immune-containing comparator can isolate an added component; expected all-treated safety and measurable-disease response sets do not automatically receive flag d.

S5.5. Barrier-measurement coding

Measurement descriptions record specimen, timing, assay, evaluable denominator and the component function assessed. Baseline tissue, on-treatment tumor tissue, resection specimens, blood and skin assays answer different questions. An unpaired post-treatment sample is distinguished from a paired analysis; selected-lesion observations are not representative whole-cohort evidence. The categories distinguish a reported measurement, an assay collected or attempted without a reported result, a measurement not reported in an inspected full report, inaccessible or unresolved information, and tissue sampling not applicable to the clinical setting. Blood immunogenicity can be appropriate after complete resection. ctDNA and CA19-9 measure tumor burden rather than directly establishing an immune mechanism. No binary tumor-measurement percentage is derived from these heterogeneous settings.

S5.6. Reporting conventions in Table S5

Population cells distinguish enrolled, randomized, treated and evaluable patients where established. These denominators are not added into a unique-patient total. PRINCE's reported efficacy population includes phase 1b participants; COMBAT reports include related cohorts and an updated chemotherapy cohort; MORPHEUS publications share control patients. The source “85 studies” denotes the original 85 inventory entries, not independent protocols or disjoint patient sets. One trial has been removed from the evidence synthesis, leaving 84 entries in Table S5. Primary-endpoint cells state the actual endpoint and success rule when available. Clinical efficacy, dose-finding, immunogenicity and feasibility outcomes are not interchangeable. Source-reported estimates and intervals are used; no review-derived efficacy interval is presented.

S5.7. Controlled vocabularies applied to evidence claims

Mechanistic plausibility, hypothesis-generating clinical activity and a supported comparative effect are described separately. Regimen-level clinical readiness remains investigational unless the relevant clinical indication is established. A design tier does not automatically confer the term “promising” or demonstrate benefit.

Biomarker descriptions identify treatment selection, trial enrichment, prognosis or pharmacodynamics. Tumor-agnostic dMMR/MSI-H and TMB-high authorization supports treatment selection under the applicable assay and clinical conditions. Evidence for a PDAC-specific treatment interaction, and for choosing an added combination component, is assessed separately. Exploratory immune or molecular associations are not treated as validated selectors.

S6. Corpus cells that are incomplete

Tables S3 and S4 summarize source-access limitations and narrowly unresolved reported details after this revision. Table S3 concerns information not established from obtainable material; Table S4 concerns a specific omission or discrepancy identified in the inspected material. Neither is proof that no other public source supplies the answer. Table S5 gives the context and affected analysis for each entry. Unavailable values are not inferred, and a source limitation is not equated with an absent measurement.

S6.1. Values that could not be established from the report obtained

Table S3. Values in Table S5 that could not be established from the report obtained.

Study or report	Field	Information not established from obtained material
Aoki γδ-cell transfer [S8]	Endpoints, efficacy and immune analyses	The accessed primary abstract does not establish the prespecified success rule, complete between-group survival estimates or assay-specific evaluable populations. Allocation depended on cell expansion; this was not randomized.
CISPD3 [S1]	Immune assays and analysis details	Full-text assay methods, power details and response-population definitions were not obtained. The randomized comparative OS endpoint is established; missing registry correlatives do not prove that no specimens were collected.
Hill adjuvant Cy/GVAX–SBRT [S9]	Immune-assay results	The registry supplies safety and dose-finding definitions, now reflected in Table S5. Immune-assay results were not established from the obtained abstract and registry; lack of a registered immune endpoint does not prove that sampling did not occur.
S-1 ± cytokine-induced killer cells [S2]	Primary endpoint, arm populations and immune assays	The accessed abstract does not establish the prespecified primary endpoint, complete analysis-population definitions or immune-assay content. Arm sizes inferred from percentages are not independently verified counts.
THAG [S3,S10]	Full-cohort endpoint adjudication and tissue assays	The final abstract reports response and resection results, but a formal co-primary success declaration and detailed tumor immune-assay methods were not obtained. Earlier serial ctDNA data are a separate tumor-burden assessment.
TRIPLE-R [S11]	Biopsy analyses	The accessed primary abstract establishes failure of the response threshold but does not establish the full-report biopsy methods or immune results.
daNIS-1 [S12]	Paired-tumor assay denominators	The June 2026 peer-reviewed abstract reports paired tumor RNA changes, but assay-specific populations were not available in the obtained material. This is a full-text access limit, not a demonstrated omission from the complete publication.
Sotigalimab phase 1b; pelareorep–pembrolizumab; nivolumab–gem/nab-P [S13–S15]	Correlative methods and evaluable populations	Primary abstracts support reported biological findings, but detailed paired-tissue and blood-assay populations were not independently established. For pelareorep–pembrolizumab, detailed survival and event-specific safety values also remain access-limited.
CP-870,893 phase 1 [S4]	Exposure and individual-biopsy claim	The relation between 22 reported entrants and 21 treated participants, as well as the individual on-treatment liver-biopsy claim, was not resolved from obtained primary material. These are not established reporting errors.
COMBAT cohorts 1 and 2 [S16,S17]	Immune assays and response definitions	The first abstract supports tumor CD8 changes, but exact paired-assay populations and the later report's full response-set definition were not obtained. Publication/registry response-population differences are specified in Table S4.
TeloVac; algenpantucel-L phase 2 [S18,S19]	Immune-assay populations	Precise blood/skin or antibody-assay populations were not independently confirmed from the obtained primary material. The phase 2 algenpantucel-L report's formal endpoint-success rule also remains unestablished.
Algenpantucel-L phase 3 [S20]	Immune-assay content	No immune result is supplied by the accessed primary abstract; full-report correlative content was not independently inspected. This does not establish absence of an assay.
LOKON002 [S21]	Pancreatic-specific tissue analysis	Registry results supply pancreatic efficacy values. The PDAC-specific paired denominator for the reported week-13 tumor Th1 change remains unverified; the blood population spans histologies.
Niraparib–checkpoint combinations; DAPPER [S22–S24]	On-treatment tumor analyses	A tumor immune result was not verified for the niraparib report. DAPPER reports paired tissue analyses, but the exact PDAC-specific paired population was not established; pooled colorectal/PDAC correlatives are not PDAC-only evidence.
SWOG S2001 [S25]	Response-population detail and immune results	The preliminary 2026 conference report does not establish all response denominators or provide immune pharmacodynamic results. Measurable-disease response populations can differ legitimately from the randomized PFS population.
CheckMate 032 [S26]	Baseline PD-L1 assay populations	The main report refers to supplemental Table 3 for PD-L1-stratified response results; the complete assay population was not established from obtained material. The published response thresholds and power calculations are available and are not missing.

Study or report	Field	Information not established from obtained material
Weiss gem/nab-P–pembrolizumab [S27,S28]	Detailed serious/fatal toxicity	The obtained primary report and linked correction support the revised population and survival figures; detailed serious/fatal-event counts and attribution were not independently verified.

S6.2. Unresolved details in inspected reports

Table S4. Specific unresolved details in inspected reports; limitations apply to the named field.

Study or report	Field	Specific unresolved details in inspected material
VENUS-PC; PEGASUS-PC [S29,S30]	Reported blood immune analyses	VENUS reports peptide-specific ELISpot–survival comparisons, but assay-specific denominators are not explicit in the inspected text. PEGASUS reports no significant CTL-gene increase, with detailed data not shown. Neither statement means that no immune assay was performed.
Tremelimumab–gemcitabine; ipilimumab–gemcitabine; REVOLUTION [S31–S33]	Collected or planned analyses without reported results	Tremelimumab had registered immune analyses without reported results. Ipilimumab blood sampling was insufficient and analyses were not completed. REVOLUTION collected on-treatment biopsies without reporting post-baseline tumor immune results. These are distinct reporting or feasibility limitations, not inferred absence of collection.
OPTIMIZE-1; LOKON001; COMBAT [S16,S17,S34–S36]	Response counts or populations	OPTIMIZE-1's updated narrative and table differ in PR counts; LOKON001's narrative and response table also differ. COMBAT publication response populations differ from registry mITT populations (cohort 1: 29 versus 30; cohort 2: published rates imply 38, while the registry states 39). Table S5 preserves publication-specific values and identifies these narrow discrepancies (e).
Defactinib; MORPHEUS-PDAC [S37–S40]	Participant and event denominators	Defactinib describes a pretreatment withdrawal despite an aggregate treated count; some safety percentages use an unclear denominator. MORPHEUS has arm-specific header/percentage inconsistencies and overlapping controls. Distinct treatment periods and legitimate efficacy/safety populations must be separated before assigning a discrepancy (e).
POLAR [S41]	Response criteria, tissue populations and grade 3 safety	The report and supporting material differ in the response-success criterion; baseline multiplex-IHC populations differ between Methods and Results. The number of grade 3 events in the narrative does not reconcile with Table 3 of the POLAR report. These specific issues (e) do not invalidate every safety observation or clinical endpoint.
KG4/2015 [S7,S42]	Randomized-stratum result and design description	Methods combine 111 randomized eotaxin-high patients with 37 nonrandomized eotaxin-low controls, unlike the abstract's simplified randomization description (e). A separate high-stratum comparison was planned, but its effect estimate was not located in obtained reports and supplements. Corrected eotaxin units are pg/mL.
Neoadjuvant mFOLFIRINOX–nivolumab; BMS-813160; NASCA; selicrelumab [S43–S46]	Specific safety reporting	The nivolumab report's overall severe-event rate conflicts with a table count; BMS-813160 has an anemia count/percentage inconsistency; NASCA differs in treatment-emergent versus treatment-related labeling; selicrelumab's adjuvant safety denominator differs from the number initiating adjuvant therapy. Table S5/S6 retain event categories and the exact affected population (e).

S7. Retained clinical study and cohort inventory

Table S5 retains 84 supplied study/cohort entries with revised population, design, endpoint, efficacy, measurement and limitation descriptions. It is a nonexhaustive inventory within a narrative review, with related phases and shared-control cohorts identified. Additional clinical context is discussed separately in the manuscript. Reference numbers in this table refer to the supplementary list.

Table S5. Retained 84-entry clinical study/cohort inventory. Entries are not independent trials or disjoint patient populations; groups follow the principal combination context.

**Study (registry)**	**Setting; line**	**Regimen vs. comparator**	**n**	**Tier**	**Primary endpoint and success rule**	**Key efficacy outcomes**	**Target mapping and human measurements**	**Flags**
Chemotherapy-based combinations								
PA.7 [S47,S48] (NCT02879318)	Metastatic; 1L	Gem/nab-P + durvalumab/tremelimumab vs. gem/nab-P	180 randomized (119 combination/61 chemotherapy); 11 additional safety run-in patients; randomized safety set 119/58.	T1	Comparative OS: not met.	Final analysis, 81.6 mo: median OS 9.84 vs. 8.76 mo, HR 0.89 (95% CI 0.64–1.22), *P* = 0.46; PFS 5.45 vs. 5.42 mo, HR 0.95 (95% CI 0.69–1.31), *P* = 0.76; ORR at the 2022 primary analysis 30.3% vs. 23.0% (*P* = 0.28)	B4/B1: No on-treatment tumor immune assay reported. Baseline plasma sequencing in 173 and archival tumor whole-genome sequencing in 46, including tissue–plasma concordance analysis. The four-gene alteration rule is exploratory, outcome-derived and not a functional HRD assay.	c: Outcome-derived input selection and model fitting in the same biomarker dataset; no independent predictive validation.
PRINCE [S49] (NCT03214250)	Metastatic; 1L	Sotigalimab and/or nivolumab + gem/nab-P; no chemo-only arm	99 randomized in phase 2; 93 received treatment. Efficacy set 105 (93 phase 2 plus 12 phase 1b); safety set 108 (93 plus 15 phase 1b). The phase 1b row overlaps; there were 129 unique phase 1b/2 entrants and 123 unique treated patients.	T2	Each arm tested 1-year OS against a 35% historical benchmark; met by nivolumab/chemotherapy only. Between-arm efficacy was exploratory.	Nivolumab/chemotherapy, sotigalimab/chemotherapy and triplet: 1-year OS 57.7% (P=0.006), 48.1% (P=0.062) and 41.3%, respectively. Median OS 16.7/11.4/10.1 months; PFS 6.4/7.3/6.7 months; ORR 50%/33%/31%. The triplet did not meet its benchmark; the abstract and body differ on its P value (0.223/0.233).	B1/B3: Tumor and blood immune analyses. Seven-color multiplex immunofluorescence in 14 paired tumor sets (5/3/6 across arms); serial blood and exploratory survival-associated biomarkers. Biomarker P values were not adjusted for multiplicity.	a: No chemotherapy-only control for the overall immune strategy; randomized immune-partner comparisons remain possible. b: Historical OS benchmark. c: Post hoc survival-associated biomarker analyses. d: Six randomized patients untreated; selected phase 1b carryover populations.

Study (registry)	Setting; line	Regimen vs. comparator	n	Tier	Primary endpoint and success rule	Key efficacy outcomes	Target mapping and human measurements	Flags
Sotigalimab phase 1b [S13] (NCT03214250)	Metastatic; 1L	APX005M + gem/nab-P ± nivolumab	30 enrolled and treated; 24 DLT-evaluable patients formed the response set. Twelve overlap the PRINCE efficacy set and 15 overlap its safety set.	T3	Safety, DLTs and RP2D: RP2D established at 0.3 mg/kg.	Responses in 14 of 24 DLT-evaluable patients (58%) pooled across dose cohorts; feasibility established, component effects not separable	B1/B3: Detailed blood and tumor correlative methods were not independently verified from the accessible primary abstract. The linked PRINCE report provides overlapping translational evidence.	a: No concurrent chemotherapy-only control. d: Response analysis restricted to 24 of 30 DLT-evaluable patients.
OPTIMIZE-1 [S34,S35] (NCT04888312)	Metastatic; 1L	Mitazalimab + mFOLFIRINOX; single arm	70 treated: five at 450 μg/kg and 65 at the 900 μg/kg RP2D. The primary efficacy set required at least two cycles (57 patients); an updated sensitivity analysis included all 65 RP2D patients.	T3	Historical-benchmark ORR endpoint met in the prespecified ≥2-cycle efficacy set; no concurrent comparison.	Primary confirmed ORR 23/57 (40.4%; one-sided 90% lower confidence bound ≥32%). Updated ≥2-cycle set: ORR 24/57 (42.1%), median DoR 12.6 months, PFS 7.7 months and OS 14.9 months. All 65 RP2D patients: ORR 24/65 (36.9%; 90% CI 26.9–47.8), PFS 7.7 months and OS 14.0 months.	B3/B1: Three evaluable paired tumor biopsies, all from objective responders; baseline tumor RNA in 21 and genomic profiling in 37/57. Serum cytokines and whole-blood immunophenotyping were also analyzed. These selected exploratory assays do not establish a validated predictor.	a: No concurrent control. b: Historical ORR benchmark. c: Outcome-defined transcriptomic gene/pathway selection and prognostic modeling. d: Eight RP2D patients excluded before two cycles; all-RP2D sensitivity analysis available. e: Updated body reports 24 PR plus one CR, versus 23 PR plus one CR in Table S3; ORR uses the consistent table total.
Weiss et al. [S27,S28] (NCT02331251)	Metastatic; mixed prior therapy overall, with a chemotherapy-naive efficacy subset.	Gem/nab-P + pembrolizumab; single arm	17 treated; 12 chemotherapy-naive patients in the corrected survival analysis and 11 chemotherapy-naive response-evaluable patients; nine with evaluable plasma copy-number changes.	T3	Prespecified complete-response rate >15%: not met.	Three partial responses and disease control in 11/11 response-evaluable chemotherapy-naive patients. Corrected survival analysis in 12 chemotherapy-naive patients: median PFS 9.1 months and OS 15.0 months.	B4: Plasma copy-number instability in nine patients, retrospectively associated with survival; this measures tumor burden rather than immune pharmacodynamics.	a: No concurrent control. b: Historical comparisons. c: Retrospective plasma copy-number instability analysis. d: Selected chemotherapy-naive and response-evaluable populations. The 2019 correction supplies the corrected survival figure.

Study (registry)	Setting; line	Regimen vs. comparator	n	Tier	Primary endpoint and success rule	Key efficacy outcomes	Target mapping and human measurements	Flags
Morizane et al. [S50] (JapicCTI-184230)	Metastatic; 1L	Nivolumab + mFOLFIRINOX; single arm	31 enrolled, treated and included in the full analysis set.	T3	ORR against historical 31.6% null rate: not met.	ORR 32.3% (90% CI 18.7–48.5); median PFS 7.4 months and OS 13.4 months.	B4: Baseline PD-L1 CPS in 30 and TMB in 24; no on-treatment tumor immune assay reported. The exploratory 5 mutations/Mb TMB cutoff differs from the tissue-agnostic clinical threshold.	a: No concurrent control. b: Historical ORR benchmark
Neoadjuvant mFOLFIRINOX + nivolumab [S43] (NCT03970252)	Borderline resectable; neoadjuvant	mFOLFIRINOX + nivolumab; single arm	28 treated; 22 resected. Survival analysis included 26 patients who completed treatment and had available records.	T3	Co-primary postoperative fistula rate and pathological response reported descriptively; a formal success threshold was not established from the accessed report.	Resection in 22/28 (79%); R0 margins in 19/22 (86%); two pathological complete responses. Median OS was 35 months in the selected 26-person survival set and 38 months among the 22 resected patients.	B4: Tumor RNA profiling in six pretreatment and 21 post-treatment samples, including five paired sets; spatial profiling in six samples. External mFOLFIRINOX tissue comparisons were nonrandomized.	a: No concurrent clinical efficacy control. b: External tissue comparators. c: Explicitly post hoc translational and survival analyses. d: Survival restricted to 26/28 patients. e: The narrative overall grade ≥3 rate conflicts with the table neutropenia count.
Pelareorep + pembrolizumab [S14] (NCT02620423)	Metastatic; ≥2L	Pelareorep + pembrolizumab + investigator-choice chemotherapy; single-arm	11 enrolled; ten response-evaluable.	T3	Phase 1b safety endpoint; no comparative efficacy test.	Disease control in 3/10 response-evaluable patients, including one partial response lasting 17.4 months. Detailed median PFS/OS values were not independently verified from the accessible primary abstract.	B1/B2: Tumor viral replication, on-treatment immune changes and new peripheral T-cell clones were reported. The precise paired-IHC denominator was not independently verified from the accessible abstract.	a: No concurrent control. d: Response set excludes one of 11 enrolled patients.

Study (registry)	Setting; line	Regimen vs. comparator	n	Tier	Primary endpoint and success rule	Key efficacy outcomes	Target mapping and human measurements	Flags
ARC-8 [S51] (NCT04104672)	Metastatic; 1L	Quemliclustat + gem/nab-P ± zimberelimab; no backbone-alone arm	90 randomized in the comparison (61 with/29 without zimberelimab). The broader trial treated 138 patients, including dose escalation and expansion; the pooled 100 mg quemliclustat analysis included 122.	T2	Phase 1b safety endpoint; efficacy comparisons were descriptive, without formal hypothesis testing.	Quemliclustat/chemotherapy plus versus without zimberelimab: confirmed ORR 25% (95% CI 15–37) versus 38% (21–58), median PFS 4.9 versus 8.8 months and OS 14.6 versus 19.4 months. The pooled 100 mg quemliclustat cohort had OS 15.7 versus 9.8 months against a propensity-matched external control (HR 0.634, 95% CI 0.471–0.854); this was an observational comparison.	B3/B4: Tumor analyses included 37 paired biopsies, baseline RNA sequencing in 80 patients and spatial T-cell proximity in 150 regions of interest. Regions are not participant counts; NR4A and survival associations were exploratory.	a: No quemliclustat-free comparator; the randomized zimberelimab addition remains interpretable exploratorily. b: External control. c: Post hoc biomarker and external-control analyses.
BMS-813160 [S44] (NCT03184870)	Metastatic; 1L	BMS-813160 + gem/nab-P ± nivolumab vs. gem/nab-P	Part 2: 115 randomized; 102 treated (35 with nivolumab/35 without nivolumab/32 chemotherapy controls), 93 BICR response-evaluable. Nine of 41 randomized controls were untreated. Separate phase 1 dose cohorts included 21 first-line PDAC patients.	T2	Part 2 co-primary efficacy measures: ORR, DoR and 24-week PFS. Safety and tumor pharmacodynamics were secondary; no comparative efficacy-success threshold was specified.	With nivolumab, without nivolumab and chemotherapy alone: BICR ORR 37.1%, 25.7% and 28.1%; median DoR 44.6, 121 and 31 weeks; 24-week PFS 56%, 56% and 50%; 52-week OS 37%, 51% and 29%. Median PFS/OS were not reported. These use treated populations despite differential pretreatment attrition.	B3: Paired-tumor IHC and RNA analyses. CD163-positive macrophage density increased in 76% of BMS-813160-treated patients versus 46% of chemotherapy controls, opposite to the intended density reduction; marker-positive density does not establish suppressive function.	d: Differential pretreatment attrition, particularly 9/41 untreated controls, with treated-population analysis. e: Table 5 contains an unresolved anemia count/percentage inconsistency. The efficacy row is PDAC-specific despite the broader mixed-tumor publication.
daNIS-1 [S12] (NCT04390763)	Metastatic; 1L	NIS793 ± spartalizumab + gem/nab-P vs. gem/nab-P	153 randomized (50 NIS793/spartalizumab/chemotherapy, 51 NIS793/chemotherapy, 52 chemotherapy); 11 additional safety run-in patients. Randomized safety sets: 46/49/45.	T1	Comparative PFS: no improvement established. The primary registry analysis was Bayesian; the displayed Cox HRs are distinct estimates.	Median PFS 3.91 / 5.52 / 4.37 mo; median OS 10.7 / 8.5 / 10.1 mo; ORR 22.0% / 31.4% / 15.4%; Cox HR for PFS vs. control 1.02 and 1.08	B2: The peer-reviewed publication abstract reports paired tumor RNA analysis with TGF-beta pathway and CAF-marker downregulation as well as post-treatment neutrophil-gene increases. Assay denominators were unavailable in the accessed abstract.	None assigned.

Study (registry)	Setting; line	Regimen vs. comparator	n	Tier	Primary endpoint and success rule	Key efficacy outcomes	Target mapping and human measurements	Flags
IMAGE-1 [S52] (NCT01303172)	Locally advanced or metastatic (84% metastatic); 1L	Gemcitabine + IMM-101 (heat-killed Mycobacterium obuense) vs. gemcitabine, open-label, 2:1	110 randomized (75 combination/35 gemcitabine), ITT; safety set 74/35.	T2	Primary safety/tolerability assessment; clinical efficacy comparisons were exploratory.	ITT median OS 6.7 mo (95% CI 5.4–7.5) vs. 5.6 mo (3.2–7.2), HR 0.68 (0.44–1.04), *P* = 0.074; ITT median PFS 4.1 mo (3.3–4.8) vs. 2.4 mo (2.1–4.0), HR 0.58 (0.37–0.91), *P* = 0.016; ORR 10.7% (4.7–19.9) vs. 2.9% (0.1–14.9), *P* = 0.164; disease stabilization 44% (32.5–55.9) vs. 34.3% (19.1–52.2), *P* = 0.334. Pre-defined metastatic subgroup (n=92): OS 7.0 vs. 4.4 mo, HR 0.54 (0.33–0.87), *P* = 0.01; PFS 4.4 vs. 2.3 mo, HR 0.46 (0.28–0.75), *P* = 0.001. Pre-defined locally advanced subgroup (n=18) favored gemcitabine alone: OS 6.7 vs. 9.2 mo, HR 3.81 (1.03–14.05), *P* = 0.032	B1/B3: No tumor or blood immune pharmacodynamic assay reported. Baseline neutrophil-to-lymphocyte ratio entered an exploratory prognostic model; the report described predictive-marker analyses as ongoing.	c: Outcome-based stepwise inclusion in exploratory prognostic Cox models. Disease-extent subgroups were pre-defined; a formal treatment interaction was not reported.
CISPD3 [S1] (NCT03977272)	Metastatic or recurrent; 1L	Sintilimab + mFOLFIRINOX vs. mFOLFIRINOX, single-center, open-label, 1:1	110 randomized (55/55), ITT; safety sets 53/54. The response-evaluable denominators were not established from the accessible abstract.	T1	Comparative OS: not met.	ITT median OS 10.9 vs. 10.8 mo, HR 1.07 (95% CI 0.69–1.68); median PFS 5.9 vs. 5.7 mo, HR 0.93 (0.62–1.40); ORR 50.0% (95% CI 34.6–65.4) vs. 23.9% (11.1–36.7), *P* < 0.05; DCR 84.1% (72.8–95.3) vs. 71.7% (58.2–85.3)	B4: Immune-assay reporting could not be established from the accessible abstract. The absence of a registry correlative endpoint does not establish absence of sampling.	None established in the accessible sources. Full-text power, assay and response-population details remain access-limited.

Study (registry)	Setting; line	Regimen vs. comparator	n	Tier	Primary endpoint and success rule	Key efficacy outcomes	Target mapping and human measurements	Flags
NASCA [S45] (NCT05218889)	Locally advanced or metastatic (≈81% metastatic); 1L	Surufatinib + camrelizumab + nab-paclitaxel/S-1 versus gemcitabine/nab-paclitaxel; both the immune partners and chemotherapy backbone differ.	96 enrolled: six phase Ib and 90 randomized phase II patients (45/45). The phase II ITT and safety sets were 45/45.	T1	Phase II comparative ORR: met. Phase Ib DLT/RP2D endpoint also met.	ORR 51.1% (23/45; 95% CI 35.8–66.3) vs. 24.4% (11/45; 12.9–39.5); rate difference 26.7% (90% CI 9.9–41.5; 95% CI 6.6–43.9); odds ratio 3.2 (95% CI 1.3–8.2), *P* = 0.01. DCR 91.1% (78.8–97.5) vs. 88.9% (75.9–96.3); median DoR 8.1 mo (5.8–13.3) vs. 6.0 mo (2.3–13.0); median PFS 7.9 mo (5.9–9.4) vs. 5.3 mo (3.4–6.4), HR 0.63 (0.40–0.99), *P* = 0.045; median OS 13.0 mo (10.5–16.1) vs. 11.0 mo (8.4–15.3), HR 0.77 (0.47–1.28), *P* = 0.318	B3/B2: Baseline multiplex tumor IHC in 26/45 experimental-arm patients, including macrophage ratio and CD8/PD-1-positive cells. No on-treatment tissue or comparator-arm immune assay was reported.	a: Different chemotherapy backbones and multiple added partners prevent isolation of camrelizumab. c: Outcome-derived CA19-9 ROC thresholds for six-month survival. e: The table and abstract differ in labeling adverse events as treatment-emergent versus treatment-related.
CP-870,893 phase 1 [S4] (NCT00711191)	Advanced, chemotherapy-naïve (90% metastatic); 1L	CP-870,893 (CD40 agonist) + gemcitabine, dose escalation 0.1 and 0.2 mg/kg then MTD expansion; single-arm.	22 patients in the primary abstract. The supplied report identifies 21 CP-870,893-exposed/analysis patients; this exposure distinction was not independently resolved from the accessible abstract.	T3	Phase 1 safety, tolerability and MTD/RP2D; 0.2 mg/kg established.	Four partial responses were reported. Metabolic responses varied within patients; hepatic-lesion metabolic change correlated with survival in a very small exploratory subset. Detailed RECIST, PFS and OS denominators were not independently verified from the accessible abstract.	B1/B3: Serum cytokine changes and peripheral B-cell activation were reported. The individual on-treatment liver-biopsy claim could not be independently verified from the accessed primary text and is not used for the tumor-measurement classification.	a: No concurrent control. The 22-versus-21 exposure distinction is unresolved, not an established internal error.
Nivolumab + gem/nab-P [S15] (NCT02309177)	Locally advanced or metastatic; 1L	Gem/nab-P + nivolumab; single arm (arm B of a multi-tumor phase 1)	50 treated; six DLT-evaluable in dose finding.	T3	Safety co-primary measures: DLTs and grade 3–4 treatment-emergent events/discontinuation. The authors did not support further development of the regimen.	ORR 18% (95% CI 8.6–31.4); median PFS 5.5 months (95% CI 3.25–7.20) and OS 9.9 months (6.74–12.16). More detailed response-duration and disease-control values were not independently verified from the accessible primary abstract.	B4: Peripheral Ki67-positive CD4/CD8 increases and response associations were reported. Precise paired-tumor IHC and blood-subset denominators could not be independently verified from the accessible abstract; tumor sampling must not be classified as absent on that basis.	a: No concurrent control. b: Historical efficacy comparisons. c: Additional biomarker analyses were post hoc; PD-L1 assessment was prespecified. Assay attrition is not automatically clinical efficacy attrition.

Study (registry)	Setting; line	Regimen vs. comparator	n	Tier	Primary endpoint and success rule	Key efficacy outcomes	Target mapping and human measurements	Flags
Tremelimumab + gemcitabine [S31] (NCT00556023)	Metastatic; 1L (chemotherapy-naive)	Gemcitabine + tremelimumab 6/10/15 mg/kg, 3+3 dose escalation; single arm	34 treated (4/8/22 by dose); 28 response-evaluable. The article body describes 38 enrolled, its abstract describes 34 and the registry describes 37; treated and response denominators are distinct.	T3	No DLT at the tested doses; 15 mg/kg was the maximum administered dose.	Median OS 7.4 mo (95% CI 5.8–9.4); by cohort 5.3 (1.2–14.6), 8.0 (2.3–16.9) and 7.5 (6.0–9.5) mo at 6, 10 and 15 mg/kg; 2 partial responses at 8 weeks among 28 response-evaluable patients (abstract reports 2/19, 10.5%, in the 15 mg/kg group); 7 patients with stable disease >10 weeks; PFS not reported.	B1/B4: Pharmacokinetic results only. Lymphocyte-subset and cytokine analyses were registered, but immune results were not reported; this does not establish that samples were never collected.	a: No concurrent control. b: Historical survival comparisons. d: Six of 34 treated patients lacked response evaluation after premature withdrawal. e: Enrollment differs between the article body, abstract and registry.
Ipilimumab + gemcitabine [S32] (NCT01473940)	Advanced/metastatic; 1L and ≥2L (67% ≥1 prior line, 95% stage IV)	Gemcitabine + ipilimumab, 3+3 dose escalation with MTD expansion; single arm	21 enrolled and treated (13 dose escalation/eight MTD expansion); all 21 included in response analysis.	T3	MTD established at gemcitabine 1,000 mg/m² plus ipilimumab 3 mg/kg.	ORR 14% (3/21) with 7 patients achieving stable disease; median duration of response 11 mo for the three responders, longest 19.8 mo; median PFS 2.78 mo (95% CI 1.61–4.83); median OS 6.90 mo (95% CI 2.63–9.57)	B1/B4: Optional peripheral blood sampling for regulatory T cells and other T-cell subsets was attempted but insufficient; the analyses were not completed. No tumor sampling reported.	a: No concurrent control. b: Historical efficacy comparisons.
Neoadjuvant selicrelumab (CITN-11-01) [S46] (NCT02588443)	Resectable; neoadjuvant and adjuvant	Neoadjuvant selicrelumab alone (arm I) or gem/nab-P then selicrelumab (arm II), then adjuvant gem/nab-P + selicrelumab; non-randomized, sequential arms	16 initiated neoadjuvant therapy (11 arm I/five arm II); 15 resected and 13 initiated adjuvant therapy.	T3	Feasibility and safety co-primary endpoints; no formal feasibility-success result established from the accessed report.	Among 15 resected patients, median DFS from surgery was 13.8 months (95% CI 2.9–24.8) and OS from surgery was 23.4 months (18.0–28.8). No ORR endpoint. These are postoperative outcomes, not enrollment-based ITT estimates.	B1/B3: Post-treatment tumor multiplex IHC in 11 patients (eight/three by arm), compared with external cohorts of 104 untreated and 13 chemotherapy/chemoradiation-treated resections. T-cell enrichment occurred in 9/11, 38/104 and 3/13, respectively. No patient contributed a paired pretreatment tumor sample. Blood cytokines, CyTOF and TCR sequencing were also reported.	a: No concurrent clinical control. b: External tissue comparators. d: Survival conditional on resection. e: The adjuvant AE table uses 16 despite 13 initiating adjuvant therapy; counts and treatment period must remain explicit.

Study (registry)	Setting; line	Regimen vs. comparator	n	Tier	Primary endpoint and success rule	Key efficacy outcomes	Target mapping and human measurements	Flags
PAAG [S5] (NCT05493995)	Metastatic; 1L	Penpulimab + anlotinib + gem/nab-P for eight cycles then penpulimab/anlotinib maintenance; single-arm	66 enrolled, treated and analyzed; 70 screened.	T3	ORR co-primary: 50.0%, exceeding the 40% design target derived from a 23% historical rate. DCR was the other co-primary; no separate DCR success threshold was specified.	ORR 50.0% (33/66; 95% CI 37.4–62.6), all partial responses; DCR 95.5% (63/66; 95% CI 87.3–99.1); median PFS 8.8 mo (95% CI 8.1–11.6), 9- and 12-mo PFS 43.7% (31.4–60.7) and 30.8% (19.6–48.4); median OS 13.7 mo (95% CI 12.4–NR), 9- and 12-mo OS 84.0% (74.9–94.3) and 67.1% (54.7–82.3)	B4/B2: Baseline PD-L1 IHC in 46 (ten positive) and tumor NanoString profiling in 18 for response and 15 for PFS associations; no on-treatment tumor or blood immune assay reported.	a: No concurrent control. b: Historical ORR benchmark. c: Outcome-defined transcriptomic groups and survival decision-tree analyses.
Toripalimab + gem/nab-P [S53] (ChiCTR2000032293)	Locally advanced or metastatic; 1L	Toripalimab + gem/nab-P; single arm, Simon two-stage phase Ib/II	72 enrolled, treated and analyzed (18 phase Ib/54 phase II).	T3	OS co-primary not met: Kaplan–Meier 1-year OS 31.9%, versus a 55% design target and 35% historical rate. Safety was the other co-primary.	ORR 33.3% (24/72); DCR 90.3% (65/72); median PFS 5.6 mo (95% CI 4.9–6.8); median OS 8.9 mo (95% CI 7.3–11.0); 1-year OS 31.9%. ORR 56.3% with PD-L1 TPS ≥5% or CPS ≥5 vs. 25.0% below (*P* = 0.03), without a corresponding PFS or OS difference	B4: Paired serum cytokines, with three of 17 analytes reliably detected and no significant change. Tumor assays were baseline only: multiplex imaging (47 patients for the seven-marker panel), spatial analysis in nine, PD-L1 in 56 and sequencing in 65. The 17-protein and seven-marker descriptions concern different panels.	a: No concurrent control; b: historical 1-year OS benchmark; c: outcome-trained LASSO/Boruta biomarker model.
SHR-1701 + gem/nab-P [S54] (NCT04624217)	Locally advanced or metastatic; 1L	SHR-1701 (anti-PD-L1/TGF-βRII fusion protein) + gem/nab-P; single arm, phase 1b/2	56 enrolled, treated and analyzed, including six phase Ib patients continuing at the RP2D.	T3	Phase Ib RP2D established at 30 mg/kg every three weeks; no DLT. Phase II ORR was estimated with a precision-based sample size, without a formal efficacy-success threshold.	ORR 32.1% (18/56; 95% CI 20.3–46.0); DCR 78.6% (95% CI 65.6–88.4); median DoR 5.5 mo (95% CI 4.1–8.3); median PFS 5.6 mo (95% CI 4.3–6.6); median OS 10.3 mo (95% CI 8.8–12.3) with 6-, 9- and 12-mo OS 82.1%, 60.7% and 39.3%. Subgroups: ORR 40.0% (CPS ≥1) vs. 25.7%; 60.0% (CPS ≥10) vs. 26.7%; 66.7% (TPS 1–49%) vs. 25.0%, with median OS 18.8 vs. 9.9 mo	B4/B2: Baseline pSMAD2/3 and CD8 IHC and tumor RNA profiling; no on-treatment tumor immune assay reported. The baseline pSMAD2/3–PFS association does not establish pharmacodynamic TGF-beta inhibition.	a: No concurrent control; c: outcome-defined transcriptomic subgroups and gene selection; baseline associations do not establish target engagement.
Radiotherapy- and local-ablation-based combinations								

Study (registry)	Setting; line	Regimen vs. comparator	n	Tier	Primary endpoint and success rule	Key efficacy outcomes	Target mapping and human measurements	Flags
Katz et al. [S55] (NCT02305186)	Resectable/borderline resectable; neoadjuvant	Chemoradiotherapy ± pembrolizumab	37 randomized and treated (24 pembrolizumab/13 control); 24 resected.	T2	Safety and CD8+ TIL-density co-primary; one DLT; no convincing CD8-density difference	Pancreatectomy 17/24 versus 7/13; median OS 27.8 versus 24.3 months; comparative clinical efficacy not established	B2/B4: resection tissue, T-cell panel 17/7, myeloid panel 15/7 and macrophage panel 12/7; serial blood flow cytometry	e (CD8 difference CI differs between prose and table)
Xie et al. [S56,S57] (NCT02311361)	Metastatic; ≥2L	Durvalumab ± tremelimumab plus SBRT (8 Gy ×1 or 25 Gy in five fractions)	59 enrolled; 58 safety/survival; 39 response-evaluable.	T3	Safety/feasibility primary; no DLT	One confirmed and one unconfirmed PR among 39; confirmed ORR 2.6%; median PFS 2.0 and OS 3.7 months among 58 treated	B2/B4: five paired baseline/day-29 biopsies, all from cohort B2; nonsignificant CD3/CD8 increases; MHC-I positive in assessed samples	a; d (39/58 response set); e (residual response-count inconsistency; survival-curve labels corrected)
Parikh et al. [S58] (NCT03104439)	Metastatic; ≥2L	Radiotherapy + ipilimumab + nivolumab	25 PDAC treated; 17 received radiation; 65 across PDAC and CRC.	T3	Primary ITT disease-control rate reported; PDAC cohort assessed separately	ITT DCR 5/25 (20%), ORR 3/25 (12%); PFS 2.5 and OS 4.2 months. Irradiated subset: DCR 5/17, ORR 3/17; out-of-field lesions assessed	B1/B2/B4: optional serial tumor biopsies; WES 41 specimens from 17 patients across both cancers; paired RNA analyses also pooled	a; d (radiation-treated subset); g (pooled translational cohort)
CheckPAC [S59] (NCT02866383)	Metastatic; refractory	SBRT + nivolumab vs. SBRT + nivolumab/ipilimumab	88 randomized; 84 treated (41 nivolumab/43 nivolumab–ipilimumab)	T2	Clinical-benefit screening criterion met in dual-checkpoint arm; arms tested independently	CBR 17.1% versus 37.2%; PR 1/41 versus 6/43; median PFS 1.7 versus 1.6 months and OS 3.8 months in both arms	B2/B4: baseline/on-treatment tumor IHC; PD-L1 TPS available in 49/42 samples, not necessarily paired. Linked serum-protein study: baseline 78, any on-treatment 76	b (per-arm screening benchmark); d (four noninitiators); randomized contrast concerns ipilimumab addition, not SBRT
TRIPLE-R [S11] (NCT04258150)	Metastatic; refractory	SBRT + ipilimumab + nivolumab + tocilizumab	26 enrolled and treated	T3	ORR expansion criterion not met; no responses against prespecified 15% threshold	SD 5/26 (19%; 95% CI 7–39); PFS 1.6 and OS 5.3 months	Target mapping B3/B2 (author heuristic): Not established from the accessed primary abstract; full-report biopsy details unavailable	a; b (prespecified activity benchmark); full published report access limited
CA209-9KH [S60] (NCT04098432)	Locally advanced; after FOLFIRINOX	Stereotactic radiotherapy followed by nivolumab	15 enrolled from 22 screened; all initiated treatment	T3	Safety/tolerability primary; investigators judged objective met; efficacy descriptive	No confirmed imaging response; PFS 8.1 and OS 12.9 months from study entry after four induction FOLFIRINOX cycles	Target mapping B1/B4 (author heuristic): Baseline tumor PD-L1/MMR IHC in 9/15; serial CA19-9; no established paired on-treatment immune-state assessment	a; selected for nonprogression after induction; longitudinal QoL/biomarker availability differs from clinical n

Study (registry)	Setting; line	Regimen vs. comparator	n	Tier	Primary endpoint and success rule	Key efficacy outcomes	Target mapping and human measurements	Flags
THAG [S3,S10] (ChiCTR2000032955; NCT05634564)	Borderline resectable or locally advanced; neoadjuvant conversion therapy	Tislelizumab + gemcitabine/nab-paclitaxel + hypofractionated radiotherapy	56 full cohort (17 BRPC/39 LAPC); earlier 29 enrolled/25 analyzed overlap	T3	ORR and R0 resection co-primary; final success declaration unavailable in accessed abstract	ORR 51.8%; median PFS 13.2 and OS 21.3 months; 22/56 resected and 20/22 R0	Target mapping B4/B1 (author heuristic): Baseline blood immune/IL-6 associations; serial ctDNA in earlier cohort (86 samples/25 patients); no verified tumor immune-change result	a; b; d (earlier evaluable and resected subsets); final report access limited
Wang et al. [S61] (ChiCTR2100049799)	Metastatic; 2L after failure of first-line chemotherapy	Concurrent hypofractionated radiotherapy + anti-PD-1 antibody + SOX chemotherapy; single arm	25 enrolled; 23 treated and response-assessed	T3	Median PFS primary, descriptive; no prespecified success threshold reported	ORR 7/23 (30.4%); DCR 16/23 (69.6%); PFS 5.48 and OS 6.57 months	Target mapping B4/B1 (author heuristic): Baseline PD-L1 in seven; blood/NK counts and inflammatory ratios before treatment and after two cycles; no tumor immune remodeling result	a; c (cohort-derived optimal inflammatory-marker thresholds); d (23/25 analysis); e (safety and biomarker-direction discrepancies)
Hill et al. adjuvant Cy/GVAX + SBRT [S9] (NCT01595321; institutional protocol J1179; distinct from NCT03161379 and NCT02648282)	High-risk resected PDAC (close/positive margin ≤1 mm in 71%, node-positive in 79%, both in 63%); adjuvant	Adjuvant SBRT with FOLFIRINOX dose cohorts (full dose, then modified); cohort 3 added one pre-SBRT and four post-chemotherapy Cy/GVAX doses, followed by boosters	19 treated in cohorts of 3, 4 and 12; 18 DLT-evaluable (3, 3 and 12); 12 received Cy/GVAX	T3	Primary grade ≥3 Cy/GVAX-related toxicity; dose finding assessed diarrhea, neutropenia and thrombocytopenia over the first two chemotherapy cycles (8 weeks)	From surgery: all 19 had OS 36.2 and DFS 18.2 months; Cy/GVAX cohort OS 61.3 and DFS 24.1 months; descriptive, nonrandomized	Target mapping B1/B3 (author heuristic): Not established from accessed primary abstract	a (sequential, nonrandomized cohorts); registry and publication populations distinguished
Stromal, trafficking and myeloid-directed combinations								
COMBAT cohort 1 [S16] (NCT02826486)	Metastatic; ≥2L	Motixafortide + pembrolizumab	37 enrolled; 29 response-evaluable; distinct from COMBAT chemotherapy cohort	T3	ORR primary; cohort-specific success rule not established in accessed abstract	DCR 10/29 (34.5%): one PR and nine SD; enrolled median OS 3.3 months	B2/B3: primary abstract reports increased tumor CD8 effectors and reduced selected suppressor populations; exact paired denominators unverified	a; d (29/37 response set); e (publication response n=29 versus registry mITT n=30)

Study (registry)	Setting; line	Regimen vs. comparator	n	Tier	Primary endpoint and success rule	Key efficacy outcomes	Target mapping and human measurements	Flags
COMBAT/KEYNOTE-202 [S17] (NCT02826486)	Metastatic; 2L	Motixafortide + pembrolizumab + nanoliposomal irinotecan/5-FU/leucovorin	43 enrolled; publication and registry response populations differ (registry mITT n = 39); evaluable denominator not inferred from percentages	T3	ORR primary; success criterion not established from accessed abstract	ORR 21.1%, confirmed ORR 13.2%, DCR 63.2%; median PFS 3.8 and OS 6.6 months	Target mapping B2/B4 (author heuristic): Immune results not established from accessed abstract; full-report assay status unresolved	a; b; e (registry mITT n=39 versus publication response percentages implying n=38); initial 22-person report overlaps; e (published and registry response-set definitions require reconciliation)
Defactinib triplet [S37,S38] (NCT02546531)	Metastatic; refractory	Defactinib + pembrolizumab ± gemcitabine in escalation; triplet expansion	34 PDAC enrolled within 42 total; 30 PDAC efficacy-evaluable (20 refractory/10 maintenance); exact treated PDAC n unresolved	T3	Safety/RP2D primary; no DLT	Refractory: DCR 80%, PFS 3.6, OS 7.8 months; maintenance: DCR 70%, PFS 5.0, OS 8.3 months from post-chemotherapy study entry	B2/B3/B4: nine paired pretreatment/post-cycle-3 biopsies; reduced pFAK/suppressive-cell measures and increased CD8 infiltration; no established outcome correlation	a; d (efficacy evaluability); e (residual denominator issues); g (mixed-tumor escalation)
Entinostat + nivolumab [S62] (NCT03250273)	Metastatic; ≥2L	Entinostat lead-in then nivolumab; single arm	30 treated; 27 efficacy-evaluable	T3	ORR endpoint not met (three responses; P=.15)	ORR 3/27 (11.1%); median DOR 10.2 months in responders; PFS 1.89 and OS 2.73 months	B3/B4: tumor mIF 26 baseline/21 after entinostat/four on combination; blood CyTOF 28 paired lead-ins and eight paired combinations; four paired combination biopsies	a; c (explicitly post hoc RNA-sequencing analysis); d (three early toxicity discontinuations excluded from efficacy)
Galunisertib + durvalumab [S63] (NCT02734160)	Metastatic; ≥2L	Galunisertib + durvalumab; single arm	42 treated across doses; 32 at RP2D	T3	Safety/DLT primary; RP2D established without DLT	At RP2D: one PR, seven SD; DCR 25%; PFS 1.87 and OS 5.72 months; nine without scans retained in n=32	Target mapping B2/B3 (author heuristic): Baseline tumor PD-L1 25/32, NGS 20 across doses and circulating proteins; no verified on-treatment tumor TGF-β readout	a; clinical response denominator retains unevaluable patients
Galunisertib + gemcitabine [S64] (NCT01373164)	Locally advanced or metastatic; 1L	Galunisertib + gemcitabine vs. placebo + gemcitabine	156 randomized (104/52); 155 treated (103/52); separate phase Ib mixed-tumor cohort	T1	Bayesian OS criterion met: posterior benefit probability .93 versus .85 threshold; weak-borrowing sensitivity .84	Bayesian median OS 8.9 versus 7.1 months; HR 0.79 (95% CrI 0.59–1.09); frequentist HR 0.86 (95% CI 0.60–1.23); no significant PFS difference	B3: serial plasma TGF-β1 reduction >20% in 64/93 versus 30/50; circulating proteins and T-cell counts; no tumor pathway-change assessment	b (prespecified historical borrowing)

Study (registry)	Setting; line	Regimen vs. comparator	n	Tier	Primary endpoint and success rule	Key efficacy outcomes	Target mapping and human measurements	Flags
MORPHEUS-PDAC [S39] (NCT03193190)	Metastatic; 2L and 3L	Atezolizumab + motixafortide, cobimetinib or simlukafusp alfa versus shared mFOLFOX6/GnP controls	104 treated non-PEG second-line participants, including 46 shared controls; reported second-line cohort 183 randomized/170 treated	T2	ORR screening; four non-PEG experimental arms closed for limited activity	ORR: motixafortide 0%, cobimetinib 0%, simlukafusp q2w 7.1%, q3w 0%; mFOLFOX6 8.7%, GnP 0%; q2w responder MSI-H	Target mapping B2 (author heuristic): Responder-focused tumor immune descriptions; specimen timing insufficient to establish on-treatment barrier remodeling	a (replaced chemotherapy backbone); c (responder-selected tumor immune phenotype descriptions); e (arm-specific header/percentage conflicts); controls shared with PEG report
MORPHEUS atezolizumab + PEGPH20 [S40] (NCT03193190)	Metastatic; 2L	Atezolizumab + PEGPH20 vs. chemotherapy	117 randomized (71/46); 108 treated (66/42); controls shared within MORPHEUS	T2	Early response expansion criterion met; final activity insufficient for further development	ORR 4/66 (6.1%) versus 1/42 (2.4%); PFS 1.5 versus 2.3 and OS 7.1 versus 6.8 months; one experimental responder MSI-H	Target mapping B2 (author heuristic): Baseline HA and PD-L1, each n=53; HA ≥50% cutoff prespecified; no reported on-treatment HA depletion	a (different treatment backbones); e (minor HA-subgroup arithmetic); shared controls
Oleclumab ± durvalumab [S65] (NCT03611556)	Metastatic; 1L	Gem/nab-P (A1) vs. oleclumab + gem/nab-P (A2) vs. oleclumab + durvalumab + gem/nab-P (A3)	195 treated: 25 escalation plus 170 randomized expansion (62/38/70)	T1	Primary triplet-versus-GnP ORR comparison not met: 32.9% versus 29.0%, P=.650	Triplet versus GnP: PFS 7.5 versus 6.7 months, HR 0.72 (95% CI 0.47–1.11); OS 12.9 versus 10.8, HR 0.75 (0.50–1.13)	Target mapping B3/B4 (author heuristic): Baseline CD73 stratification and secondary subgroup analysis; exploratory CD8/PD-L1/genomics; baseline ctDNA n=160; no tumor adenosine-suppression readout	matched chemotherapy comparator
Acalabrutinib ± pembrolizumab [S66] (NCT02362048)	Advanced; ≥2L, median 3 prior lines	Acalabrutinib versus acalabrutinib + pembrolizumab	77 randomized (37/40); 73 treated (35/38); 15 later crossed over	T2	Safety primary; no DLT in initial 12; within-arm DCR screening assessed	ORR 0% versus 7.9%; DCR 14.3% versus 21.1%; PFS 1.4 months in both arms; OS 3.6 versus 3.8 months	B3/B4: serial blood MDSC reduction and T-cell activation; baseline tumor RNA n=18; selected responder pretreatment mIF; on-treatment samples lacked tumor	c (exceptional-responder molecular and tissue analyses); d (four noninitiators); e (crossover PR/SD contradiction); randomized pembrolizumab-addition contrast

Study (registry)	Setting; line	Regimen vs. comparator	n	Tier	Primary endpoint and success rule	Key efficacy outcomes	Target mapping and human measurements	Flags
LEAP-005 cohort G [S67] (NCT03797326)	Metastatic; 2L and 3L	Lenvatinib 20 mg/day + pembrolizumab 200 mg every 3 weeks; single-arm cohort within a multicohort study	103 treated in PDAC cohort G	T3	ORR and safety co-primary, descriptive; no hypothesis-based success threshold reported	BICR ORR 8/103 (7.8%; 95% CI 3.4–14.7), DCR 37.9%; DOR 5.8, PFS 2.1 and OS 4.3 months	Target mapping B4/B2 (author heuristic): Baseline PD-L1 CPS in 96/103; MSI status missing in 74/103. WES/RNA immune-signature analyses were in BTC cohort F, not PDAC	a; primary efficacy includes patients without assessments
Neoadjuvant BMS-813160 + nivolumab [S68] (NCT03496662)	Borderline resectable or locally advanced; neoadjuvant	BMS-813160 + nivolumab + GnP; initial small GnP control cohort	40 treated (32 investigational/8 controls); initial 15 randomized, subsequent 25 single-arm expansion	T2/T3	RP2D 300 mg twice daily established without DLT; ORR descriptive co-primary	ITT PR 7/32 (22%) versus 0/8; BRPC: resection 11/17, R0 9/17; LAPC: resection and R0 2/15	B3/B4: 38 tumor specimens from 24 patients (16/8); reduced intratumoral monocytes/macrophages; scRNA four pre/six post samples; paired blood showed no monocyte change	d (response/completer subsets); small initial control cohort; later expansion nonrandomized
Azacitidine + pembrolizumab [S69] (NCT03264404)	Locally advanced or metastatic; 2L	Subcutaneous azacitidine 50 mg/m² days 1–5 of each 28-day cycle with pembrolizumab 200 mg every 3 weeks from week 3; single arm	36 enrolled; 34 azacitidine-treated safety; 31 received both agents	T3	PFS target four months versus two-month null not met	ORR 3/31 (9.7%), including one unconfirmed PR; confirmed responses 2/31; PFS 1.51 and OS 4.83 months	Target mapping B1/B4 (author heuristic): Baseline PD-L1 n=24; baseline immune IHC in ten outcome-selected samples; week-8 biopsy required but paired immune-remodeling results not reported	a; c (outcome-selected baseline immune-IHC samples); d (lead-in exclusion from n=31); e (responder and microscopy reporting)
ARRY-382 + pembrolizumab [S70] (NCT02880371)	Advanced/metastatic; ≥2L (≥1 prior line of systemic therapy)	ARRY-382 + pembrolizumab; dose escalation and separate phase II tumor cohorts	Phase II: 27 pancreatic treated (25 specified PDAC/two unspecified); six additional phase Ib PDAC	T3	Confirmed ORR primary; no formal success threshold established in inspected text; limited activity	Phase II ORR 1/27 (3.7%), DOR 2.4, PFS 1.4 and OS 2.2 months; phase Ib MSI-H responder reported separately	B3: serum CSF1 and nonclassical-monocyte changes pooled across histologies; screening-only tumor sampling	a; g (pooled pharmacodynamics); e (cohort-label/count discrepancies); all 27 retained for ORR
Plerixafor + cemiplimab [S71] (NCT04177810)	Metastatic; ≥2L (90% ≥2 prior lines, 76% prior resection, 62% prior radiation)	AMD3100/plerixafor 80 μg/kg/h by 7-day continuous infusion each 21-day cycle + cemiplimab 350 mg on day 1; single arm	21 treated; 14 paired tumor biopsies	T3	iRECIST ORR criterion of at least two responses among 21 not met	No objective response; DCR 2/21 (9.5%); PFS 1.9 and OS 4.5 months; one nine-month SD	B2/B3: paired tumor IMC and blood assays; increased CD8 density and macrophage-associated spatial changes; human associations distinct from causal mouse experiments	a; small exploratory tissue analyses; increased cell density alone does not identify trafficking mechanism

Study (registry)	Setting; line	Regimen vs. comparator	n	Tier	Primary endpoint and success rule	Key efficacy outcomes	Target mapping and human measurements	Flags
Canakinumab + spartalizumab + gem/nab-P (SR1) [S6] (NCT04581343)	Metastatic; 1L, treatment-naive	Canakinumab (anti-IL-1β) + spartalizumab + gemcitabine/nab-paclitaxel; single arm	10 treated; separate 11-person GnP blood-correlative cohort	T3	RP2/3 dose established; no DLT in first six during 56-day window	Confirmed ORR 2/10 (20%), one additional unconfirmed PR; DCR 8/10; PFS 4.6 and OS 12.9 months	B3/B4: six paired mIF analyses; scRNA four pairs plus four single-timepoint patients. Blood MoMDSCs decreased; tumor CD8:Treg ratio rose in all six pairs	a (no randomized efficacy control); blood comparator is not an efficacy control
Dual and multi-agent checkpoint combinations								
Durvalumab ± tremelimumab [S72] (NCT02558894)	Metastatic; 2L	Durvalumab + tremelimumab vs. durvalumab	65 randomized (32 combination/33 durvalumab); 64 treated and safety-evaluable (32/32).	T2	Per-arm ORR activity criteria: not met; neither arm expanded.	ORR 3.1% (95% CI 0.08–16.2) vs. 0%; both arms stopped without expansion; grade ≥3 TRAE 22% vs. 6%	B4/B1: Baseline tumor PD-L1 in 65 samples; no on-treatment assay reported.	None assigned
CheckMate 032 [S26] (NCT01928394)	Metastatic; ≥2L	Nivolumab; nivolumab + ipilimumab; nivolumab + ipilimumab + cobimetinib	69 treated in nonrandomized cohorts (18 nivolumab/21 nivolumab–ipilimumab/30 triplet).	T3	ORR activity criteria not met: original cohorts stopped after stage 1; triplet investigator ORR 6.7% did not exceed the 10% null. Original design targeted 80% power at a 25% alternative; triplet design targeted 70% power.	No responses with nivolumab or nivolumab/ipilimumab; triplet investigator-assessed ORR 2/30 (6.7%; 95% CI 0.8–22.1), the primary endpoint, and 3/30 (10.0%; 2.1–26.5) by blinded independent central review; median PFS 3.0 mo, OS 6.2 mo	B4/B1: Baseline PD-L1 IHC was exploratory; exact assay populations were not established from obtained main-text material (supplemental Table 3 referenced). No on-treatment immune result was identified in the inspected main report.	a: No concurrent backbone control; nonrandomized cohorts. Investigator and central-review response counts concern different assessment methods, not necessarily a reporting error.
REVOLUTION [S33] (NCT04787991)	Metastatic; 1L	Gem/nab-P + nivolumab/ipilimumab (cohort A) or + hydroxychloroquine/ipilimumab (cohort B); single arm per cohort	30 enrolled and treated: 15 in each nonrandomized cohort.	T3	Primary safety assessment; both cohorts met the prespecified first-stage activity threshold of at least five responses among 15 patients.	Cohort A: ORR 5/15 (33%), median PFS 9.0 months and OS 17.4 months. Cohort B: ORR 6/15 (40%), PFS 7.9 months and OS 15.8 months. Neither expanded: A because of funding and B because of hydroxychloroquine tolerability.	B4/B1: Serial blood flow cytometry and Olink proteomics. On-treatment biopsies were collected, but no post-baseline tumor immune or sequencing result was reported; baseline/archival tumor sequencing was available.	a: No concurrent control.
Vaccine and antigen-directed combinations								

Study (registry)	Setting; line	Regimen vs. comparator	n	Tier	Primary endpoint and success rule	Key efficacy outcomes	Target mapping and human measurements	Flags
TeloVac [S18] (ISRCTN43482138)	Locally advanced or metastatic; 1L	Gemcitabine/capecitabine ± GV1001 telomerase vaccine, sequential or concurrent	1,062 randomized: 358 chemotherapy, 350 sequential vaccine and 354 concurrent vaccine	T1	No: randomized primary OS comparison.	Median OS 7.9, 6.9 and 8.4 months, respectively; sequential HR 1.19 (98.25% CI 0.97–1.48), concurrent HR 1.05 (98.25% CI 0.85–1.29); overall P = 0.11.	Target mapping B1 (author heuristic): Blood and skin immunogenicity in vaccinated subsets; precise assay denominators were not independently confirmed from the accessible primary abstract.	None assigned
Algenpantucel-L phase 3 [S20] (NCT01836432)	Borderline resectable or locally advanced; neoadjuvant	Standard chemotherapy and chemoradiation ± algenpantucel-L	303 randomized: 158 standard treatment and 145 vaccine; safety populations 140 and 142	T1	No: primary OS.	Median OS 14.9 versus 14.3 months (HR 1.02, 95% CI 0.66–1.58; P = 0.98); reported PFS 13.4 versus 12.4 months.	Target mapping B1 (author heuristic): No immune result in the accessible primary abstract; full-report assay content was not independently verified.	e: reported PFS HR/CI/P-value discordance.
Algenpantucel-L phase 2 [S19] (NCT00569387)	Resected; adjuvant	Algenpantucel-L + gemcitabine and 5-FU chemoradiation; single arm	73 registry enrollment; 70 treated in the primary abstract	T3	Primary 12-month DFS reported; a formal success rule was not established from accessible sources.	12-month DFS 62% and OS 86%; median follow-up 21 months.	Target mapping B1 (author heuristic): Reported antibody findings and assay denominators require full-report confirmation.	a: no concurrent component control; b: historical efficacy comparison.
GVAX prime / CRS-207 boost [S73] (NCT01417000)	Metastatic; ≥2L	Cy/GVAX then CRS-207 vs. Cy/GVAX alone	93 randomized; 90 treated (61 prime/boost, 29 GVAX); initial interim analysis 88; per-protocol analysis 66	T1	Yes: prespecified interim randomized OS criterion.	Initial OS 6.0 versus 3.4 months (HR 0.45; P = 0.0057), crossing the interim boundary; updated OS 6.1 versus 3.9 months (HR 0.59; P = 0.02). Selected per-protocol OS 9.7 versus 4.6 months.	Target mapping B1 (author heuristic): Blood ELISpot in 44 HLA-selected patients; no reported tumor assay. Survival associations used selected post-treatment immune subsets.	c: immune analyses stratified by observed survival above/below six months; d: ≥3 dose per protocol and assay selection.
Cy/GVAX → CRS-207 ± nivolumab [S74] (NCT02243371)	Metastatic; 2L	Cy/GVAX then CRS-207 ± nivolumab	96 randomized; 93 treated (51 nivolumab, 42 control)	T1	No: randomized primary OS.	Median OS 5.9 versus 6.1 months (HR 0.86, 95% CI 0.55–1.34; P = 0.49); RECIST responses 1/51 and 1/42.	Target mapping B1/B4 (author heuristic): Paired tumor mIHC in 22 patients; outcome-defined survival subgroups were exploratory.	c: tissue/serum analyses stratified by observed survival above/below six months; d: paired-biopsy selection.

Study (registry)	Setting; line	Regimen vs. comparator	n	Tier	Primary endpoint and success rule	Key efficacy outcomes	Target mapping and human measurements	Flags
ECLIPSE [S75] (NCT02004262)	Metastatic; ≥2L	Cy/GVAX + CRS-207 vs. CRS-207 vs. physician's-choice chemotherapy	303 randomized: 213 primary-cohort and 90 second-line patients; 235 treated overall	T1	No: primary-cohort intention-to-treat OS.	Primary-cohort OS 3.7 months with Cy/GVAX–CRS-207 versus 4.6 months with chemotherapy (HR 1.17, 95% CI 0.84–1.64); CRS-207 alone 5.4 months.	Target mapping B1/B3 (author heuristic): Post hoc baseline neutrophil-to-lymphocyte ratio in 301 patients; no reported on-treatment immune assay.	a: different-regimen comparison; c: post hoc pooled NLR/GRIm analysis; d: differential non-initiation affects treated analyses, not primary ITT OS.
CRS-207 + nivolumab/ipilimumab ± GVAX [S76] (NCT03190265)	Metastatic; ≥2L	CRS-207 + nivolumab + ipilimumab ± GVAX	61 randomized; 57 treated (30 with GVAX, 27 without)	T2	No: each arm tested against a historical response benchmark.	ORR 0/30 versus 2/27; median OS 4.3 versus 3.9 months and PFS 2.2 versus 1.9 months.	Target mapping B1/B3 (author heuristic): RNA/TCR: 40 samples from 32 patients, including 8 paired sets; paired FFPE in 12, with 5 high-quality imaging-mass-cytometry pairs. Selected antigen-specific clones reached tumor alongside myeloid enrichment.	a: no non-ICI control for the whole regimen; d: assay attrition.
GVAX + ipilimumab maintenance [S77] (NCT01896869)	Metastatic; maintenance after FOLFIRINOX	Ipilimumab + GVAX versus continued chemotherapy, including FOLFIRINOX and fluoropyrimidine-based variants	83 randomized; 82 analyzed (40 immunotherapy, 42 chemotherapy); 39 immunotherapy-treated	T1	No: immunotherapy was inferior for primary OS.	Median OS 9.38 versus 14.7 months (HR 1.75; P = 0.019). Continued chemotherapy included FOLFIRINOX and fluoropyrimidine-based variants.	Target mapping B1/B4/B3 (author heuristic): Six paired tumor sets; 16 baseline biopsies. Tissue analyses cannot establish efficacy in the negative randomized comparison.	a: different-regimen comparison; d: biopsy selection.
Le et al. GVAX + ipilimumab [S78] (NCT00836407)	Locally advanced or metastatic; previously treated	Ipilimumab ± GVAX	30 randomized and treated (15 per arm)	T2	Primary safety study; exploratory survival comparison.	Median OS 3.6 months with ipilimumab versus 5.7 months with GVAX–ipilimumab (HR 0.51; P = 0.072); no RECIST responses.	Target mapping B1/B4 (author heuristic): Blood immune assays in 19 HLA-selected patients; 14 received ≥2 treatments. No reported tumor immune assay.	c: immune analyses stratified by observed median survival of 4.3 months; d: treatment and assay selection.
Neoadjuvant GVAX/Cy + nivolumab + SBRT [S79] (NCT03161379)	Borderline resectable; neoadjuvant	Cy/GVAX + nivolumab + SBRT; single arm	31 enrolled before induction; 18 immunotherapy-treated, 14 resected and 13 evaluable for the tumor CD8 endpoint	T3	No: tumor CD8 endpoint versus historical control.	Median OS 20.4 months (95% CI 18.2–not estimable); one pathological complete response among 14 resections.	Target mapping B1/B3/B4 (author heuristic): Post-treatment resection tissue in 13; registry reports mean log-transformed CD8 density 5.4 (SD 0.8). Historical comparison, not paired baseline sampling.	a: no concurrent component control; b: historical tissue comparator; d: induction and surgery selection; f: abstract-level report.

Study (registry)	Setting; line	Regimen vs. comparator	n	Tier	Primary endpoint and success rule	Key efficacy outcomes	Target mapping and human measurements	Flags
Heumann platform trial [S80] (NCT02451982)	Resectable; neoadjuvant and adjuvant	GVAX + cyclophosphamide (A) vs. + nivolumab (B) vs. + nivolumab and urelumab (C)	54 entered: 42 randomized to A/B, 12 nonrandomly assigned C; 46 treated (17/18/11); 40 resected (16/14/10)	T2/T3	T2 for randomized A/B; T3 for nonrandom C. Biological endpoint met for C versus B; clinical efficacy exploratory.	Median DFS 13.9/14.98/33.51 months and OS 23.59/27.01/35.55 months for A/B/C; comparisons were not statistically significant. Chemotherapy differed across sequential cohorts.	Target mapping B1/B4 (author heuristic): Resection CD137 assay in 7/8/8 patients; C had higher CD8/CD137 density than B (P = 0.003). Few baseline samples; this was not a paired fold-change estimate.	b: nonconcurrent C comparison; d: surgery/assay selection.
Autogene cevumeran [S81,S82] (NCT04161755)	Resected; adjuvant	Atezolizumab → autogene cevumeran → mFOLFIRINOX; single arm	34 enrolled; 28 underwent surgery, 19 received atezolizumab, 16 vaccine and 15 mFOLFIRINOX	T3	Safety primary; RFS/OS secondary and immune response exploratory.	Immune responses in 8/16. At 3.2-year follow-up, responder versus nonresponder RFS was not reached versus 13.4 months (HR 0.14, 95% CI 0.03–0.59; P = 0.007). A post-priming landmark analysis also supported the association.	Target mapping B1 (author heuristic): Blood ELISpot/TCR predominated. One post-priming liver lesion contained vaccine clones and rare matched TP53 but no overt malignant histology: putative micrometastatic, single-case evidence, not paired remodeling.	a: no component control; d: manufacturing, treatment and assay selection.
Mutant *KRAS* vaccine + nivolumab/ipilimumab [S83,S84] (NCT04117087)	Resected; adjuvant	Pooled mutant *KRAS* long peptides + nivolumab + ipilimumab; single arm	12 treated; 11 received perioperative chemotherapy	T3	Safety and maximum pooled KRAS IFN-γ response were co-primary.	Pooled responses in 11/12 and tumor-matched responses in 10/12. Overall median DFS 6.35 months and OS 29.59 months; high/low immune groups (9/3) had exploratory DFS 18.8/2.76 months (HR 0.19; P = 0.024).	Target mapping B1/B4 (author heuristic): Blood assays; one recurrent lung biopsy contained 80 vaccine-specific TCR clonotypes, 55% directed at the tumor mutation. This was not paired on-treatment tumor remodeling.	a: no component control.
Pelareorep + carboplatin/paclitaxel [S85] (NCT01280058)	Metastatic; 1L	Pelareorep + carboplatin/paclitaxel vs. carboplatin/paclitaxel, with crossover on progression	76 randomized; 73 evaluable (36 pelareorep, 37 control); 16 control patients later crossed over	T1	No: randomized primary PFS.	Median PFS 4.9 versus 5.2 months and OS 7.3 versus 8.8 months; seven responses in each arm. Crossover patients are not additional entrants.	Target mapping B1 (author heuristic): Paired blood analysis after two cycles in 70 patients; no reported tumor immune assay. Cytokine findings require multiplicity-aware interpretation.	d: sampling after treatment.

Study (registry)	Setting; line	Regimen vs. comparator	n	Tier	Primary endpoint and success rule	Key efficacy outcomes	Target mapping and human measurements	Flags
LOKON001 [S36] (NCT02705196)	Unresectable or metastatic; 1L and ≥2L	LOAd703 oncolytic adenovirus + gem/nab-P; single arm	22 enrolled, 21 treated and 18 activity-evaluable after ≥3 injections; high-dose cohort 14 treated/11 activity-evaluable	T3	Safety primary; prespecified high-dose response rule met in the selected activity population.	ORR 8/18 (44%) activity-evaluable versus 8/21 (38%) treated; median OS 9.3 versus 8.7 months, respectively. Activity-population PFS 5.4 months.	Target mapping B1 (author heuristic): Blood adenovirus ELISpot in 16, antibodies in 20 and flow cytometry in 13, declining to 8; adenovirus reactivity does not establish tumor-specific immunity.	a: no component control; d: ≥3 dose activity selection; e: narrative response-count discrepancy.
WT1 peptide + gemcitabine [S86] (UMIN000005248)	Locally advanced, metastatic or recurrent; 1L	Gemcitabine + WT1 peptide vaccine vs. gemcitabine	91 enrolled; 85 eligible/evaluable (42 vaccine, 43 control)	T1	No: primary OS; under-recruited relative to the planned 150.	One-year OS 35.7% versus 20.9%; OS HR 0.82 (P = 0.363). Secondary PFS HR 0.66 (P = 0.084), meeting the protocol's two-sided 0.10 significance level.	Target mapping B1 (author heuristic): DTH evaluable in 38/42, including 18 positive; tetramer subsets comprised 15 DTH-positive, 14 DTH-negative and 29 controls. Correlative survival analysis selected patients with ≥2 courses.	d: early-progression and correlative selection.
Survivin 2B vaccine [S87] (UMIN000012146)	Advanced; ≥2L	SVN-2B + interferon-β vs. SVN-2B + placebo vs. double placebo	94 randomized (36/38/20); report states 84 treated, while the described FAS contains 83 (30/34/19)	T1	No: randomized primary PFS.	Median OS 102/96.5/111 days (P = 0.4565). Selected post-progression immune subgroups do not establish randomized survival benefit.	Target mapping B1 (author heuristic): Blood ELISpot/tetramer; within-arm ELISpot increase P = 0.0245, between-arm comparison P = 0.0849. Step-2 immune subset included nine selected patients.	d: postrandomization exclusions; e: 84/83 population discrepancy; g: six non-adenocarcinoma cases.
KG4/2015 [S7,S42] (NCT02854072)	Locally advanced or metastatic; first line; randomized eotaxin-high and nonrandom eotaxin-low strata	Gemcitabine/capecitabine + GV1001 and GM-CSF vs. gemcitabine/capecitabine	148 entered: 111 eotaxin-high randomized (75 vaccine, 36 control), plus 37 nonrandom eotaxin-low controls; reported comparison 75 versus 73	T1/T3	Reported pooled OS positive; separate randomized high-stratum effect not established from obtained sources.	Pooled OS 11.3 versus 7.5 months (P = 0.021); TTP 7.3 versus 4.5 months; PFS 7.3 versus 4.6 months. ORR 20/75 versus 20/73. Censoring and pooled controls limit causal interpretation.	Target mapping B1 (author heuristic): Corrected eotaxin threshold 81.02 pg/mL. Blood immune testing in 75 vaccine patients, 35 ever responders; CD8 subsets 21/14. Post hoc protein analysis in 73.	c: survival-defined groups used to select candidate protein markers; e: abstract randomization description conflicts with Methods; corrected eotaxin units.

Study (registry)	Setting; line	Regimen vs. comparator	n	Tier	Primary endpoint and success rule	Key efficacy outcomes	Target mapping and human measurements	Flags
PEGASUS-PC [S30] (UMIN000001664)	Locally advanced or metastatic; 1L	Gemcitabine + elpamotide (VEGFR2 peptide) vs. gemcitabine + placebo, double-blind, 2:1	159 randomized; 153 FAS (100 vaccine, 53 placebo), including 150 adenocarcinoma and three adenosquamous cases	T1	No: randomized primary OS.	Median OS 8.36 versus 8.54 months (reported HR 0.87, 95% CI 0.486–1.557; P = 0.918).	Target mapping B1/B2 (author heuristic): Peripheral CTL-related gene panel on days 8/29 showed no significant increase; no antigen-specific functional or on-treatment tumor assay reported.	c: post hoc severe injection-site reaction survival analysis; g: three adenosquamous cases.
J15237 [S88,S89] (NCT02648282)	Locally advanced; after 4–8 cycles of induction chemotherapy	Cy/GVAX + pembrolizumab + SBRT then continued immunotherapy; single arm	58 enrolled/treated; 54 efficacy-evaluable, 35 explored and 24 resected	T3	No: primary DMFS target.	Median DMFS 9.8 months versus the 13.6-month design target. OS 21.8 months from first immunotherapy and 28.2 months from diagnosis; resected-only OS is selected.	Target mapping B1/B4/B3 (author heuristic): 54 pretreatment and 45 post-treatment tissue samples; survival-linked paired analyses in 42 and spatial analyses in 17. Historical tissue cohorts were separate; exploratory spatial survival associations were not uniformly significant.	a: no component control; b: external comparisons; c: tissue analyses stratified by observed survival above/below two years; d: induction, treatment and surgery selection.
LOKON002 [S21] (NCT03225989)	Advanced/metastatic solid tumors including pancreatic; any line (standard-of-care gemcitabine-based chemotherapy or gemcitabine immune-conditioning)	Intratumoral LOAd703 (up to 8 biweekly injections, 3 dose levels) + gemcitabine-based chemotherapy; single arm	47 consented, 42 assigned and 41 treated across cancers; 29 pancreatic patients treated and 24 pancreatic response-evaluable	T3	Safety/dose-finding; no controlled efficacy inference.	Registry pancreatic response set: 6/24 partial responses and 17/24 clinical benefit; median PFS 4.62 months and OS 7.29 months. First-line abstract percentages use a different subset.	Target mapping B1/B3 (author heuristic): Blood antibody/activation analyses in 39 across histologies; tumor Th1 increase reported at week 13, but PDAC-specific paired denominator was not verified.	a: no component control; d: ≥3 dose/week 9 response selection; f: abstract-level report; g: mixed histologies.

Study (registry)	Setting; line	Regimen vs. comparator	n	Tier	Primary endpoint and success rule	Key efficacy outcomes	Target mapping and human measurements	Flags
VENUS-PC [S29] (UMIN000008082)	Locally advanced or metastatic; 1L, chemotherapy-naïve	Gemcitabine + KIF20A-66/VEGFR1-1084/VEGFR2-169 peptide cocktail in all patients; comparison is HLA-A*24:02-matched vs. unmatched, not vaccine vs. no vaccine	68 enrolled (38 HLA-matched, 30 unmatched); safety 67 and FAS 66	T3	No: nonrandomized comparison between HLA groups.	Median OS 9.0 versus 10.0 months and PFS 4.7 versus 5.2 months; neither differed significantly. All patients received vaccine plus gemcitabine.	Target mapping B1 (author heuristic): Blood peptide-specific CTL analyses; post-treatment response–survival associations cannot establish treatment prediction. HLA status determined comparison groups.	a: no vaccine-free control; g: one other histology and 15 unassessed cases in the FAS.
CT TG01-01 [S90] (NCT02261714)	Resected stage I–II; adjuvant	TG01/GM-CSF + adjuvant gemcitabine, two sequential vaccination schedules (main and modified cohorts); single arm	32 treated: 19 initial-schedule and 13 modified-schedule patients; 30 immune-evaluable	T3	Safety and immune response study; no controlled efficacy endpoint.	Median OS 33.3 months and DFS 16.1 months from surgery; sequential schedule cohorts are not randomized.	Target mapping B1 (author heuristic): Blood/skin immune assays in 30 evaluable patients; baseline KRAS mutation confirmed in 26/32.	a: no component control; b: historical efficacy context; d: immune-evaluable selection.
OCV-C01 adjuvant phase 2 [S91] (UMIN000007991; linked HLA-unmatched control UMIN000020366)	Resected pancreatic ductal carcinoma; adjuvant	OCV-C01 peptide vaccine + adjuvant gemcitabine; prospective nonrandom comparison	63 screened; 30 vaccine-treated FAS/safety, 27 per protocol; linked prospective HLA-unmatched chemotherapy cohort 15	T3	Prespecified two-year DFS endpoint not yet mature at reported 18-month follow-up.	Median DFS 15.8 months; 18-month DFS 34.6% and OS 69%. Linked nonrandom control DFS 12 months (P = 0.504).	Target mapping B1 (author heuristic): Blood CTL analysis in 29 and baseline tumor IHC in 30, including seven KIF20A-positive tumors. FAS immune/expression survival comparisons were not statistically significant; selected per-protocol findings were stronger.	b: historical design benchmark; d: per-protocol selection.
Ota et al. DC vaccine [S92] (UMIN000027279)	Metastatic, unresectable or relapsed PDAC; 1L and ≥2L (27% chemotherapy-naïve, 44% previously treated)	WT1 and/or MUC1 peptide-loaded dendritic cells (~1×10^7 intradermally every 2–4 weeks, ≥7 doses) + OK-432, with concurrent gem/nab-P (n=33) or FOLFIRINOX (n=15); single arm	48 treated: 33 with gemcitabine/nab-paclitaxel and 15 with FOLFIRINOX	T3	Prospective registered single-arm study; no randomized efficacy test.	ORR 7/48; median PFS 8.1 months and OS 15.1 months.	Target mapping B1 (author heuristic): Blood ELISpot before and after treatment; multivariable prognostic measurements were baseline WT1 (n = 32) and MUC1 (n = 47). Baseline tumor IHC was separate; post-treatment immune associations remain exploratory.	a: no component control; c: outcome-associated univariate screening for multivariable prognostic models.
Cellular and adoptive-transfer combinations								

Study (registry)	Setting; line	Regimen vs. comparator	n	Tier	Primary endpoint and success rule	Key efficacy outcomes	Target mapping and human measurements	Flags
IRE ± allogeneic Vγ9Vδ2 T cells [S93] (NCT03180437)	Locally advanced; after or instead of induction chemotherapy	Irreversible electroporation + one to three courses of allogeneic Vγ9Vδ2 T-cell infusion (group A) vs. irreversible electroporation alone (group B)	62 randomized (30 combination/32 IRE); all included in reported clinical analyses	T1	OS/PFS co-primary; median differences reported with incomplete primary-comparison effect precision. Prospective protocol detail remains unverified.	From treatment: OS 14.5 versus 11 and PFS 11 versus 8.5 months; from diagnosis: OS 22.5 versus 19 and PFS 18.5 versus 15.5 months	B4: peripheral immune-cell, receptor and cytokine assays at baseline/day 90; figure legends n=62; no tumor immune assay	e (AE percentages/counts and survival-model reporting)
S-1 ± cytokine-induced killer cells [S2] (no registration reported)	Advanced; 2L after gemcitabine-based therapy	S-1 + cytokine-induced killer cells versus S-1	58 randomized; arm-specific n unavailable in accessed abstract	T1/T2	Exact tier unresolved: primary endpoint and success rule not established from accessed abstract.	DCR 53.6% versus 40.0%, P=.621; TTP 2.9 versus 2.5 months, P=.037; OS 6.6 versus 6.1 months, P=.09	Target mapping B1/B4 (author heuristic): Not established from accessed primary abstract	No specific flag established from abstract; full-report access limited
Tan et al. NK cell therapy [S94] (ChiCTR1900021764)	Advanced pancreatic cancer (locally advanced 12%, metastatic 88%); 1L	Allogeneic NK cells (1×10^9 to 8×10^9 per infusion, four dose levels) + gemcitabine and S-1; single arm, dose escalation	25 enrolled; 24 NK-infused; 19 efficacy/survival-evaluable, including 16 metastatic	T3	Safety/tolerability primary; no NK DLT or GVHD; MTD not reached	Among 19: ORR 31.6%, DCR 73.7%; PFS 6.6 and OS 10.8 months; survival from enrollment in selected efficacy set	B4: 19 longitudinal PBMC specimens from seven selected patients; TCR sequencing in three responders; no tumor assay	a; c (responder-selected TCR repertoire analysis); d (25/24/19 sets); e (six-month OS CI excludes point estimate)
Aoki et al. γδ T-cell transfer [S8] (UMIN000000931)	Curatively resected pancreatic cancer; adjuvant	Gemcitabine + autologous γδ T cells versus gemcitabine; nonrandomized manufacturing-based allocation	56 recruited; 28 selected for cellular therapy and 20 chemotherapy controls analyzed	T3	Primary endpoint/success rule unavailable in accessed abstract	No significant RFS or OS difference; exact survival estimates unavailable in accessed abstract	B4: peripheral γδ-cell proportions and product purity; post-infusion outcome associations; no verified tumor assay	d (manufacturing/analysis selection); nonrandomized control
PARP-inhibitor and maintenance combinations								

Study (registry)	Setting; line	Regimen vs. comparator	n	Tier	Primary endpoint and success rule	Key efficacy outcomes	Target mapping and human measurements	Flags
Niraparib + nivolumab or ipilimumab [S22,S23] (NCT03404960)	Platinum-sensitive advanced; maintenance	Niraparib + nivolumab versus niraparib + ipilimumab; randomized ICI partner, no PARP-alone control	91 randomized/treated (46 nivolumab, 45 ipilimumab); per-protocol efficacy 84 (44/40)	T2	Ipilimumab arm met its independent PFS6 benchmark per protocol; its ITT interval did not exclude the null. Nivolumab did not meet the benchmark.	Per-protocol PFS6: 20.6% with nivolumab and 59.6% with ipilimumab; ipilimumab ITT PFS6 56.8% (95% CI 41.7–71.9%), versus null 44%. No formal between-arm efficacy test.	Target mapping B1/B4 (author heuristic): No verified on-treatment tumor immune result; platinum sensitivity is eligibility selection, not proof of a predictive immune biomarker.	a: no PARP-alone control; b: independent historical PFS6 benchmark; d: seven efficacy exclusions; g: mixed pancreatic histologies.
POLAR [S41] (NCT04666740)	Advanced, platinum-controlled; maintenance	Pembrolizumab + olaparib, three biomarker cohorts; no control	63 treated: cohorts A/B/C 33/15/15; 59 adenocarcinoma overall; cohort A measurable response set 20	T3	No: reported co-primary response and PFS6 endpoints in cohort A.	Cohort A ORR 7/20 (35%) and PFS6 64%; planned final success counts were ≥12 responses or ≥23 progression-free patients among 33. PFS A/B/C 8.3/4.8/3.3 months; OS 28/18/10 months.	Target mapping B4/B1 (author heuristic): Baseline tissue mIF described as 33 in Results versus 21 in Methods; WES 35 and TIL analysis 38. Serial ctDNA is a tumor-burden measure. Exploratory HRD/immune associations are not validated predictors.	a: no PARP-alone control; b: historical activity benchmarks; d: selected assay subsets; e: response-criterion, assay-n and grade 3 safety discrepancies; g: mixed histologies.
SWOG S2001 [S25] (NCT04548752)	Germline *BRCA1/2*, platinum-controlled; maintenance	Olaparib + pembrolizumab vs. olaparib	73 enrolled; 68 eligible randomized (34 per arm)	T1	No: primary PFS; closed for futility at planned interim analysis.	Median PFS 8.2 versus 6.4 months ($P = 0.82$); OS 20 versus 22.1 months, immature. ORR 28.1% versus 14.3% in response-assessable subsets; no proven added pembrolizumab benefit.	Target mapping B4 (author heuristic): Banked biospecimens; no reported immune pharmacodynamic result in the 2026 abstract.	f: preliminary conference report and immature OS.
CBP501 + cisplatin + nivolumab [S95] (NCT04953962)	Metastatic; ≥3L	CBP501 25 or 16 mg/m² + cisplatin + nivolumab; CBP501 25 mg/m² + cisplatin; or cisplatin + nivolumab	36 randomized (nine per arm); 33 treated (8/9/8/8), 32 imaging-evaluable	T2	Both triplets met individual early PFS3 activity rules; not a powered between-arm comparison.	For high-dose triplet, low-dose triplet, CBP501–cisplatin and cisplatin–nivolumab: PFS3 44.4/44.4/11.1/33.3%; median PFS 2.4/2.1/1.5/1.5 months and OS 6.3/5.3/3.7/4.9 months. ORR 2/9 in the high-dose triplet and zero elsewhere.	Target mapping B3/B4 (author heuristic): No reported on-treatment immune assay; imaging-evaluable attrition does not change the randomized nine-per-arm PFS3 denominator.	a: no standard-care control for whole regimen; e: inconsistent intervals for identical counts in the accessed pre-proof.

Study (registry)	Setting; line	Regimen vs. comparator	n	Tier	Primary endpoint and success rule	Key efficacy outcomes	Target mapping and human measurements	Flags
DAPPER [S24] (NCT03851614)	Advanced; ≥2L, mismatch repair-proficient	Durvalumab + olaparib vs. durvalumab + cediranib; no backbone-alone arm	19 PDAC patients randomized (9 olaparib, 10 cediranib), 18 response-evaluable; broader report included 31 colorectal patients	T2	No significant intratumoral T-cell increase; clinical activity limited.	No confirmed PDAC response; one unconfirmed partial response with cediranib. Median PFS 1.3 versus 2.1 months; pooled PDAC OS 2.7 months.	Target mapping B4 (author heuristic): Baseline/week-4 tissue IHC/RNA showed no significant CD3/CD8 increase. Exact PDAC paired denominator not established from obtained text; survival correlates pooled colorectal and PDAC patients.	a: no durvalumab-alone control; d: paired-assay selection; g: mixed-histology safety/correlative populations.

Table S5 notes: Tiers and analysis-specific flags are defined in Section S5. Measurement descriptions retain specimen, timing, evaluable population and source-access limitations. No cross-study binary measurement percentage or unique-patient total is calculated. 1L, first line; 2L, second line; BICR, blinded independent central review; DCR, disease-control rate; DoR, duration of response; ITT, intention to treat; mFOLFIRINOX, modified FOLFIRINOX; NA, not available; NR, not reached; TRAE, treatment-related adverse event.

S8. Per-trial safety matrix

Table S6 retains 50 selected safety entries from the supplied inventory and revises their denominators, attribution, exposure and comparisons. Its size does not imply that the remaining studies had no safety information. Access limits and unresolved event-specific details are stated alongside the values that can be supported. Safety and efficacy populations may differ legitimately. Within-trial comparisons require the relevant attribution category; rates across rows are not ranked.

Table S6. Selected report-specific safety entries, with denominators, attribution, exposure and treatment contrasts. Unresolved details are qualified without discarding unaffected findings.

Regimen (trial)	Safety denominator	Attribution rule as reported	Exposure context	Comparator and component contrast	Reported severe, serious and fatal events
Gem/nab-P + durvalumab/tremelimumab (PA.7) [S47]	119 vs. 58 (61 randomized to chemotherapy alone, 58 treated; the adverse-event table uses the treated denominator)	All-cause adverse events; investigator-attributed immune-related events described separately	Until progression; first line	Randomized same GnP backbone; estimates the joint addition of durvalumab/tremelimumab	Grade ≥3 AEs: 100/119 (84%) versus 44/58 (76%). Three grade 4 and two grade 5 events in the experimental arm were at least possibly immunotherapy-related. Two grade 5 events in the chemotherapy arm were at least possibly treatment-related.
Sotigalimab and/or nivolumab + chemotherapy (PRINCE) [S49]	108 safety-evaluable, including phase Ib participants; not the 105-person efficacy set	Treatment-related	Until progression; first line	Randomized immune-partner arms on GnP; no chemotherapy-only arm; phase Ib participants also included	Any-grade TRAEs in 98%; CRS 0%, 24% and 34% with nivolumab, sotigalimab and both, respectively. Five grade 3 CRS events and no grade 4–5 CRS; all-drug discontinuation 17%, 3% and 3%. These are not a composite grade ≥3 TRAE rate.

Regimen (trial)	Safety denominator	Attribution rule as reported	Exposure context	Comparator and component contrast	Reported severe, serious and fatal events
Mitazalimab + mFOLFIRINOX (OPTIMIZE-1) [S34,S35]	70 treated; updated safety cutoff	Treatment-emergent	Until progression; first line	No concurrent control; single-arm combination	Updated report: grade ≥3 treatment-emergent adverse events in 55/70 (78.6%); neutropenia 18/70 (25.7%), hypokalemia 11/70 (15.7%), anemia 9/70 (12.9%) and thrombocytopenia 8/70 (11.4%). Serious adverse events in 30/70 (42.9%), including 8/70 (11.4%) considered mitazalimab-related; no serious adverse event leading to death. These updated figures supersede the earlier safety cutoff.
Quemliclustat + gem/nab-P ± zimberelimab (ARC-8) [S51]	61 with zimberelimab versus 29 without, at the shared quemliclustat/GnP dose	Study drug-related, by component	Until progression; first line	Randomized zimberelimab addition on a shared quemliclustat/GnP backbone; quemliclustat contribution not isolated	Composite study drug-related grade ≥3: 74% vs. 76%, dominated by chemotherapy; quemliclustat-attributable 23% vs. 21%; zimberelimab-attributable 28% vs. 0%
BMS-813160 + gem/nab-P ± nivolumab [S44]	35 triplet/35 BMS-813160–GnP/32 GnP safety-evaluable	Treatment-related (grade 3–5)	Until progression; first line	Randomized GnP-based comparison; nivolumab and BMS-813160 contributions depend on the arm contrast	Grade 3–5 treatment-related adverse events: 60.0% (+nivolumab), 85.7% (without nivolumab) and 68.8% (chemotherapy alone). Grade 3–5 treatment-related serious events: 20.0%, 20.0% and 6.3%; any-grade treatment-related serious events: 34.3%, 25.7% and 9.4%.
Durvalumab + tremelimumab vs. durvalumab [S72]	32 versus 32 treated and safety-evaluable; 65 randomized (32 versus 33). One patient assigned durvalumab received no treatment because of worsening disease.	Treatment-related	Up to 12 months or progression/unacceptable toxicity; after one prior fluorouracil- or gemcitabine-based regimen.	Randomized tremelimumab addition to durvalumab; no conventional chemotherapy control	Grade ≥3 treatment-related adverse events in 7/32 (22%) with combination versus 2/32 (6%) with durvalumab.
SBRT + nivolumab ± ipilimumab (CheckPAC) [S59]	41 SBRT/nivolumab versus 43 SBRT/nivolumab/ipilimumab treated	Treatment-related	Median four nivolumab cycles in each arm and two ipilimumab cycles; AEs monitored through 100 days after discontinuation	Randomized ipilimumab addition on SBRT/nivolumab; radiation contribution not isolated	Grade ≥3 TRAEs: 10/41 (24.4%) versus 13/43 (30.2%); no treatment-related death.
SBRT + ipilimumab/nivolumab/tocilizumab (TRIPLE-R) [S11]	26 treated	Treatment-related	Refractory disease; median follow-up 4.9 months; median drug exposure not established from accessed abstract	No concurrent control	Grade ≥3 TRAEs: 2/26 (8%); any-grade TRAEs: 19/26 (73%). Detailed event attribution beyond the abstract is unavailable.
Gem/nab-P + nivolumab/ipilimumab (REVOLUTION cohort A) [S33]	15 treated in cohort A	Treatment-related (grade 3–4)	Until progression; first line	No concurrent control for cohort A	Grade 3–4 TRAEs in 60%, as reported in the abstract; the body uses a grade ≥3 label. Cohort B safety is a separate regimen.

Regimen (trial)	Safety denominator	Attribution rule as reported	Exposure context	Comparator and component contrast	Reported severe, serious and fatal events
CRS-207 + nivolumab/ipilimumab ± GVAX [S76]	57 treated: 30 with GVAX/27 without	Treatment-related	Until progression; previously treated	Randomized GVAX addition on CRS-207/nivolumab/ipilimumab; no immune-free backbone control	Grade ≥3 AEs attributed to any study drug in 39/57 (68%), including one fatal immune-mediated myocarditis.
Acalabrutinib ± pembrolizumab [S66]	35 acalabrutinib versus 38 acalabrutinib/pembrolizumab treated	All-cause AEs and investigator-attributed treatment-related AEs reported separately	Median acalabrutinib exposure 1.4 months; pembrolizumab exposure 0.72 months; previously treated disease	Randomized pembrolizumab addition to acalabrutinib	Treatment-related grade 3–4 AEs: 5/35 (14.3%) versus 6/38 (15.8%). All-cause grade 3–4 AEs: 16/35 (45.7%) versus 28/38 (73.7%).
Oleclumab ± durvalumab + gem/nab-P [S65]	62 GnP/38 oleclumab–GnP/70 oleclumab–durvalumab–GnP treated in expansion	Treatment-related	Until progression; first line	Randomized same GnP backbone; the triplet adds two immune components	Grade ≥3 TRAEs: 67.7%/73.7%/77.1%; AE discontinuation: 11.3%/23.7%/24.3%. One control death was chemotherapy-related; one intracranial hemorrhage death was attributed to oleclumab/durvalumab in the triplet.
Niraparib + nivolumab vs. niraparib + ipilimumab [S22]	46 niraparib/nivolumab versus 45 niraparib/ipilimumab treated; includes two patients subsequently identified as neuroendocrine	Treatment-related	Continuous niraparib maintenance after platinum disease control; checkpoint schedules differed	Randomized checkpoint-partner comparison on niraparib; no niraparib-alone control	Grade 3–4 TRAEs: 10/46 (22%) versus 23/45 (printed as 50% in the accessed accepted manuscript; 23/45=51.1%). No treatment-related death. Final typeset correction of this percentage was not verified.
Pembrolizumab + olaparib (POLAR) [S41]	63 treated across cohorts A/B/C (33/15/15); includes four non-adenocarcinoma tumors	Treatment-related, with olaparib and pembrolizumab attribution separately tabulated	Maintenance; continuous dosing	No concurrent control; genomic cohorts are not randomized comparators	No grade 4–5 TRAEs reported. Grade 3 anemia differs between narrative/caption (10 patients) and Table 3 (13 patients, 29 events). Grade 3 immune-event counts and colitis grading also differ. A unique composite grade ≥3 patient rate cannot be established from these entries.
Cy/GVAX → CRS-207 ± nivolumab [S74]	51 with nivolumab versus 42 without, treated	Treatment-related	Six 3-week cycles; second line	Randomized nivolumab addition on the same Cy/GVAX–CRS-207 backbone	Grade ≥3 treatment-related AEs: 35.3% versus 11.9%; no treatment-related death reported.
Chemotherapy and chemoradiation ± algenpantucel-L [S20]	140 standard-treatment versus 142 algenpantucel-L safety-evaluable; 303 randomized	Grade ≥3 AEs; treatment attribution not specified in accessed primary abstract	Neoadjuvant; borderline resectable or locally advanced	Randomized algenpantucel-L addition on the same chemotherapy/chemoradiation backbone	Grade ≥3 AEs: 105/140 (75%) versus 115/142 (81%). Exact reasons for safety-set exclusions and detailed attribution were not verified from the accessible abstract.

**Regimen (trial)**	**Safety denominator**	**Attribution rule as reported**	**Exposure context**	**Comparator and component contrast**	**Reported severe, serious and fatal events**
Defactinib + pembrolizumab ± gemcitabine; triplet expansion [S37,S38]	Expansion table headed n=22; printed percentages imply n=23. Overall report n=42 includes mixed-tumor escalation and a predose withdrawal	Treatment-related grade 3–4 events; corrected heart-failure attribution	Dose escalation followed by refractory and post-GnP maintenance expansion; exposure differs by cohort	No concurrent control	Expansion table reports neutropenia 9 (39%) and anemia 4 (17%); the denominator conflict prevents an unqualified rate. No DLT. Correction: patient 01–046 heart failure unrelated to study drugs; defactinib attribution removed for a second patient.
Entinostat + nivolumab [S62]	30 received entinostat; safety includes three early toxicity discontinuations excluded from n=27 efficacy	Treatment-related	Entinostat lead-in followed by nivolumab; six entinostat dose reductions	No concurrent control	Grade ≥3 TRAEs: 19/30 (63%); no grade 5 AE; three early toxicity discontinuations.
Neoadjuvant mFOLFIRINOX + nivolumab [S43]	28 treated	Treatment-related	Neoadjuvant; fixed duration	No randomized concurrent control; external comparisons do not isolate nivolumab	The narrative reports 11% grade ≥3 treatment-related events, but Table 1 lists grade 3–4 neutropenia in 5/28 patients; an internally consistent overall rate cannot be extracted. No grade ≥3 event was attributed to nivolumab; no grade C postoperative pancreatic fistula occurred.
Neoadjuvant chemoradiotherapy ± pembrolizumab [S55]	24 pembrolizumab versus 13 control treated	At least possibly related to neoadjuvant therapy	Neoadjuvant; fixed duration	Randomized pembrolizumab addition on the same chemoradiotherapy backbone	Grade ≥3 events at least possibly related to therapy: 9/24 (38%) versus 4/13 (31%); one probable combination-related grade 3 transaminitis DLT.
Gemcitabine/capecitabine ± GV1001 (KG4/2015) [S7]	75 vaccine versus 67 pooled controls in the safety set; 142/148 total, not 148 randomized	Table 3 labels TRAEs; narrative uses grade ≥3 AEs for the same figures	Until progression; first line	Same chemotherapy backbone, but published control group pools randomized eotaxin-high and nonrandomized eotaxin-low patients	Grade ≥3 AEs: 58/75 (77.3%) versus 49/67 (73.1%), P=.562. Neutropenia 57.3%/50.8%, anemia 16.0%/13.4%, leukopenia 12.0%/9.0%, thrombocytopenia 9.3%/13.4%. AEs leading to death: two/four; these are not automatically treatment-related deaths.
Gemcitabine ± elpamotide (PEGASUS-PC) [S30]	100 elpamotide versus 53 placebo treated/FAS; includes three adenosquamous tumors	Treatment-related ("adverse events related to the study drug")	First line; median vaccine/placebo exposure 113.5/112.0 days and gemcitabine 113.0/106.0 days	Randomized elpamotide addition versus placebo on gemcitabine	No composite grade ≥3 rate is reported. Per-event drug-related grade 3–4: neutropenia 48.0% vs. 56.6%, leukopenia 31.0% vs. 43.4%, lymphopenia 25.0% vs. 24.5%, hemoglobin decreased 17.0% vs. 15.1%, thrombocytopenia 15.0% vs. 15.1%; injection-site ulceration 4.0% vs. 0%; no protocol-treatment-related deaths
Gemcitabine ± IMM-101 (IMAGE-1) [S52]	74 IMM-101/gemcitabine versus 35 gemcitabine treated	All-cause/treatment-emergent; attributed fatal events separately specified	Median 4.83 vs. 2.79 months on study; first line. Rates were exposure-adjusted by the investigators because time on study differed	Randomized IMM-101 addition on gemcitabine	57 of 74 (77%) vs. 26 of 35 (74%); asthenia 11% vs. 3%, neutropenia 18% vs. 17%, anemia 8% vs. 3%, biliary sepsis 5% vs. 0%; grade 5 events 19% vs. 14%, none considered study drug-related

**Regimen (trial)**	**Safety denominator**	**Attribution rule as reported**	**Exposure context**	**Comparator and component contrast**	**Reported severe, serious and fatal events**
Sintilimab + mFOLFIRINOX vs. mFOLFIRINOX (CISPD3) [S1]	53 sintilimab/mFOLFIRINOX versus 54 mFOLFIRINOX treated	Treatment-emergent	Until progression; first line	Randomized sintilimab addition on mFOLFIRINOX	Grade ≥3 treatment-emergent adverse events 84.9% (45/53) vs. 74.1% (40/54); grade ≥3 immune-related adverse events 5.7% (3/53) vs. 0
Surufatinib + camrelizumab + nab-paclitaxel/S-1 vs. gem/nab-P (NASCA) [S45]	45 experimental versus 45 GnP treated	Treatment-emergent in Table 3; the abstract labels the same figures treatment-related	Eight cycles then maintenance until progression; first line	Randomized treatment packages with different chemotherapy backbones; two additional agents in experimental arm	No composite grade ≥3 rate is reported. Highest per-event grade 3 + 4: decreased neutrophil count 33.3% vs. 35.6%, decreased white cell count 31.1% vs. 26.7%, decreased lymphocyte count 20.0% vs. 6.7%, elevated bilirubin 11.1% (5 of 45, all grade 3) vs. 4.4% (2 of 45, one grade 3 and one grade 4), peripheral neuropathy 11.1% vs. 0%. Immune-related adverse events in 10 of 45 (22.2%) experimental-arm patients, of which two were grade 3
CP-870,893 + gemcitabine [S4]	22 combination-treated in primary abstract; detailed agent-exposure and event-table denominators unverified	Primary abstract identifies a dose-limiting grade 4 event; detailed attribution rule not established	Gemcitabine weekly for three weeks; CP-870,893 on day 3 of each 28-day cycle; actual median exposure unverified	No concurrent control	One grade 4 cerebrovascular accident DLT at 0.2 mg/kg; CRS was the commonest AE and generally grade 1–2. Composite and detailed grade ≥3 event rates could not be verified from the accessed primary abstract.
Lenvatinib + pembrolizumab (LEAP-005 cohort G) [S67]	103 treated in PDAC cohort G	Treatment-related, investigator-determined	Median treatment duration 2.1 months; one or two prior regimens	No concurrent control	Grade 3–4 treatment-related adverse events 61 of 103 (59.2%): grade 3 53 (51.5%), grade 4 8 (7.8%), no grade 5. Hypertension was the commonest (grade ≥3 in 28.2%). Discontinuation for a treatment-related event in 11 (10.7%). Regardless of attribution, grade 3–5 clinically significant lenvatinib events occurred in 56 (54.4%), including four grade 5
Cy/GVAX + pembrolizumab + SBRT (J15237) [S88]	58 received study treatment; efficacy n=54 is separate	Treatment-related, attributed per agent (CTCAE v4.0)	Two neoadjuvant cycles plus SBRT, then up to two years of maintenance immunotherapy; locally advanced disease after induction chemotherapy	No randomized concurrent control; historical surgical cohort is not a toxicity control	Grade 3–4 treatment-related immune AEs in 7/58 (12%); included pneumonitis, diabetic ketoacidosis, colitis, elevated liver enzymes, nephritis and rash. No toxicity-related surgical delay reported. One preoperative cholangitis death was unrelated to study treatment.

**Regimen (trial)**	**Safety denominator**	**Attribution rule as reported**	**Exposure context**	**Comparator and component contrast**	**Reported severe, serious and fatal events**
BMS-813160 + nivolumab + gem/nab-P vs. gem/nab-P [S68]	8 GnP controls/7 initial investigational/25 expansion patients	Treatment-emergent, with per-agent attribution codes	Four neoadjuvant cycles or until progression; borderline resectable or locally advanced disease	Eight concurrent GnP controls in initial randomized phase; later 25-person expansion nonrandomized	No composite grade ≥3 rate is reported. Only grade 3 diarrhea (1 of 25) and grade 3 QTc prolongation (1 of 25) were attributed solely to BMS-813160; the authors state the expansion cohort had a similar frequency of grade 3–4 events to the control arm. Highest per-event grade 3–4 in expansion vs. control: anemia 24% vs. 38%, neutropenia 20% vs. 38%, fatigue 12% vs. 13%, ALT increase 12% vs. 13%, hypokalemia 12% vs. 25%. Ten of 32 investigational-arm patients did not complete treatment, including two COVID-19 deaths and one sudden death of indeterminate cause
Gem/nab-P + nivolumab [S15]	50 treated	Treatment-emergent	First-line 28-day cycles: GnP on days 1, 8 and 15; nivolumab on days 1 and 15. Actual median exposure unverified from accessed abstract	No concurrent control	Grade 3–4 TEAEs in 48/50 (96%); 18/50 discontinued for TEAEs. One grade 5 respiratory-failure event and one hepatitis DLT among six DLT-evaluable patients. Fatal-event drug attribution, composite union and SAE incidence were not verified from the accessed abstract.
Gemcitabine + tremelimumab [S31]	34 treated	Treatment-related	Maximum four 84-day cycles (amended to permit up to seven); median two tremelimumab infusions (range 1–7); first line	No concurrent control	No composite grade ≥3 rate is reported. Most frequent grade 3/4 toxicities were asthenia 11.8% and nausea 8.8%; grade 3/4 diarrhea in 2 patients (both Portion B); 11 patients had ≥1 serious adverse event, of which one (diarrhea with dehydration) was treatment-related and fatal
Gemcitabine + ipilimumab [S32]	21 treated	Treatment-related, as stated in the Safety paragraph introducing Table 3; the subsequent composite is described as grade 3 or higher adverse events.	Gemcitabine weekly for 7 of every 8 weeks with ipilimumab at weeks 1, 4, 7 and 10 then every 12 weeks; advanced disease, 67% previously treated	No concurrent control	The Safety paragraph reports grade 3 or higher adverse events in 16/21 patients (76%); 74% of such events were hematologic. Grade 3–4 anemia and leukopenia occurred in 48% each and neutropenia in 43%; grade 3 thrombocytopenia in 14%, and diarrhea, nausea and hypokalemia in 10% each.

Regimen (trial)	Safety denominator	Attribution rule as reported	Exposure context	Comparator and component contrast	Reported severe, serious and fatal events
Neoadjuvant/adjuvant selicrelumab ± gem/nab-P (CITN-11-01) [S46]	16 neoadjuvant recipients; 13 initiated adjuvant therapy, although adjuvant table retains n=16 header	Selicrelumab-related (chemotherapy-related toxicities tabulated separately)	Single neoadjuvant dose 2 weeks before surgery, then up to four adjuvant 28-day cycles; resectable disease	No randomized comparator for selicrelumab-related toxicity; chemotherapy events reported separately	No composite grade ≥3 rate is reported. Neoadjuvant selicrelumab-related: 1 grade 3 hyperglycemia and 1 grade 3 elevated liver function tests; cytokine-release syndrome in 10 of 16 (9 grade 1); no serious adverse events. Adjuvant selicrelumab-related: grade 3 fatigue in 2 patients, and hypertension, elevated AST/ALT and thrombocytopenia in 1 each; grade 4 pancreatitis in 1; three serious adverse events in two patients
Tislelizumab + hypofractionated radiotherapy + gem/nab-P (THAG) [S3]	56 in final report	Grade ≥3 AEs; attribution not specified in accessed primary abstract	Four preoperative 21-day cycles with concurrent radiotherapy during cycle 3, followed by surgery in eligible patients; conversion setting	No concurrent control	Grade ≥3 adverse events occurred in 33/56 patients (58.9%)
Penpulimab + anlotinib + gem/nab-P (PAAG) [S5]	66 treated	Treatment-related	Eight cycles of the quadruplet then penpulimab and anlotinib maintenance; first line	No concurrent control	Grade 3 or 4 treatment-related events in 26 of 66 (39.4%), most commonly neutropenia (n=14); no grade 5 treatment-related events; no grade 3 or 4 treatment-related event occurred during maintenance
Toripalimab + gem/nab-P [S53]	72 treated	Treatment-related	Until progression; first line. Median 6 chemotherapy cycles (range 1–34) and 5.5 immunotherapy cycles (range 1–37)	No concurrent control	Grade 3/4 treatment-related events in 38 of 72 (52.8%), most commonly neutropenia 15.3%, anemia 13.9%, leukopenia 8.3%; all 72 patients experienced ≥1 treatment-related event; 5 grade 3 immune-related events led to permanent toripalimab discontinuation
SHR-1701 + gem/nab-P [S54]	56 treated, including phase Ib participants	Treatment-related	Until progression; first line. Median exposure 18.4 weeks for SHR-1701 and 18.0 weeks for each of nab-paclitaxel and gemcitabine	No concurrent control	Grade 3 or worse treatment-related events in 27 of 56 (48.2%), most commonly decreased neutrophil count 25.0% and decreased white cell count 16.1%; serious treatment-related events in 9 (16.1%); grade ≥3 immune-related events in 7 (12.5%); one grade 5 treatment-related event (upper gastrointestinal hemorrhage, 1.8%)

Regimen (trial)	Safety denominator	Attribution rule as reported	Exposure context	Comparator and component contrast	Reported severe, serious and fatal events
Azacitidine + pembrolizumab [S69]	34 received azacitidine; 31 later received both drugs	Treatment-related	Azacitidine lead-in; pembrolizumab from week 3, continued until progression/toxicity; median drug exposure not established	No concurrent control	Grade ≥3 treatment-related events in 7 patients (21%), including 3 grade 5 events, one of which was immune-related encephalitis; treatment-related events of any grade in 20 patients (59%)
ARRY-382 + pembrolizumab, phase II pancreatic cohort [S70]	27 treated: 25 specified PDAC and two pancreatic cancers not further specified	Investigator-attributed ARRY-382-related events; not a whole-regimen high-grade rate	Until progression; ≥2L. Median exposure 1.1 months for ARRY-382 and 1.4 months for pembrolizumab in the PDAC cohort	No concurrent control; other tumor cohorts are not pancreatic safety comparators	ARRY-382-related grade ≥3 AEs: 7/27 (25.9%); ARRY-382-related SAEs: 3/27 (11.1%). Pooled all-cause grade 3–5 rates across other histologies do not establish a PDAC-specific whole-regimen rate.
Plerixafor + cemiplimab [S71]	21 treated	Treatment-related, attributed separately to cemiplimab and to AMD3100	Seven-day continuous plerixafor infusion in each 21-day cycle with cemiplimab; previously treated metastatic disease	No concurrent control	Grade 3 cemiplimab-related hepatitis in one and pericarditis in one; grade 3 plerixafor-related syncope in two. No composite severe-TRAE patient rate established by these event counts.
LOAd703 + indication-specific chemotherapy/gemcitabine conditioning (LOKON002) [S21]	41 treated across dose cohorts (3/12/26), including 29 pancreatic patients; grade counts pooled across histologies	LOAd703-related events and all-cause SAEs; registry severe grades are event counts	Up to eight biweekly intratumoral injections; AEs collected from first treatment through final follow-up at 50 weeks	No concurrent control	Registry: six grade 3 and one grade 4 LOAd703-related events, no grade 5-related event; these are not patient incidences. All-cause SAEs affected 35/41 patients (3/3, 12/12, 20/26). One AE leading to death was recorded without LOAd703 attribution.
KIF20A/VEGFR1/VEGFR2 peptide cocktail + gemcitabine (VENUS-PC) [S29]	67 treated: 38 HLA-matched/29 unmatched	Related to the study drug	Weekly vaccination for 8 weeks then every 2 weeks with gemcitabine 1000 mg/m² days 1, 8, 15 of a 28-day cycle, until progression; first line	All received vaccine/gemcitabine; HLA groups are nonrandomized host-genotype comparisons	Per-event grade 3–4 rates, matched/unmatched: neutropenia 47.4%/58.6%, leukopenia 34.2%/27.6%, thrombocytopenia 21.1%/3.4%, cholangitis 13.2%/10.3%, injection-site reaction 7.9%/3.4%. Anorexia: 6/38 versus 3/29; unmatched percentage is misprinted as 10.8%. No treatment-related death.
TG01/GM-CSF + adjuvant gemcitabine (CT TG01-01) [S90]	32 treated: 19 initial/13 modified schedule	Treatment-emergent, with separate investigator attribution to TG01, gemcitabine or all products	Vaccination from within 12 weeks of surgery through 2 years; up to six 28-day cycles of gemcitabine 1000 mg/m² days 1, 8, 15; adjuvant	No randomized control; initial and modified schedules enrolled sequentially	Sixty-one grade 3–4 TEAEs: grade 3 in 23 patients (55 events), grade 4 in five (six events); patient overlap prevents summing an overall incidence. Twenty SAEs in 11/32 patients. Three TG01-related serious allergic reactions in the initial cohort, including two grade 4 anaphylactic reactions; none in the modified cohort. No treatment-related death.

Regimen (trial)	Safety denominator	Attribution rule as reported	Exposure context	Comparator and component contrast	Reported severe, serious and fatal events
OCV-C01 + adjuvant gemcitabine [S91]	30 received at least one OCV-C01 dose and formed the safety set	Events related to gemcitabine/OCV-C01; agent-specific relationships described narratively	Weekly subcutaneous OCV-C01 for 48 weeks with gemcitabine 1000 mg/m² days 1, 8, 15 for 24 weeks; 18 months follow-up; adjuvant	Fifteen prospective nonrandomized HLA-incompatible gemcitabine controls; no comparable control safety estimate in the inspected report	Five of 30 stopped for treatment-related AEs: two grade 2 interstitial pneumonias, one grade 3 anaphylaxis, one grade 1 anaphylaxis-like reaction and one grade 4 neutropenia. No protocol-related death. Composite grade ≥3 incidence not established from the inspected extraction.
WT1 and/or MUC1 peptide-loaded DC vaccine + gem/nab-P or FOLFIRINOX [S92]	48 treated	All-cause treatment-emergent adverse events (CTCAE v4.0) during the treatment period, with vaccine-attributed events described separately	Vaccination every 2–4 weeks, median 9 doses (range 5–29), with concurrent gem/nab-P (median 7 cycles) or FOLFIRINOX (median 9 cycles); metastatic/relapsed disease	No randomized concurrent control; chemotherapy chosen by referring clinicians	No composite grade ≥3 rate is reported. Grade 3–4 rates by event: neutropenia 46%, leukocytopenia 44%, anemia 13%, thrombocytopenia 8%, febrile neutropenia 8%, anorexia 4%, fatigue 2%, ALT elevation 2%, AST elevation 2%. Adverse events directly attributed to vaccination were injection-site erythema/induration (100%) and OK-432-related mild fever (40%), all low grade; 17 patients (35%) required granulocyte colony-stimulating factor
Hypofractionated radiotherapy + PD-1 antibody + SOX, second line (Wang et al.) [S61]	23 in reported safety/response set; 25 enrolled, and a post-treatment scan was required	Treatment-related (TRAE), CTCAE v5.0	Concurrent radiotherapy with SOX and PD-1 antibody until progression; second line after first-line failure	No concurrent control	Grade 3–4 TRAEs in 7/23. Per-event counts conflict between narrative and Table 3; toxic epidermal necrolysis is listed as zero any-grade but one high-grade event. A single reconciled event-level profile is unavailable. No treatment-related death reported.
Allogeneic NK cells + gemcitabine/S-1 (Tan et al.) [S94]	25 for regimen safety; 24 received NK cells	Treatment-related, with chemotherapy attribution judged by physicians on timing and known toxicity profiles and NK-infusion-related events reported separately	Gemcitabine days 1 and 8 plus oral S-1 days 1–14 of a 21-day cycle, with a single NK infusion on days 14–21 during the first four cycles (median 4 infusions, range 1–13), then S-1 maintenance; first line	No concurrent control	Grade ≥3 regimen TRAEs in 14/25 (56%), predominantly chemotherapy-associated. Two patients had chemotherapy-related grade 4 neutropenia. All NK-infusion-related AEs were grade ≤2 among 24 recipients; no NK DLT or GVHD.
Adjuvant gemcitabine + autologous γδ T-cell transfer (Aoki et al.) [S8]	28 combination-treated; formal safety-set definition unavailable in accessed abstract	Narrative attribution to gemcitabine versus cellular therapy; formal causality rule unavailable	Adjuvant gemcitabine with repeated autologous γδ-cell infusions; exact delivered exposure not established from abstract	Twenty nonrandomized gemcitabine controls; allocation depended on ex vivo cell proliferation	No severe AE obviously related to γδ-cell therapy in the primary abstract. Composite grade ≥3 whole-regimen incidence and detailed control safety unavailable.

Regimen (trial)	Safety denominator	Attribution rule as reported	Exposure context	Comparator and component contrast	Reported severe, serious and fatal events
Adjuvant Cy/GVAX + SBRT + (m)FOLFIRINOX (Hill et al., NCT01595321/J1179) [S9]	19 overall safety (3/4/12); DLT-evaluable 18 (3/3/12); Cy/GVAX-specific n=12	Registry: DLTs regardless of attribution; Cy/GVAX-related grade ≥3 patient counts separately	First-two-cycle DLT window eight weeks; five Cy/GVAX priming doses around SBRT/six chemotherapy cycles, then six-monthly boosts; long-term AE collection	Sequential backbone cohorts, not concurrent randomized controls	Registry DLTs: cohort 1 neutropenia 3/3 and thrombocytopenia 2/3; cohort 2 none/3; cohort 3 diarrhea 1/12 and neutropenia 1/12. Grade ≥3 Cy/GVAX-related AEs in 2/12. All-cause SAEs: 3/3, 1/4 and 6/12.
Canakinumab + spartalizumab + gem/nab-P (SR1) [S6]	10 treated	Treatment-related	First-line phase Ib; DLT window 56 days; continued combination therapy until progression/toxicity	No randomized safety control; separate GnP cohort used for blood correlatives	Grade 3 or higher treatment-related events in 7 of 10 (70%), predominantly hematologic; one pneumonitis leading to spartalizumab discontinuation and one gastrointestinal bleed leading to discontinuation of all agents; no grade 5 events

Table S6 notes: Treatment-related and all-cause events, severe and serious events, and event counts and patient incidences are distinct. Follow the population and attribution stated in each row. Gem/nab-P, gemcitabine plus nab-paclitaxel; SBRT, stereotactic body radiotherapy; Cy, cyclophosphamide.

S9. Extended notes to the main-text tables

These notes expand the interpretation of the main tables without repeating their trial results.

S9.1. Extended note to Table 1

Table 1 separates intended component function from human clinical evidence. Experimental mechanisms are labeled as such. Design tiers do not confer clinical efficacy, and a class-level entry does not establish that every drug shares the same biological or clinical effect.

S9.2. Extended note to Table 2

Table 2 is a selected clinical comparison, with complete row-level detail in Table S5. A primary biological or feasibility endpoint is not a clinical efficacy endpoint. Same-backbone component attribution, historical comparisons, outcome-selected analyses, missing data, reporting discrepancies, publication status and mixed histology are considered separately under Section S5.

S9.3. Extended note to Table 3

Table 3 distinguishes established tumor-agnostic selection, clinical enrichment and exploratory research. Authorization under an assay-specific label does not establish a PDAC combination interaction. The PA.7 gene-status-hit rule is not interchangeable with functional HRD or a single pathogenic variant.

S9.4. Extended note to Table 4

Tables 4 and S6 preserve source-specific safety interpretation. A concurrent immune-containing comparator can isolate the toxicity of an added component; a regimen with several changed components answers a broader comparison. Disease setting, treatment history, exposure and evaluability vary, preventing cross-trial rankings. An unresolved event count limits that estimate rather than necessarily invalidating all safety information.

S9.5. Extended note to Table 6

Table 6 compares selected reviews' stated scope and contributions. It does not score earlier reviews for omission of later publications, infer absence from inaccessible full text or claim that the present heuristic is uniquely novel.

Supplementary References

The supplementary references support Tables S3–S6. Shared publications also appear in the manuscript list under a separate numbering scheme. Related analyses and correction notices are distinguished from new clinical cohorts.

S1. Fu, Q.; Chen, Y.; Huang, D.; Guo, C.; Zhang, X.; Xiao, W.; Xue, X.; Zhang, Q.; Li, X.; Gao, S.; et al. Sintilimab Plus Modified FOLFIRINOX in Metastatic or Recurrent Pancreatic Cancer: The Randomized Phase II CISPD3 Trial. *Ann. Surg. Oncol.* **2023**, *30*, 5071–5080. https://doi.org/10.1245/s10434-023-13383-w

S2. Wang, M.; Shi, S.B.; Qi, J.L.; Tang, X.Y.; Tian, J. S-1 plus CIK as second-line treatment for advanced pancreatic cancer. *Med. Oncol.* **2013**, *30*, 747. https://doi.org/10.1007/s12032-013-0747-9

S3. Ni, J.; Sun, Y.; Cheng, H.; Bai, X.; Tong, F.; Zhang, X.; Wang, X.; Mao, L.; Li, G.; Shen, S.; et al. Tislelizumab and Hypofractionated Radiotherapy plus Nab-Paclitaxel/Gemcitabine as Conversion Therapy for BRPC/LAPC: A Phase II Trial with Dynamic Biomarker Monitoring. *Clin. Cancer Res.* **2026**, *32*, 516–527. https://doi.org/10.1158/1078-0432.ccr-25-2461

S4. Beatty, G.L.; Torigian, D.A.; Chiorean, E.G.; Saboury, B.; Brothers, A.; Alavi, A.; Troxel, A.B.; Sun, W.; Teitelbaum, U.R.; Vonderheide, R.H.; et al. A Phase I Study of an Agonist CD40 Monoclonal Antibody (CP-870,893) in Combination with Gemcitabine in Patients with

Advanced Pancreatic Ductal Adenocarcinoma. *Clin. Cancer Res.* **2013**, *19*, 6286–6295. https://doi.org/10.1158/1078-0432.ccr-13-1320

S5. Sha, H.; Tong, F.; Ni, J.; Sun, Y.; Zhu, Y.; Qi, L.; Li, X.; Li, W.; Yang, Y.; Gu, Q.; et al. First-line penpulimab (an anti-PD1 antibody) and anlotinib (an angiogenesis inhibitor) with nab-paclitaxel/gemcitabine (PAAG) in metastatic pancreatic cancer: a prospective, multicentre, biomolecular exploratory, phase II trial. *Signal Transduct. Target. Ther.* **2024**, *9*, 143. https://doi.org/10.1038/s41392-024-01857-6

S6. Oberstein, P.E.; Dias Costa, A.; Kawaler, E.A.; Cardot-Ruffino, V.; Rahma, O.E.; Beri, N.; Singh, H.; Abrams, T.A.; Biller, L.H.; Cleary, J.M.; et al. Blockade of IL1β and PD1 with Combination Chemotherapy Reduces Systemic Myeloid Suppression in Metastatic Pancreatic Cancer with Heterogeneous Effects in the Tumor. *Cancer Immunol. Res.* **2024**, *12*, 1221–1235. https://doi.org/10.1158/2326-6066.cir-23-1073

S7. Jo, J.H.; Kim, Y.T.; Choi, H.S.; Kim, H.G.; Lee, H.S.; Choi, Y.W.; Kim, D.U.; Lee, K.H.; Kim, E.J.; Han, J.H.; et al. Efficacy of GV1001 with gemcitabine/capecitabine in previously untreated patients with advanced pancreatic ductal adenocarcinoma having high serum eotaxin levels (KG4/2015): an open-label, randomised, Phase 3 trial. *Br. J. Cancer* **2024**, *130*, 43–52. https://doi.org/10.1038/s41416-023-02474-w

S8. Aoki, T.; Matsushita, H.; Hoshikawa, M.; Hasegawa, K.; Kokudo, N.; Kakimi, K. Adjuvant combination therapy with gemcitabine and autologous γδ T-cell transfer in patients with curatively resected pancreatic cancer. *Cytotherapy* **2017**, *19*, 473–485. https://doi.org/10.1016/j.jcyt.2017.01.002

S9. Hill, C.S.; Parkinson, R.; Jaffee, E.M.; Sugar, E.; Zheng, L.; Onners, B.; Weiss, M.J.; Wolfgang, C.L.; Cameron, J.L.; Pawlik, T.M.; et al. Phase 1 Study of Adjuvant Allogeneic Granulocyte-Macrophage Colony-Stimulating Factor–Transduced Pancreatic Tumor Cell Vaccine, Low-Dose Cyclophosphamide, and Stereotactic Body Radiation Therapy Followed by FOLFIRINOX in High-Risk Resected Pancreatic Ductal Adenocarcinoma. *Int. J. Radiat. Oncol. Biol. Phys.* **2025**, *121*, 930–941. https://doi.org/10.1016/j.ijrobp.2024.10.039

S10. Du, J.; Lu, C.; Mao, L.; Zhu, Y.; Kong, W.; Shen, S.; Tang, M.; Bao, S.; Cheng, H.; Li, G.; et al. PD-1 blockade plus chemoradiotherapy as preoperative therapy for patients with BRPC/LAPC: A biomolecular exploratory, phase II trial. *Cell Rep. Med.* **2023**, *4*, 100972. https://doi.org/10.1016/j.xcrm.2023.100972

S11. Chen, I.M.; Donia, M.; Chamberlain, C.A.; Jensen, A.W.P.; Draghi, A.; Theile, S.; Madsen, K.; Hasselby, J.P.; Toxværd, A.; Høgdall, E.; et al. Phase 2 study of ipilimumab, nivolumab, and tocilizumab combined with stereotactic body radiotherapy in patients with refractory pancreatic cancer (TRIPLE-R). *Eur. J. Cancer* **2023**, *180*, 125–133. https://doi.org/10.1016/j.ejca.2022.11.035

S12. Pelletier, M.; Yang, J.; Joshi, M.; Grauel, A.; Abecunas, C.; Altzerinakou, M.A.; Zhou, Z.; Zhu, X.; Clark, K.; Li, L.; et al. Biomarker Analysis from Patients with Metastatic PDAC Treated with TGFβ Antibody NIS793 plus Abraxane + Gemcitabine versus Abraxane + Gemcitabine Alone in a Phase II, Open-Label, Randomized Study. *Clin. Cancer Res.* **2026**, *online ahead of print*, OF1–OF15. https://doi.org/10.1158/1078-0432.ccr-26-0805

S13. O'Hara, M.H.; O'Reilly, E.M.; Varadhachary, G.; Wolff, R.A.; Wainberg, Z.A.; Ko, A.H.; Fisher, G.; Rahma, O.; Lyman, J.P.; Cabanski, C.R.; et al. CD40 agonistic monoclonal antibody APX005M (sotigalimab) and chemotherapy, with or without nivolumab, for the treatment of metastatic pancreatic adenocarcinoma: an open-label, multicentre, phase 1b study. *Lancet Oncol.* **2021**, *22*, 118–131. https://doi.org/10.1016/s1470-2045(20)30532-5

S14. Mahalingam, D.; Wilkinson, G.A.; Eng, K.H.; Fields, P.; Raber, P.; Moseley, J.L.; Cheetham, K.; Coffey, M.; Nuovo, G.; Kalinski, P.; et al. Pembrolizumab in Combination with the Oncolytic Virus Pelareorep and Chemotherapy in Patients with Advanced Pancreatic

Adenocarcinoma: A Phase Ib Study. *Clin. Cancer Res.* **2020**, *26*, 71–81. https://doi.org/10.1158/1078-0432.ccr-19-2078

S15. Wainberg, Z.A.; Hochster, H.S.; Kim, E.J.; George, B.; Kaylan, A.; Chiorean, E.G.; Waterhouse, D.M.; Guiterrez, M.; Parikh, A.; Jain, R.; et al. Open-label, Phase I Study of Nivolumab Combined with nab-Paclitaxel Plus Gemcitabine in Advanced Pancreatic Cancer. *Clin. Cancer Res.* **2020**, *26*, 4814–4822. https://doi.org/10.1158/1078-0432.ccr-20-0099

S16. Bockorny, B.; Semenisty, V.; Macarulla, T.; Borazanci, E.; Wolpin, B.M.; Stemmer, S.M.; Golan, T.; Geva, R.; Borad, M.J.; Pedersen, K.S.; et al. BL-8040, a CXCR4 antagonist, in combination with pembrolizumab and chemotherapy for pancreatic cancer: the COMBAT trial. *Nat. Med.* **2020**, *26*, 878–885. https://doi.org/10.1038/s41591-020-0880-x

S17. Bockorny, B.; Macarulla, T.; Semenisty, V.; Borazanci, E.; Feliu, J.; Ponz-Sarvise, M.; Abad, D.G.; Oberstein, P.; Alistar, A.; Muñoz, A.; et al. Motixafortide and Pembrolizumab Combined to Nanoliposomal Irinotecan, Fluorouracil, and Folinic Acid in Metastatic Pancreatic Cancer: The COMBAT/KEYNOTE-202 Trial. *Clin. Cancer Res.* **2021**, *27*, 5020–5027. https://doi.org/10.1158/1078-0432.ccr-21-0929

S18. Middleton, G.; Silcocks, P.; Cox, T.; Valle, J.; Wadsley, J.; Propper, D.; Coxon, F.; Ross, P.; Madhusudan, S.; Roques, T.; et al. Gemcitabine and capecitabine with or without telomerase peptide vaccine GV1001 in patients with locally advanced or metastatic pancreatic cancer (TeloVac): an open-label, randomised, phase 3 trial. *Lancet Oncol.* **2014**, *15*, 829–840. https://doi.org/10.1016/s1470-2045(14)70236-0

S19. Hardacre, J.M.; Mulcahy, M.; Small, W.; Talamonti, M.; Obel, J.; Krishnamurthi, S.; Rocha-Lima, C.S.; Safran, H.; Lenz, H.J.; Chiorean, E.G. Addition of Algenpantucel-L Immunotherapy to Standard Adjuvant Therapy for Pancreatic Cancer: a Phase 2 Study. *J. Gastrointest. Surg.* **2013**, *17*, 94–101. https://doi.org/10.1007/s11605-012-2064-6

S20. Hewitt, D.B.; Nissen, N.; Hatoum, H.; Musher, B.; Seng, J.; Coveler, A.L.; Al-Rajabi, R.; Yeo, C.J.; Leiby, B.; Banks, J.; et al. A Phase 3 Randomized Clinical Trial of Chemotherapy With or Without Algenpantucel-L (HyperAcute-Pancreas) Immunotherapy in Subjects With Borderline Resectable or Locally Advanced Unresectable Pancreatic Cancer. *Ann. Surg.* **2022**, *275*, 45–53. https://doi.org/10.1097/SLA.0000000000004669

S21. Hahn, A.; Irenaeus, S.; Sandin, L.C.; Nordström, C.; Wenthe, J.; Lövgren, T.; Eriksson, E.; Alsaqal, S.; Pahnke, S.; Sundin, A.; et al. Tumor Microenvironment Gene Engineering with LOAd703 in Patients with Solid Malignancies: LOKON002 Phase I/IIb Clinical Trial. *Clin. Cancer Res.* **2026**, *32*, 3157–3168. https://doi.org/10.1158/1078-0432.ccr-25-4396

S22. Reiss, K.A.; Mick, R.; Teitelbaum, U.; O'Hara, M.; Schneider, C.; Massa, R.; Karasic, T.; Tondon, R.; Onyiah, C.; Gosselin, M.K.; et al. Niraparib plus nivolumab or niraparib plus ipilimumab in patients with platinum-sensitive advanced pancreatic cancer: a randomised, phase 1b/2 trial. *Lancet Oncol.* **2022**, *23*, 1009–1020. https://doi.org/10.1016/s1470-2045(22)00369-2

S23. Correction to Lancet Oncol 2022; 23: 1009–20. *Lancet Oncol.* **2022**, *23*, e446. https://doi.org/10.1016/s1470-2045(22)00571-x

S24. Hernando-Calvo, A.; Han, M.; Ayodele, O.; Wang, B.X.; Bruce, J.P.; Abbas-Aghababazadeh, F.; Vila-Casadesús, M.; Sanz-Garcia, E.; Yang, S.Y.C.; Berman, H.K.; et al. A Phase II, Open-Label, Randomized Trial of Durvalumab With Olaparib or Cediranib in Patients With Mismatch Repair—Proficient Colorectal or Pancreatic Cancer. *Clin. Colorectal Cancer* **2024**, *23*, 272–284.e9. https://doi.org/10.1016/j.clcc.2024.05.002

S25. Chung, V.; Guthrie, K.A.; Pishvaian, M.J.; Lowy, A.M.; Sohal, D.; Colby, S.; Reiss, K.A.; Khan, G.; Winograd, R.; Meyerhardt, J.A.; et al. Randomized phase II trial of olaparib and pembrolizumab vs. olaparib alone as maintenance therapy in metastatic pancreatic cancer patients with germline BRCA1 or BRCA2 (gBRCA1/2) mutations: SWOG S2001. *J. Clin.*

Oncol. **2026**, *44 (Suppl. 16)*, 4012, ASCO Annual Meeting Abstract 4012. https://doi.org/10.1200/JCO.2026.44.16_suppl.4012

S26. Callahan, M.; Amin, A.; Kaye, F.J.; Morse, M.A.; Taylor, M.H.; Peltola, K.J.; Sharma, P.; O'Reilly, E.M.; Meadows Shropshire, S.; O'Brien, S.; et al. Nivolumab monotherapy or combination with ipilimumab with or without cobimetinib in previously treated patients with pancreatic adenocarcinoma (CheckMate 032). *J. Immunother. Cancer* **2024**, *12*, e007883. https://doi.org/10.1136/jitc-2023-007883

S27. Weiss, G.J.; Blaydorn, L.; Beck, J.; Bornemann-Kolatzki, K.; Urnovitz, H.; Schütz, E.; Khemka, V. Phase Ib/II study of gemcitabine, nab-paclitaxel, and pembrolizumab in metastatic pancreatic adenocarcinoma. *Invest. New Drugs* **2018**, *36*, 96–102. https://doi.org/10.1007/s10637-017-0525-1

S28. Weiss, G.J.; Blaydorn, L.; Beck, J.; Bornemann-Kolatzki, K.; Urnovitz, H.; Schütz, E.; Khemka, V. Correction to: Phase Ib/II study of gemcitabine, nab-paclitaxel, and pembrolizumab in metastatic pancreatic adenocarcinoma. *Invest. New Drugs* **2019**, *37*, 797. https://doi.org/10.1007/s10637-019-00763-x

S29. Suzuki, N.; Hazama, S.; Iguchi, H.; Uesugi, K.; Tanaka, H.; Hirakawa, K.; Aruga, A.; Hatori, T.; Ishizaki, H.; Umeda, Y.; et al. Phase II clinical trial of peptide cocktail therapy for patients with advanced pancreatic cancer: VENUS-PC study. *Cancer Sci.* **2017**, *108*, 73–80. https://doi.org/10.1111/cas.13113

S30. Yamaue, H.; Tsunoda, T.; Tani, M.; Miyazawa, M.; Yamao, K.; Mizuno, N.; Okusaka, T.; Ueno, H.; Boku, N.; Fukutomi, A.; et al. Randomized phase II/III clinical trial of elpamotide for patients with advanced pancreatic cancer: PEGASUS-PC Study. *Cancer Sci.* **2015**, *106*, 883–890. https://doi.org/10.1111/cas.12674

S31. Aglietta, M.; Barone, C.; Sawyer, M.B.; Moore, M.J.; Miller, W.H.; Bagalà, C.; Colombi, F.; Cagnazzo, C.; Gioeni, L.; Wang, E.; et al. A phase I dose escalation trial of tremelimumab (CP-675,206) in combination with gemcitabine in chemotherapy-naive patients with metastatic pancreatic cancer. *Ann. Oncol.* **2014**, *25*, 1750–1755. https://doi.org/10.1093/annonc/mdu205

S32. Kamath, S.D.; Kalyan, A.; Kircher, S.; Nimeiri, H.; Fought, A.J.; Benson, A.; Mulcahy, M. Ipilimumab and Gemcitabine for Advanced Pancreatic Cancer: A Phase Ib Study. *Oncologist* **2020**, *25*, e808–e815. https://doi.org/10.1634/theoncologist.2019-0473

S33. O'Reilly, E.M.; Cabanski, C.R.; Lyman, J.P.; Wainberg, Z.A.; Fisher, G.A.; Wolff, R.A.; Ko, A.H.; O'Hara, M.H.; Spencer, C.N.; Yu, J.X.; et al. Clinical and translational results from a phase 1 trial of gemcitabine/nab-paclitaxel with nivolumab/ipilimumab or hydroxychloroquine/ipilimumab in untreated metastatic pancreatic adenocarcinoma. *J. Immunother. Cancer* **2026**, *14*, e012864. https://doi.org/10.1136/jitc-2025-012864

S34. Van Laethem, J.L.; Borbath, I.; Prenen, H.; Geboes, K.P.; Lambert, A.; Mitry, E.; Cassier, P.A.; Blanc, J.F.; Pilla, L.; Batlle, J.F.; et al. Combining CD40 agonist mitazalimab with mFOLFIRINOX in previously untreated metastatic pancreatic ductal adenocarcinoma (OPTIMIZE-1): a single-arm, multicentre phase 1b/2 study. *Lancet Oncol.* **2024**, *25*, 853–864. https://doi.org/10.1016/s1470-2045(24)00263-8

S35. Van Laethem, J.L.; Geboes, K.; Borbath, I.; Macarulla Mercade, T.; Lambert, A.; Cassier, P.; Prenen, H.; Mitry, E.; Blanc, J.F.; Pilla, L.; et al. CD40 agonist mitazalimab with mFOLFIRINOX in untreated metastatic pancreatic cancer: Biomarkers associated with outcomes from OPTIMIZE-1. *Cell Rep. Med.* **2025**, *6*, 102407. https://doi.org/10.1016/j.xcrm.2025.102407

S36. Musher, B.L.; Rowinsky, E.K.; Smaglo, B.G.; Abidi, W.; Othman, M.; Patel, K.; Jawaid, S.; Jing, J.; Brisco, A.; Leen, A.M.; et al. LOAd703, an oncolytic virus-based immunostimulatory gene therapy, combined with chemotherapy for unresectable or metastatic pancreatic

cancer (LOKON001): results from arm 1 of a non-randomised, single-centre, phase 1/2 study. *Lancet Oncol.* **2024**, *25*, 488–500. https://doi.org/10.1016/s1470-2045(24)00079-2

S37. Wang-Gillam, A.; Lim, K.H.; McWilliams, R.; Suresh, R.; Lockhart, A.C.; Brown, A.; Breden, M.; Belle, J.I.; Herndon, J.; Bogner, S.J.; et al. Defactinib, Pembrolizumab, and Gemcitabine in Patients with Advanced Treatment Refractory Pancreatic Cancer: A Phase I Dose Escalation and Expansion Study. *Clin. Cancer Res.* **2022**, *28*, 5254–5262. https://doi.org/10.1158/1078-0432.ccr-22-0308

S38. Wang-Gillam, A.; Lim, K.H.; McWilliams, R.; Suresh, R.; Lockhart, A.C.; Brown, A.; Breden, M.; Belle, J.I.; Herndon, J.; Bogner, S.J.; et al. Correction: Defactinib, Pembrolizumab, and Gemcitabine in Patients with Advanced Treatment Refractory Pancreatic Cancer: a Phase I Dose Escalation and Expansion Study. *Clin. Cancer Res.* **2023**, *29*, 4698. https://doi.org/10.1158/1078-0432.ccr-23-2993

S39. Manji, G.A.; Chung, V.; Oh, D.Y.; Lacy, J.; Lopez, C.D.; Ponz-Sarvisé, M.; Macarulla, T.; Thomas, R.; George, B.; Alistar, A.; et al. Atezolizumab and motixafortide, cobimetinib or simlukafusp alfa in pretreated advanced pancreatic cancer: phase I/IIb MORPHEUS-PDAC umbrella study. *Oncologist* **2026**, *31*, oyag023. https://doi.org/10.1093/oncolo/oyag023

S40. Ko, A.H.; Kim, K.P.; Siveke, J.T.; Lopez, C.D.; Lacy, J.; O'Reilly, E.M.; Macarulla, T.; Manji, G.A.; Lee, J.; Ajani, J.; et al. Atezolizumab Plus PEGPH20 Versus Chemotherapy in Advanced Pancreatic Ductal Adenocarcinoma and Gastric Cancer: MORPHEUS Phase Ib/II Umbrella Randomized Study Platform. *Oncologist* **2023**, *28*, 553–e472. https://doi.org/10.1093/oncolo/oyad022

S41. Park, W.; O'Connor, C.A.; Chou, J.F.; Hilmi, M.; Tarcan, Z.; Schwartz, C.; Larsen, M.; Homsi, R.; Sivaprakasam, K.; Umeda, S.; et al. Pembrolizumab and olaparib in homologous-recombination-deficient metastatic pancreatic cancer: the phase 2 POLAR trial. *Nat. Med.* **2026**, *32*, 1783–1793. https://doi.org/10.1038/s41591-026-04299-5

S42. Jo, J.H.; Kim, Y.T.; Choi, H.S.; Kim, H.G.; Lee, H.S.; Choi, Y.W.; Kim, D.U.; Lee, K.H.; Kim, E.J.; Han, J.H.; et al. Correction: Efficacy of GV1001 with gemcitabine/capecitabine in previously untreated patients with advanced pancreatic ductal adenocarcinoma having high serum eotaxin levels (KG4/2015): an open-label, randomised, Phase 3 trial. *Br. J. Cancer* **2024**, *130*, 163. https://doi.org/10.1038/s41416-023-02520-7

S43. Wainberg, Z.A.; Link, J.M.; Premji, A.; Zheng, S.; Srienc, M.; Hammons, M.; Kim, S.E.; Li, L.; Liu, Z.; Tsvetkova, O.; et al. Neoadjuvant modified FOLFIRINOX plus nivolumab in borderline-resectable pancreatic ductal adenocarcinoma: a pilot phase 1 trial. *Nat. Commun.* **2026**, *17*, 2232. https://doi.org/10.1038/s41467-026-68976-2

S44. Le, D.T.; Folprecht, G.; Varghese, A.M.; Gutierrez, M.; Noel, M.; Trikalinos, N.A.; Chen, E.; Dayyani, F.; Davis, S.L.; Ma, W.W.; et al. Phase 1b/2 study of BMS-813160, a CCR2/5 dual antagonist, in combination with chemotherapy or nivolumab in patients with advanced pancreatic or colorectal cancer. *J. Immunother. Cancer* **2026**, *14*, e011284. https://doi.org/10.1136/jitc-2024-011284

S45. Jia, R.; Si, H.Y.; Fan, M.J.; Zhang, N.; Deng, G.C.; Liu, F.F.; Han, L.; Gou, M.M.; Tan, Z.L.; Zhang, X.; et al. First-line treatment with chemotherapy, surufatinib (an angio-immuno kinase inhibitor), and camrelizumab (an anti-PD-1 antibody) for locally advanced or metastatic pancreatic ductal adenocarcinoma: a phase Ib/II randomized study. *Signal Transduct. Target. Ther.* **2025**, *10*, 339. https://doi.org/10.1038/s41392-025-02441-2

S46. Byrne, K.T.; Betts, C.B.; Mick, R.; Sivagnanam, S.; Bajor, D.L.; Laheru, D.A.; Chiorean, E.G.; O'Hara, M.H.; Liudahl, S.M.; Newcomb, C.; et al. Neoadjuvant Selicrelumab, an Agonist CD40 Antibody, Induces Changes in the Tumor Microenvironment in Patients with Resectable Pancreatic Cancer. *Clin. Cancer Res.* **2021**, *27*, 4574–4586. https://doi.org/10.1158/1078-0432.ccr-21-1047

S47. Renouf, D.J.; Loree, J.M.; Knox, J.J.; Topham, J.T.; Kavan, P.; Jonker, D.; Welch, S.; Couture, F.; Lemay, F.; Tehfe, M.; et al. The CCTG PA.7 phase II trial of gemcitabine and nab-paclitaxel with or without durvalumab and tremelimumab as initial therapy in metastatic pancreatic ductal adenocarcinoma. *Nat. Commun.* **2022**, *13*, 5020. https://doi.org/10.1038/s41467-022-32591-8

S48. Renouf, D.J.; Topham, J.T.; Loree, J.M.; Schaeffer, D.F.; Knox, J.J.; Kavan, P.; Jonker, D.; Welch, S.; Couture, F.; Lemay, F.; et al. DNA Repair gene alterations and efficacy from gemcitabine and nab-paclitaxel with/without durvalumab and tremelimumab in metastatic pancreatic ductal adenocarcinoma. *Nat. Commun.* **2026**, *17*, 3631. https://doi.org/10.1038/s41467-026-70120-z

S49. Padrón, L.J.; Maurer, D.M.; O'Hara, M.H.; O'Reilly, E.M.; Wolff, R.A.; Wainberg, Z.A.; Ko, A.H.; Fisher, G.; Rahma, O.; Lyman, J.P.; et al. Sotigalimab and/or nivolumab with chemotherapy in first-line metastatic pancreatic cancer: clinical and immunologic analyses from the randomized phase 2 PRINCE trial. *Nat. Med.* **2022**, *28*, 1167–1177. https://doi.org/10.1038/s41591-022-01829-9

S50. Morizane, C.; Ueno, M.; Ikeda, M.; Sudo, K.; Hirashima, Y.; Kuroda, M.; Ueno, S.; Okusaka, T.; Furuse, J. A Phase 2 study of nivolumab in combination with modified FOLFIRINOX for metastatic pancreatic cancer. *BJC Rep.* **2024**, *2*, 3. https://doi.org/10.1038/s44276-023-00028-4

S51. Wainberg, Z.A.; Manji, G.A.; Bahary, N.; Ulahannan, S.V.; Pant, S.; Spigel, D.R.; Uboha, N.V.; Oberstein, P.E.; Saeed, A.; Beagle, B.; et al. Quemliclustat and chemotherapy with or without zimberelimab in metastatic pancreatic adenocarcinoma: a randomized phase 1 trial. *Nat. Med.* **2026**, *32*, 1267–1277. https://doi.org/10.1038/s41591-026-04283-z

S52. Dalgleish, A.G.; Stebbing, J.; Adamson, D.J.A.; Arif, S.S.; Bidoli, P.; Chang, D.; Cheeseman, S.; Diaz-Beveridge, R.; Fernandez-Martos, C.; Glynne-Jones, R.; et al. Randomised, open-label, phase II study of gemcitabine with and without IMM-101 for advanced pancreatic cancer. *Br. J. Cancer* **2016**, *115*, 789–796. https://doi.org/10.1038/bjc.2016.271 Erratum in Br. J. Cancer 2016, 115, e16. https://doi.org/10.1038/bjc.2016.342.

S53. Cheng, K.; Li, X.; Lv, W.; Zhao, G.; Zhou, R.; Chang, C.; Yang, H.; Li, R.; Li, Z.; Chen, Y.; et al. Spatial interactions of immune cells as potential predictors to efficacy of toripalimab plus chemotherapy in locally advanced or metastatic pancreatic ductal adenocarcinoma: a phase Ib/II trial. *Signal Transduct. Target. Ther.* **2024**, *9*, 321. https://doi.org/10.1038/s41392-024-02031-8

S54. Xue, R.; Wei, M.; Yuan, J.; Li, Z.; Zhou, Y.; Xue, Z.; Wu, Y.; Han, H.; Zhou, J.; Yu, X.; et al. A phase 1b/2 study of first-line anti-PD-L1/TGF-βRII fusion protein SHR-1701 combined with nab-paclitaxel and gemcitabine for advanced pancreatic ductal adenocarcinoma. *Signal Transduct. Target. Ther.* **2025**, *10*, 415. https://doi.org/10.1038/s41392-025-02530-2

S55. Katz, M.H.G.; Petroni, G.R.; Bauer, T.; Reilley, M.J.; Wolpin, B.M.; Stucky, C.C.; Bekaii-Saab, T.S.; Elias, R.; Merchant, N.; Dias Costa, A.; et al. Multicenter randomized controlled trial of neoadjuvant chemoradiotherapy alone or in combination with pembrolizumab in patients with resectable or borderline resectable pancreatic adenocarcinoma. *J. Immunother. Cancer* **2023**, *11*, e007586. https://doi.org/10.1136/jitc-2023-007586

S56. Xie, C.; Duffy, A.G.; Brar, G.; Fioravanti, S.; Mabry-Hrones, D.; Walker, M.; Bonilla, C.M.; Wood, B.J.; Citrin, D.E.; Gil Ramirez, E.M.; et al. Immune Checkpoint Blockade in Combination with Stereotactic Body Radiotherapy in Patients with Metastatic Pancreatic Ductal Adenocarcinoma. *Clin. Cancer Res.* **2020**, *26*, 2318–2326. https://doi.org/10.1158/1078-0432.ccr-19-3624

S57. Xie, C.; Duffy, A.G.; Brar, G.; Fioravanti, S.; Mabry-Hrones, D.; Walker, M.; Bonilla, C.M.; Wood, B.J.; Citrin, D.E.; Gil Ramirez, E.M.; et al. Correction: Immune Checkpoint Blockade in Combination with Stereotactic Body Radiotherapy in Patients with Metastatic Pancreatic

Ductal Adenocarcinoma. *Clin. Cancer Res.* **2021**, *27*, 358. https://doi.org/10.1158/1078-0432.ccr-20-4640

S58. Parikh, A.R.; Szabolcs, A.; Allen, J.N.; Clark, J.W.; Wo, J.Y.; Raabe, M.; Thel, H.; Hoyos, D.; Mehta, A.; Arshad, S.; et al. Radiation therapy enhances immunotherapy response in microsatellite stable colorectal and pancreatic adenocarcinoma in a phase II trial. *Nat. Cancer* **2021**, *2*, 1124–1135. https://doi.org/10.1038/s43018-021-00269-7

S59. Chen, I.M.; Johansen, J.S.; Theile, S.; Hjaltelin, J.X.; Novitski, S.I.; Brunak, S.; Hasselby, J.P.; Willemoe, G.L.; Lorentzen, T.; Madsen, K.; et al. Randomized Phase II Study of Nivolumab With or Without Ipilimumab Combined With Stereotactic Body Radiotherapy for Refractory Metastatic Pancreatic Cancer (CheckPAC). *J. Clin. Oncol.* **2022**, *40*, 3180–3189. https://doi.org/10.1200/JCO.21.02511

S60. Vošmik, M.; John, S.; Dvořák, J.; Mohelníková-Duchoňová, B.; Melichar, B.; Lohynská, R.; Ryška, A.; Banni, A.M.; Krempová, J.; Sirák, I. Stereotactic Radiotherapy Plus Nivolumab in Patients with Locally Advanced Pancreatic Cancer: Results from Phase 1/2 Clinical CA209-9KH Trial. *Oncol. Ther.* **2024**, *12*, 817–831. https://doi.org/10.1007/s40487-024-00309-z

S61. Wang, Q.; Tong, F.; Qiao, L.; Qi, L.; Sun, Y.; Zhu, Y.; Ni, J.; Liu, J.; Kong, W.; Liu, B.; et al. Hypofractionated radiotherapy plus PD-1 antibody and SOX chemotherapy as second-line therapy in metastatic pancreatic cancer: a single-arm, phase II clinical trial. *Cancer Immunol. Immunother.* **2024**, *73*, 201. https://doi.org/10.1007/s00262-024-03744-z

S62. Baretti, M.; Danilova, L.; Durham, J.N.; Betts, C.B.; Cope, L.; Sidiropoulos, D.N.; Tandurella, J.A.; Charmsaz, S.; Gross, N.; Hernandez, A.; et al. Entinostat in combination with nivolumab in metastatic pancreatic ductal adenocarcinoma: a phase 2 clinical trial. *Nat. Commun.* **2024**, *15*, 9801. https://doi.org/10.1038/s41467-024-52528-7

S63. Melisi, D.; Oh, D.Y.; Hollebecque, A.; Calvo, E.; Varghese, A.; Borazanci, E.; Macarulla, T.; Merz, V.; Zecchetto, C.; Zhao, Y.; et al. Safety and activity of the TGFβ receptor I kinase inhibitor galunisertib plus the anti-PD-L1 antibody durvalumab in metastatic pancreatic cancer. *J. Immunother. Cancer* **2021**, *9*, e002068. https://doi.org/10.1136/jitc-2020-002068

S64. Melisi, D.; Garcia-Carbonero, R.; Macarulla, T.; Pezet, D.; Deplanque, G.; Fuchs, M.; Trojan, J.; Oettle, H.; Kozloff, M.; Cleverly, A.; et al. Galunisertib plus gemcitabine vs. gemcitabine for first-line treatment of patients with unresectable pancreatic cancer. *Br. J. Cancer* **2018**, *119*, 1208–1214. https://doi.org/10.1038/s41416-018-0246-z

S65. Coveler, A.L.; Reilley, M.J.; Zalupski, M.; Macarulla, T.; Fountzilas, C.; Ponz-Sarvisé, M.; Nagrial, A.; Uboha, N.V.; Frentzas, S.; Overman, M.; et al. A Phase Ib/II Randomized Clinical Trial of Oleclumab with or without Durvalumab plus Chemotherapy in Patients with Metastatic Pancreatic Ductal Adenocarcinoma. *Clin. Cancer Res.* **2024**, *30*, 4609–4617. https://doi.org/10.1158/1078-0432.ccr-24-0499

S66. Overman, M.; Javle, M.; Davis, R.E.; Vats, P.; Kumar-Sinha, C.; Xiao, L.; Mettu, N.B.; Parra, E.R.; Benson, A.B.; Lopez, C.D.; et al. Randomized phase II study of the Bruton tyrosine kinase inhibitor acalabrutinib, alone or with pembrolizumab in patients with advanced pancreatic cancer. *J. Immunother. Cancer* **2020**, *8*, e000587. https://doi.org/10.1136/jitc-2020-000587

S67. Ponz-Sarvisé, M.; Rha, S.Y.; Gomez-Roca, C.A.; Ortega Morán, L.; Gill, S.; Tortora, G.; Geva, R.; Saada-Bouzid, E.; Santoro, A.; Kim, T.W.; et al. Lenvatinib plus Pembrolizumab for Patients with Previously Treated Advanced Gastric, Biliary Tract, or Pancreatic Cancer: Results from the Phase II LEAP-005 Study. *Cancer Res. Commun.* **2026**, *6*, 673–686. https://doi.org/10.1158/2767-9764.crc-26-0018

S68. Grierson, P.M.; Wolf, C.; Suresh, R.; Wang-Gillam, A.; Tan, B.R.; Ratner, L.; Oppelt, P.; Aranha, O.; Frith, A.; Pedersen, K.S.; et al. Neoadjuvant BMS-813160, Nivolumab,

Gemcitabine, and Nab-Paclitaxel for Patients with Pancreatic Cancer. *Clin. Cancer Res.* **2025**, *31*, 3644–3651. https://doi.org/10.1158/1078-0432.ccr-24-1821

S69. Safyan, R.A.; White, R.A.; Gonda, T.A.; Lee, S.M.; Han, J.; Kuriakose, N.; Yamamoto, N.K.; Kugel, S.; Jamison, J.K.; Manji, G.A.; et al. Phase 2 study of azacitidine plus pembrolizumab as second-line treatment in patients with locally advanced or metastatic pancreatic ductal adenocarcinoma. *Oncologist* **2026**, *31*, oyag091. https://doi.org/10.1093/oncolo/oyag091

S70. Johnson, M.; Dudek, A.Z.; Sukari, A.; Call, J.; Kunk, P.R.; Lewis, K.; Gainor, J.F.; Sarantopoulos, J.; Lee, P.; Golden, A.; et al. ARRY-382 in Combination with Pembrolizumab in Patients with Advanced Solid Tumors: Results from a Phase 1b/2 Study. *Clin. Cancer Res.* **2022**, *28*, 2517–2526. https://doi.org/10.1158/1078-0432.ccr-21-3009

S71. Bever, K.M.; Shin, S.M.; Durham, J.N.; Qi, H.; Hernandez, A.; Coyne, E.M.; Gross, N.E.; Charmsaz, S.; Suresh Babu, J.; Vargas Carvajal, D.C.; et al. A phase 2 trial of CXCR4 antagonism and PD1 inhibition in metastatic pancreatic adenocarcinoma reveals recruitment of T cells but also immunosuppressive macrophages. *Oncoimmunology* **2025**, *14*, 2543614. https://doi.org/10.1080/2162402X.2025.2543614

S72. O'Reilly, E.M.; Oh, D.Y.; Dhani, N.; Renouf, D.J.; Lee, M.A.; Sun, W.; Fisher, G.; Hezel, A.; Chang, S.C.; Vlahovic, G.; et al. Durvalumab With or Without Tremelimumab for Patients With Metastatic Pancreatic Ductal Adenocarcinoma: A Phase 2 Randomized Clinical Trial. *JAMA Oncol.* **2019**, *5*, 1431–1438. https://doi.org/10.1001/jamaoncol.2019.1588

S73. Le, D.T.; Wang-Gillam, A.; Picozzi, V.; Greten, T.F.; Crocenzi, T.; Springett, G.; Morse, M.; Zeh, H.; Cohen, D.; Fine, R.L.; et al. Safety and Survival With GVAX Pancreas Prime and Listeria Monocytogenes–Expressing Mesothelin (CRS-207) Boost Vaccines for Metastatic Pancreatic Cancer. *J. Clin. Oncol.* **2015**, *33*, 1325–1333. https://doi.org/10.1200/JCO.2014.57.4244

S74. Tsujikawa, T.; Crocenzi, T.; Durham, J.N.; Sugar, E.A.; Wu, A.A.; Onners, B.; Nauroth, J.M.; Anders, R.A.; Fertig, E.J.; Laheru, D.A.; et al. Evaluation of Cyclophosphamide/GVAX Pancreas Followed by Listeria-Mesothelin (CRS-207) with or without Nivolumab in Patients with Pancreatic Cancer. *Clin. Cancer Res.* **2020**, *26*, 3578–3588. https://doi.org/10.1158/1078-0432.ccr-19-3978

S75. Le, D.T.; Picozzi, V.J.; Ko, A.H.; Wainberg, Z.A.; Kindler, H.; Wang-Gillam, A.; Oberstein, P.; Morse, M.A.; Zeh, H.J.; Weekes, C.; et al. Results from a Phase IIb, Randomized, Multicenter Study of GVAX Pancreas and CRS-207 Compared with Chemotherapy in Adults with Previously Treated Metastatic Pancreatic Adenocarcinoma (ECLIPSE Study). *Clin. Cancer Res.* **2019**, *25*, 5493–5502. https://doi.org/10.1158/1078-0432.ccr-18-2992

S76. Bever, K.M.; Huff, A.L.; Danilova, L.; Wetzel, M.; Tandurella, J.A.; Durham, J.N.; Wang, T.; Lee, J.W.; Coyne, E.M.; Montagne, J.M.; et al. A Randomized Phase II Study: CRS207/GVAX plus Anti–PD-1 and Anti-CTLA4 Recruits Mesothelin- and mKRAS-Specific T Cells into PDAC. *Cancer Immunol. Res.* **2026**, *14*, 411–422. https://doi.org/10.1158/2326-6066.cir-25-0545

S77. Wu, A.A.; Bever, K.M.; Ho, W.J.; Fertig, E.J.; Niu, N.; Zheng, L.; Parkinson, R.M.; Durham, J.N.; Onners, B.; Ferguson, A.K.; et al. A Phase II Study of Allogeneic GM-CSF–Transfected Pancreatic Tumor Vaccine (GVAX) with Ipilimumab as Maintenance Treatment for Metastatic Pancreatic Cancer. *Clin. Cancer Res.* **2020**, *26*, 5129–5139. https://doi.org/10.1158/1078-0432.ccr-20-1025

S78. Le, D.T.; Lutz, E.; Uram, J.N.; Sugar, E.A.; Onners, B.; Solt, S.; Zheng, L.; Diaz, L.A.; Donehower, R.C.; Jaffee, E.M.; et al. Evaluation of Ipilimumab in Combination With Allogeneic Pancreatic Tumor Cells Transfected With a GM-CSF Gene in Previously Treated Pancreatic Cancer. *J. Immunother.* **2013**, *36*, 382–389. https://doi.org/10.1097/cji.0b013e31829fb7a2

S79. Agarwal, P.; Guo, M.; Munjal, K.; Qi, H.; Parkinson, R.; Ferguson, A.; Mitchell, C.; Harrison, J.; Anders, R.A.; Thompson, E.D.; et al. A Phase II Study of Neoadjuvant GVAX and Cyclophosphamide Combined with Nivolumab and SBRT Followed by Surgery in Borderline Resectable Pancreatic Adenocarcinoma. *Clin. Cancer Res.* **2025**, *31*, 3205–3214. https://doi.org/10.1158/1078-0432.ccr-24-3403

S80. Heumann, T.; Judkins, C.; Li, K.; Lim, S.J.; Hoare, J.; Parkinson, R.; Cao, H.; Zhang, T.; Gai, J.; Celiker, B.; et al. A platform trial of neoadjuvant and adjuvant antitumor vaccination alone or in combination with PD-1 antagonist and CD137 agonist antibodies in patients with resectable pancreatic adenocarcinoma. *Nat. Commun.* **2023**, *14*, 3650. https://doi.org/10.1038/s41467-023-39196-9

S81. Rojas, L.A.; Sethna, Z.; Soares, K.C.; Olcese, C.; Pang, N.; Patterson, E.; Lihm, J.; Ceglia, N.; Guasp, P.; Chu, A.; et al. Personalized RNA neoantigen vaccines stimulate T cells in pancreatic cancer. *Nature* **2023**, *618*, 144–150. https://doi.org/10.1038/s41586-023-06063-y

S82. Sethna, Z.; Guasp, P.; Reiche, C.; Milighetti, M.; Ceglia, N.; Patterson, E.; Lihm, J.; Payne, G.; Lyudovyk, O.; Rojas, L.A.; et al. RNA neoantigen vaccines prime long-lived CD8+ T cells in pancreatic cancer. *Nature* **2025**, *639*, 1042–1051. https://doi.org/10.1038/s41586-024-08508-4

S83. Huff, A.L.; Haldar, S.D.; Girgis, A.A.; Wang, H.H.; Danilova, L.; Heumann, T.; Berg, M.; Wang, Y.; Andaloori, L.; Hernandez, A.; et al. Mutant KRAS vaccine with dual checkpoint blockade in resected pancreatic cancer: a phase I trial. *Nat. Commun.* **2026**, *17*, 1538. https://doi.org/10.1038/s41467-026-68324-4

S84. Huff, A.L.; Haldar, S.D.; Girgis, A.A.; Wang, H.H.; Danilova, L.; Heumann, T.; Berg, M.; Wang, Y.; Andaloori, L.; Hernandez, A.; et al. Publisher Correction: Mutant KRAS vaccine with dual checkpoint blockade in resected pancreatic cancer: a phase I trial. *Nat. Commun.* **2026**, *17*, 3516. https://doi.org/10.1038/s41467-026-71968-x

S85. Noonan, A.M.; Farren, M.R.; Geyer, S.M.; Huang, Y.; Tahiri, S.; Ahn, D.; Mikhail, S.; Ciombor, K.K.; Pant, S.; Aparo, S.; et al. Randomized Phase 2 Trial of the Oncolytic Virus Pelareorep (Reolysin) in Upfront Treatment of Metastatic Pancreatic Adenocarcinoma. *Mol. Ther.* **2016**, *24*, 1150–1158. https://doi.org/10.1038/mt.2016.66

S86. Nishida, S.; Ishikawa, T.; Egawa, S.; Koido, S.; Yanagimoto, H.; Ishii, J.; Kanno, Y.; Kokura, S.; Yasuda, H.; Oba, M.S.; et al. Combination Gemcitabine and WT1 Peptide Vaccination Improves Progression-Free Survival in Advanced Pancreatic Ductal Adenocarcinoma: A Phase II Randomized Study. *Cancer Immunol. Res.* **2018**, *6*, 320–331. https://doi.org/10.1158/2326-6066.cir-17-0386

S87. Shima, H.; Tsurita, G.; Wada, S.; Hirohashi, Y.; Yasui, H.; Hayashi, H.; Miyakoshi, T.; Watanabe, K.; Murai, A.; Asanuma, H.; et al. Randomized phase II trial of survivin 2B peptide vaccination for patients with HLA-A24-positive pancreatic adenocarcinoma. *Cancer Sci.* **2019**, *110*, 2378–2385. https://doi.org/10.1111/cas.14106

S88. Lee, V.; Sachidanand, A.S.; Rodriguez, C.; Wang, J.; Onners, B.; Qi, H.; Parkinson, R.; McPhaul, T.; Durham, J.; Ding, D.; et al. The combination of a cancer vaccine, pembrolizumab, and stereotactic body radiation in patients with locally advanced pancreatic cancer: a single-arm, phase II study. *Nat. Commun.* **2026**, *17*, 4141. https://doi.org/10.1038/s41467-026-69294-3

S89. Wang, J.; Gai, J.; Zhang, T.; Niu, N.; Qi, H.; Thomas, D.L.; Li, K.; Xia, T.; Rodriguez, C.; Parkinson, R.; et al. Neoadjuvant radioimmunotherapy in pancreatic cancer enhances effector T cell infiltration and shortens their distances to tumor cells. *Sci. Adv.* **2024**, *10*, eadk1827. https://doi.org/10.1126/sciadv.adk1827

S90. Palmer, D.H.; Valle, J.W.; Ma, Y.T.; Faluyi, O.; Neoptolemos, J.P.; Jensen Gjertsen, T.; Iversen, B.; Amund Eriksen, J.; Møller, A.S.; Aksnes, A.K.; et al. TG01/GM-CSF and adjuvant

gemcitabine in patients with resected RAS-mutant adenocarcinoma of the pancreas (CT TG01-01): a single-arm, phase 1/2 trial. *Br. J. Cancer* **2020**, *122*, 971–977. https://doi.org/10.1038/s41416-020-0752-7

S91. Miyazawa, M.; Katsuda, M.; Maguchi, H.; Katanuma, A.; Ishii, H.; Ozaka, M.; Yamao, K.; Imaoka, H.; Kawai, M.; Hirono, S.; et al. Phase II clinical trial using novel peptide cocktail vaccine as a postoperative adjuvant treatment for surgically resected pancreatic cancer patients. *Int. J. Cancer* **2017**, *140*, 973–982. https://doi.org/10.1002/ijc.30510

S92. Ota, S.; Miyashita, M.; Yamagishi, Y.; Ogasawara, M. Baseline immunity predicts prognosis of pancreatic cancer patients treated with WT1 and/or MUC1 peptide-loaded dendritic cell vaccination and a standard chemotherapy. *Hum. Vaccin. Immunother.* **2021**, *17*, 5563–5572. https://doi.org/10.1080/21645515.2021.2003645

S93. Lin, M.; Zhang, X.; Liang, S.; Luo, H.; Alnaggar, M.; Liu, A.; Yin, Z.; Chen, J.; Niu, L.; Jiang, Y. Irreversible electroporation plus allogenic Vγ9Vδ2 T cells enhances antitumor effect for locally advanced pancreatic cancer patients. *Signal Transduct. Target. Ther.* **2020**, *5*, 215. https://doi.org/10.1038/s41392-020-00260-1

S94. Tan, Q.; Li, Y.; Liu, C.; Xu, J.; Tong, J.; Yu, J.; Huang, Y.; Hu, X.; Qin, S.; Xiao, F.; et al. Dynamic transcriptional immune landscape in response to NK-cell therapy combined with gemcitabine plus S-1 in advanced pancreatic cancer: a phase 1b/2 trial. *Signal Transduct. Target. Ther.* **2025**, *10*, 381. https://doi.org/10.1038/s41392-025-02488-1

S95. Enzler, T.; Nguyen, A.; Misleh, J.; Cline, V.J.; Johns, M.; Shumway, N.; Paulson, S.; Siegel, R.; Larson, T.; Messersmith, W.; et al. A multicenter, randomized phase 2 study to establish combinations of CBP501, cisplatin and nivolumab for ≥3rd-line treatment of patients with advanced pancreatic adenocarcinoma. *Eur. J. Cancer* **2024**, *201*, 113950. https://doi.org/10.1016/j.ejca.2024.113950
